# New causal discovery algorithm over censored variables identifies subtype-specific drivers of breast cancer progression

**DOI:** 10.1093/gigascience/giag060

**Published:** 2026-05-22

**Authors:** Tyler C Lovelace, Panayiotis V Benos

**Affiliations:** Department of Computational & Systems Biology, University of Pittsburgh School of Medicine, 3396 Fifth Avenue, Pittsburgh, PA 15213, USA; Joint CMU-Pitt PhD Program in Computational Biology, Pittsburgh, PA 15213, USA; Department of Computational & Systems Biology, University of Pittsburgh School of Medicine, 3396 Fifth Avenue, Pittsburgh, PA 15213, USA; Joint CMU-Pitt PhD Program in Computational Biology, Pittsburgh, PA 15213, USA; Department of Epidemiology, University of Florida, 2004 Mowry Rd, Gainesville, FL 32610, USA

**Keywords:** mixed graphical models, survival analysis, machine learning

## Abstract

**Background:**

Many research domains are producing large, multi-scale, multi-modal datasets at growing rates with mixed variable types (continuous, discrete, censored). Identifying possible cause-effect associations in such datasets is essential for predicting outcomes and proposing possible interventions. Probabilistic graphical models (PGMs) have emerged as a robust, interpretable way to analyze such datasets, but current graph learning algorithms cannot incorporate time-to-event (censored) variables, which are important in many systems (e.g., patient survival). Instead, regression models are typically used for survival analysis of *single* censored variables, but these cannot assess cause-effect interactions.

**Results:**

Here, we present a new mathematical framework to incorporate *multiple censored variables* into mixed graphical models. A novel efficient algorithm, *CausalCoxMGM*, is implemented, which is extensively evaluated on synthetic and real-life high-dimensional biomedical datasets (cardiovascular disease, breast cancer). *CausalCoxMGM* was able to recover effectors of censored variables, supported by literature, and provided new mechanistic insights on the differences between ER+ and ER− breast cancers.

**Conclusions:**

*CausalCoxMGM* is a flexible computational framework for learning potential cause-effect relations from observational data of mixed data types, including multiple censored variables. The resulting graphs are interpretable and can be used to generate testable hypotheses or build efficient predictors of any outcome.

## Introduction

There has been an explosion of data collected in various fields in terms of both type and volume, including from biological and biomedical systems. Mining these complex, multi-modal, and multi-scale datasets for mechanistic insights is essential, especially when it involves time-to-event (censored) variables, like patient survival, time to cancer remission, or a machine’s time-to-failure. Machine learning has proven quite efficient for general analysis needs [1]. However, model interpretability remains a challenge. Though "black box" machine leanring methods often perform well in classification, they are not designed to identify the complex network of direct (cause-effect) interactions in a dataset, which is critical for gaining mechanistic insights and suggesting interventions to improve outcomes. Probabilistic graphical models (PGMs) have gained popularity in filling this gap. PGMs learn and represent conditional independence relations among all variables in a dataset, and prior work on directed [2, 3] and undirected [4, 5] PGMs has demonstrated their ability to improve inference through interpretable modeling of biological systems. Additionally, in the last decade, new algorithms have enabled PGMs to be learned on mixed datasets containing continuous and discrete variables [6–10].

However, in clinical settings and other fields, key features of interest are censored. Currently, graph-learning methods cannot incorporate censored variables. And most machine learning methods can only handle one censored variable in each dataset (frequently this is the target or outcome variable). Here we should note that a censored variable (partially observed) is different from the standard missing value problem, since censoring provides partial information (e.g., the patient did not die 16 months after the treatment).

We present the mathematical and algorithmic framework for learning mixed graphical models (MGMs) [6–8], incorporating (multiple) censored variables. We demonstrate its ability to recover adjacencies and edge orientations on synthetic datasets and to recover well-supported associations with censored outcomes in real-world cardiovascular disease datasets. Additionally, we demonstrate the ability of CausalCoxMGM to mine complex high-dimensional datasets for biological insights through the identification of clinical and gene expression signatures of breast cancer progression in estrogen receptor positive (ER+) and negative (ER-) subtypes. Despite having a common tissue of origin, ER+ and ER− breast cancers are distinct diseases [11] that undergo different pathways of progression [12, 13] associated with different biological processes [14].

## Methods

### Simulated data

We generated 20 Erdős–Rényi (ER; random) and 20 scale-free (SF) directed acyclic graphs (DAGs), each of 3 different node counts: 55, 110, and 550. Each graph included 5/11 continuous features, 5/11 discrete features, and 1/11 censored features, with an average node degree of 4. Additional ER and SF graphs with 110 nodes were generated to have average node degrees of 2 and 6 to study the effect of graph density.

To assess the effect of sample size on model learning, we simulated datasets of 100, 250, 500, 1,000, 5,000, and 10,000 samples for ER and SF graphs with 110 nodes and an average degree of 4. To assess the effect of censoring rate on graph recovery, each dataset was simulated under light censoring (30% censored; 70% observed) and heavy censoring (70% censored; 30% observed) conditions. For full simulation details, see Supplementary Methods.

### Biological data

Two cardiovascular disease datasets were used to construct causal models with censored outcomes. The first, *peakVO2* [6], includes 39 clinical, demographic, and exercise stress test features and all-cause mortality data from individuals with systolic heart failure. The second, *whas500*[8], contains 13 clinical and demographic features recorded from 500 patients hospitalized for acute myocardial infarction (AMI) in the Worcester Heart Attack Study [9]. Two censored outcomes were recorded: time-to-discharge and all-cause mortality, with in-hospital death treated as censoring in the time-to-discharge outcome.

Breast cancer gene expression and clinical data for the METABRIC study [15] were obtained from cBioPortal, while validation cohorts [16–24] were downloaded from GEO (see Supplementary Methods for accession numbers). The log_2_-transformed METABRIC microarray data were used as provided by cBioPortal. For each microarray validation cohort, raw CEL files were processed with *oligo* [25] to give log_2_-transformed data. For RNA-seq data, a variance-stabilizing transform was applied to raw counts with *DESeq2* [26]. ComBat, implemented in *sva* [27], was used to align validation cohorts with METABRIC gene expression data. We selected clinical features linked to breast cancer prognosis and subtype, including age at diagnosis, menopausal state, tumor size, lymph node status, histologic grade, histologic subtype, and receptor statuses (ER, PR, HER2) [11, 12]. Samples missing clinical data were excluded. After selecting highly variant genes and filtering to reduce multicollinearity (see Supplementary Methods), 437 gene expression features were included. The nonparanormal transform [13] was applied to continuous features to enable CoxMGM and CausalCoxMGM to capture rank-based associations.

The METABRIC dataset records breast cancer progression and mortality as censored time-to-event variables, including disease-specific survival (DSS), death by other causes (OD), distant relapse (DR), and locoregional relapse (LR). We also analyzed common combined metrics: overall survival (OS; DSS and OD), distant relapse-free survival (DRFS; DSS, OD, and DR), and disease-free survival (DFS; DSS, OD, DR, and LR). Due to differing progression pathways and baseline hazards for ER+ and ER− breast cancers, separate causal models were learned for each subtype. Further details on hyperparameter selection are provided in the Supplementary Methods.

### Statistical analysis

To evaluate causal graph recovery on simulated networks, we measure adjacency and orientation precision, recall, and *F*_1_ score [28] (Supplementary Methods; Table S1). Precision and recall metrics for CoxMGM and CausalCoxMGM are summarized over a range of hyperparameters by the area under the precision–recall curve (AUPRC), while individual model comparisons use *F*_1_ scores. Overall graph recovery is assessed with the structural Hamming distance (SHD) from the Markov equivalence class (MEC) of the true causal graph. The MEC is a partially directed graph that representing DAGs that yield the same conditional independence relationships (Supplementary Methods). The *F*_1_ score is also used to assess feature selection in CausalCoxMGM and LASSO Cox regression.

Predictive models constructed with CausalCoxMGM are Cox regression models regressed on the Markov blanket (MB) of each outcome, where the MB is the set of variables that renders the outcome independent of all other variables when conditioned upon. Predictors of composite outcomes in METABRIC (e.g., DRFS) are constructed with a multistate model (Supplementary Fig. S1) implemented with *mstate* [29]. Baseline models using LASSO Cox regression and random survival forests (RSF) were learned with *glmnet* [30] and *randomForestSRC* [31], respectively, with composite outcomes predicted directly. Predictive accuracy for censored variables is measured with Harrell’s concordance [32]. Internal validation uses 10-fold cross-validation, while external validation combines accuracy in individual datasets into a summary statistic for the Meta Cohort using *survcomp* [33].

## Results

### Overview of the method

**Figure 1:**
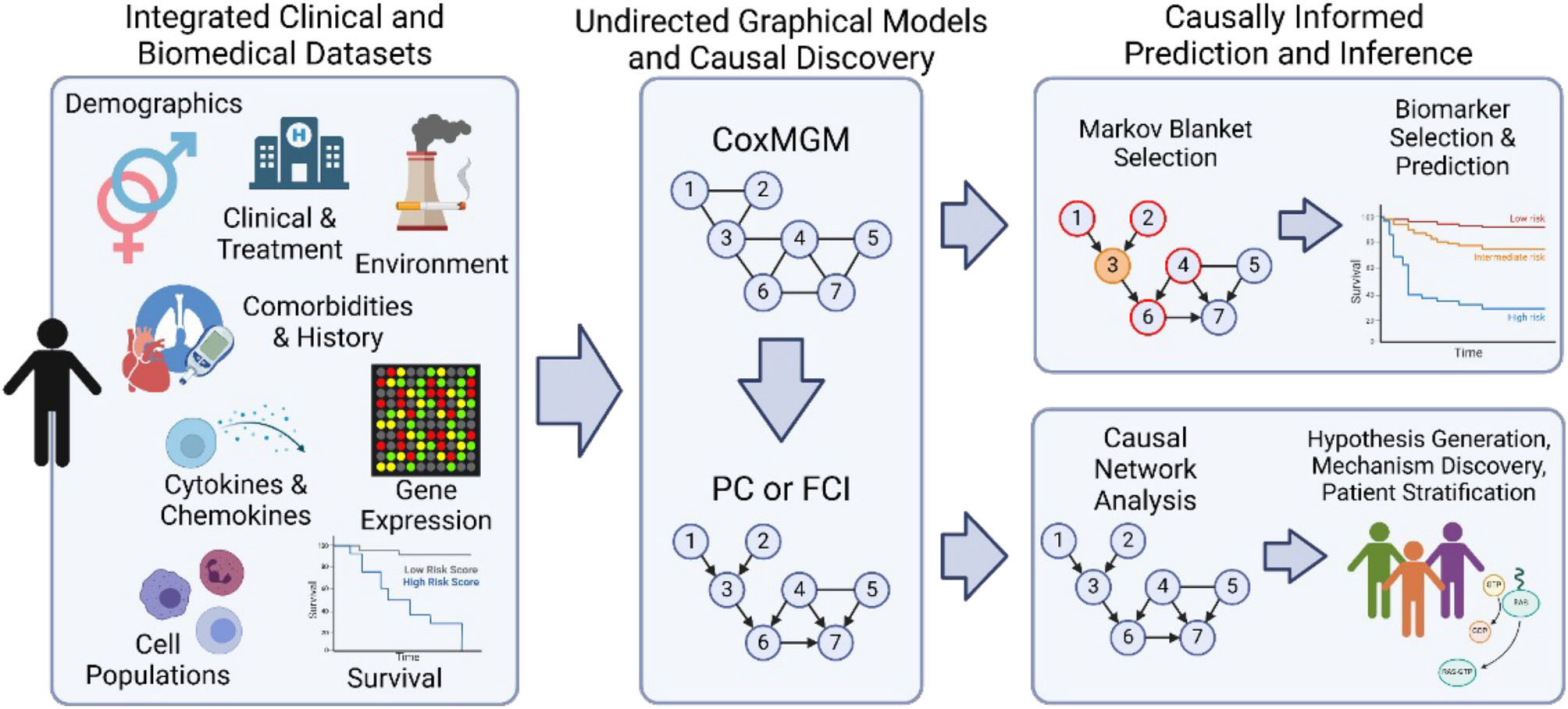
Schematic illustrating the CausalCoxMGM method. CausalCoxMGM takes heterogeneous clinical and biomedical data, including continuous, discrete, and censored variables, as input. An interpretable causal PGM is then inferred in a 2-step process: (1) an undirected graphical model is learned with the proposed CoxMGM and (2) constraint-based causal discovery algorithms such as PC or FCI are applied to the undirected skeleton. The resulting causal graphical model can be used to construct parsimonious and robust predictors, and its interpretability aids hypothesis generation, patient stratification, and mechanism discovery.

CausalCoxMGM (Fig. 1) provides a framework for learning interpretable causal PGM over heterogeneous datasets containing continuous, discrete, and censored variables. This approach enables the integration of biomedical data from varying modalities (e.g., transcriptomics, metabolomics) with clinical and demographic data and censored outcomes. CausalCoxMGM learns the graphical model through a 2-step process: (1) learning an initial estimate of the adjacencies with the undirected graphical model CoxMGM and (2) pruning spurious adjacencies and orienting edges with constraint-based causal discovery algorithms such as PC or FCI. The resulting causal graphical model not only enables the construction of parsimonious and robust predictors of censored outcomes, but also provides insights for hypothesis generation, risk stratification, and understanding mechanisms driving patient outcomes.

### Cox mixed graphical models

The CoxMGM utilizes the Cox proportional hazards model (Supplementary Methods) to expand the MGM [6] (Supplementary Methods) framework to incorporate censored variables along with continuous and discrete data types. Here, we denote continuous and discrete variables with *x* and *y*, respectively, while censored variables are represented as pairs $\{ {t,\ {\mathrm{\delta }}} \}$, where *t* is the event time or last follow-up, and ${\mathrm{\delta }}$ is the event indicator. This model allows flexible penalization across edge types by assigning different edge potentials: ${\mathrm{\gamma }}$ for continuous-censored edges and ${\mathrm{\psi }}$ for discrete-censored edges.

Using a second-order approximation of the Cox proportional hazards partial log-likelihood, CoxMGM defines a joint distribution over *x, y*, and $\{ {t,\ {\mathrm{\delta }}} \}$ as


\begin{eqnarray*}
&& P(x,y,t,\delta;\Theta) \propto \exp \left(\sum \limits_{s = 1}^p \sum \limits_{t = 1}^p - \frac{1}{2}\beta_{st}{x_s}{x_t} + \sum \limits_{s = 1}^p \alpha_s{x_s} + \sum \limits_{s = 1}^p \sum \limits_{j = 1}^q\right.\\&& \rho_{sj}\left(y_j\right){x_s} + \sum \limits_{j = 1}^q \sum \limits_{k = 1}^q {\phi_{jk}}\left(y_j,y_k\right) + \sum \limits_{s = 1}^p \sum \limits_{m = 1}^r \gamma_{sm}{x_s}{W_m}{z_m}\\&&\, \left. + \sum \limits_{j = 1}^q \sum \limits_{m = 1}^r \psi_{jm}\left(y_j\right){W_m}{z_m}\right),
\end{eqnarray*}

where $\widehat {{{{\mathrm{\eta }}}_m}} = \widehat {{{{\mathrm{\gamma }}}_m}}x + \widehat {{{{\mathrm{\psi }}}_m}}( y )$, ${{z}_m} = \widehat {{{{\mathrm{\eta }}}_m}} - l^{\prime\prime}{{( {\widehat {{{{\mathrm{\eta }}}_m}}} )}^{ - 1}}l^{\prime}( {\widehat {{{{\mathrm{\eta }}}_m}}} ),$ and ${{W}_m}$ is a diagonal weight matrix such that ${\mathrm{diag}}( {{{W}_m}} ) = {\mathrm{diag}}( {l^{\prime\prime}( {\widehat {{{{\mathrm{\eta }}}_m}}} )} )$. Efron’s correction for tied times [34] is used when multiple samples experience events or censoring at the same time.

Node-wise conditional distributions under CoxMGM are modeled by Gaussian linear regressions for continuous variables, multinomial logistic regressions for discrete variables, and Cox regressions for censored variables. This model is fit by minimizing the negative log-pseudolikelihood:


\begin{eqnarray*}
&& {\mathrm{\tilde{l}}}\left( {{\mathrm{\Theta |}}x,y,t,{\mathrm{\delta }}} \right) = - \mathop \sum \limits_{s = 1}^p \log p\left( {{{x}_s}{\mathrm{|}}{{x}_{ \setminus s}},y,t,{\mathrm{\delta }};{\mathrm{\Theta }}} \right)\\&&\, - \mathop \sum \limits_{j = 1}^q \log p\left( {{{y}_j}{\mathrm{|}}x,{{y}_{ \setminus j}},t,{\mathrm{\delta }};{\mathrm{\Theta }}} \right) - \mathop \sum \limits_{m = 1}^r \log p\left( {{{T}_m},{{{\mathrm{\delta }}}_m}{\mathrm{|}}x,y;{\mathrm{\Theta }}} \right).
\end{eqnarray*}

To flexibly encourage sparsity, CoxMGM applies separate penalties for each edge type: ${{{\mathrm{\lambda }}}_{cc}}$ for continuous-continuous edges, ${{{\mathrm{\lambda }}}_{cd}}$ for continuous-discrete edges, ${{{\mathrm{\lambda }}}_{dd}}$ for discrete-discrete edges, ${{{\mathrm{\lambda }}}_{sc}}$ for continuous-censored edges, and ${{{\mathrm{\lambda }}}_{sd}}$ for discrete-censored edges. The penalized pseudolikelihood is


\begin{eqnarray*}
&& \mathop {\min }\limits_{\mathrm{\lambda }} \widetilde {{{l}_\lambda }}\left( {\mathrm{\Theta }} \right) = \tilde{l}\left( {\mathrm{\Theta }} \right) + {{{\mathrm{\lambda }}}_{cc}}\mathop \sum \limits_{s = 1}^p \mathop \sum \limits_{t = 1}^{s - 1} \left| {{{{\mathrm{\beta }}}_{st}}} \right| + {{{\mathrm{\lambda }}}_{cd}}\mathop \sum \limits_{s = 1}^p \mathop \sum \limits_{j = 1}^q \parallel {{\rho }_{sj}}{{\parallel }_2}\\&&\, + {{{\mathrm{\lambda }}}_{dd}}\mathop \sum \limits_{j = 1}^q \mathop \sum \limits_{k = 1}^{j - 1} \parallel {\psi_jk}{{\parallel }_F} + {{{\mathrm{\lambda }}}_{sc}}\mathop \sum \limits_{s = 1}^p \mathop \sum \limits_{m = 1}^r \left| {{{{\mathrm{\gamma }}}_{sm}}} \right| + {{{\mathrm{\lambda }}}_{sd}}\mathop \sum \limits_{j = 1}^q \mathop \sum \limits_{m = 1}^r \parallel {{\psi }_{jm}}{{\parallel }_2}.
\end{eqnarray*}

Each penalty value is selected using the Stable Edge-specific Penalty Selection (StEPS) algorithm [7], optimizing regularization to maintain stability across subsamples. Nonzero regression coefficients indicate edges in the learned graph structure, enabling CoxMGM to model relationships among continuous, discrete, and censored variables.

### Independence test for censored variables in mixed datasets

For constraint-based causal discovery on datasets containing censored, continuous, and discrete variables, we need an independence test that accommodates censoring. Expanding on an independence test for mixed datasets (Supplementary Methods), our method must test: (1) whether a censored variable *X* is independent of another variable *Y* given conditioning set *S*, and (2) whether *X* is independent of *Y* given a conditioning set *S* that includes censored variables.

The first task can be accomplished using Cox regression as follows:

- When *Y* is continuous, we use Cox regression to model *X* with respect to *Y* and *S*, then perform a *t*-test on *Y*’s coefficient to calculate its *P*-value.
- When *Y* is discrete, we compare 2 Cox models of *X*: one conditioned on *S* alone (null model) and the other on *Y* and *S*. A likelihood ratio test between these 2 models yields the *P*-value.

If the *P*-value is below the threshold ${\mathrm{\alpha }}$ we reject the null hypothesis of conditional independence.

The second task, involving conditioning on censored variables in *S*, is more complex due to *expansion bias* [35]. Expansion bias occurs because censoring constrains reported values to be less than the true event time, skewing regression coefficients when both uncensored and censored values are included in the analysis. A common but limited workaround is to perform the regression using only samples where all censored variables in *S* are observed. While unbiased under uninformative censoring, this approach reduces test power as censorship increases or as more censored variables are included in *S*.

We propose an alternative approach to conditioning on censored variables that leverages the CoxMGM formulation. Instead of directly using censored values, we condition on censored covariates through the term $Wz$ derived from the second-order approximation of the Cox model. Specifically, for each censored covariate, we use its null model to compute $Wz$, where $Wz$ reduces to the Martingale residuals of the null model. These residuals measure the difference between the number of observed and expected events up to the observation time [36], approximating the functional associations of censored covariates in conditional independence tests. This approach has 2 key benefits: (1) we retain all samples in conditional independence tests and (2) Martingale residuals have been shown to effectively approximate functional relationship between covariates and censored variables in the Cox model [36], making them ideal for representing censored covariates in our test.

### Simulated network recovery

To evaluate the performance and limitations of our causal discovery algorithms, we generated synthetic Erdős–Rényi (ER) and SF DAGs across a range of different graph sizes, graph degrees, sample sizes, and censorship conditions. As graph recovery depends on hyperparameter selection, we assess the performance of both CoxMGM (undirected graph) and CausalCoxMGM (directed graph) across a range of hyperparameters, summarizing results using AUPRC. Additionally, we demonstrate CausalCoxMGM’s effectiveness in feature selection compared to LASSO Cox regression.

#### Undirected graph recovery with CoxMGM

**Figure 2:**
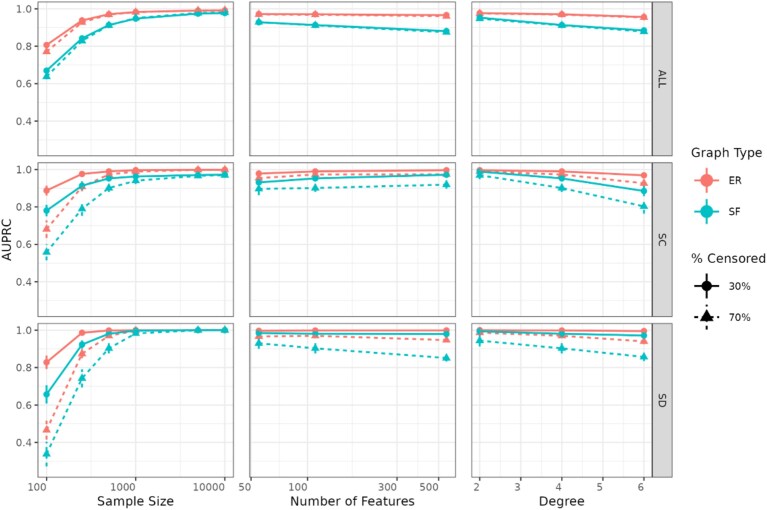
Results for undirected graph adjacency recovery in simulated datasets. AUPRC values are presented across all edges (ALL), continuous-censored edges (SC), and discrete-censored edges (SD). When sample size is varied, the number of features is 110 and degree is 4. When the number of features is varied, the sample size is 500, and the degree is 4. When the degree is varied, the sample size is 500, and the number of features is 110. Error bars denote 95% confidence intervals.

Figure 2 illustrates CoxMGM’s adjacency recovery across a range of sample sizes, graph sizes, and graph degrees, showing that AUPRC approaches 1 with increasing sample sizes for all edges, including those with censored variables (SC, SD), confirming the algorithm’s asymptotic correctness. Notably, at a fixed sample size of 500, increasing the number of nodes or graph degree results harms graph recovery, especially in SF graphs. As expected, SF graphs are more difficult to learn than Erdős–Rényi (ER) graphs due to the limited number of high-degree nodes, although this difference diminishes as sample size increases.

A key aspect for assessing CoxMGM’s performance is the effect of censoring rates on the recovery of the causal skeleton. High censorship (i.e., few observed events) hampers graph recovery across all edge types (ALL) and especially for edges involving censored variables (SC, SD). This effect is most pronounced at low sample sizes, large graphs, high graph degrees, and for edges connecting censored and discrete variables (SD). As expected, the power to recover edges involving censored variables is directly related to the number of observed events, though this limitation decreases as sample size increases.

Finally, stability-based hyperparameter selection strategies for undirected graphical models (StARS, StEPS) perform well compared to an oracle and outperform the Bayesian Information Criterion (BIC) score at larger sample sizes (Supplementary Fig. S2A). At lower sample sizes, the models selected by BIC, StARS, and StEPS have similar ${{F}_1}$ scores across all edge types. However, as sample size increases, StARS and StEPS significantly outperform BIC. Furthermore, in large and high-degree graphs, StEPS significantly outperforms StARS, particularly in edges between censored and continuous variables (Supplementary Fig. S2B, C).

#### Causal skeleton recovery with CausalCoxMGM

**Figure 3:**
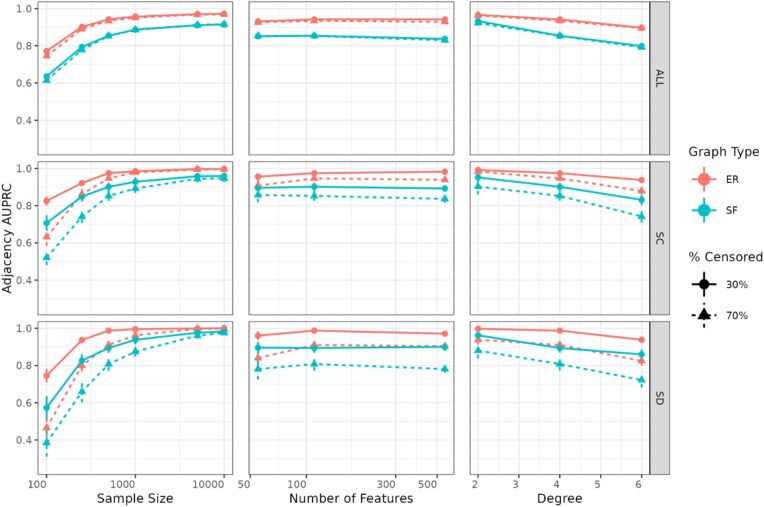
Results for directed graph adjacency recovery in simulated datasets. AUPRC values are presented across all edges (ALL), continuous-censored edges (SC), and discrete-censored edges (SD). When sample size is varied, the number of features is 110 and degree is 4. When the number of features is varied, the sample size is 500, and the degree is 4. When the degree is varied, the sample size is 500, and the number of features is 110. Error bars denote 95% confidence intervals.

We assess CausalCoxMGM’s ability to recover the adjacencies in the true causal graph after applying MPC-Stable (Majority PC) to the initial skeleton estimated by CoxMGM selected via StEPS. Figure 3 shows the AUPRC for causal skeleton recovery in the same simulated networks as Fig. 2, allowing us to evaluate performance across various ${\mathrm{\alpha }}$ values, sample sizes, graph sizes, graph degrees, and censorship rates. The results mirror the trends observed with CoxMGM, with AUPRC asymptotically approaching 1 as sample size increases. As expected for causal discovery algorithms, low sample sizes (<250 samples) significantly decrease the adjacency AUPRC. However, we note that even at low sample sizes (*n* = 100) it is possible to recover adjacencies with high precision (i.e., with a low false discovery rate; Supplementary Fig. S3).

The effect of high graph degree and SF graph architectures is more pronounced in CausalCoxMGM, highlighting a known limitation of constraint-based causal discovery methods. Despite this, precision remains relatively high across all conditions, while the recall increases substantially as sample size increases (Supplementary Fig. S3). This is especially pronounced in SF networks and in edges involving censored variables. High censoring rates significantly reduce recall in these edges, but this effect diminishes as sample size increases.

#### Causal orientation recovery with CausalCoxMGM

**Figure 4:**
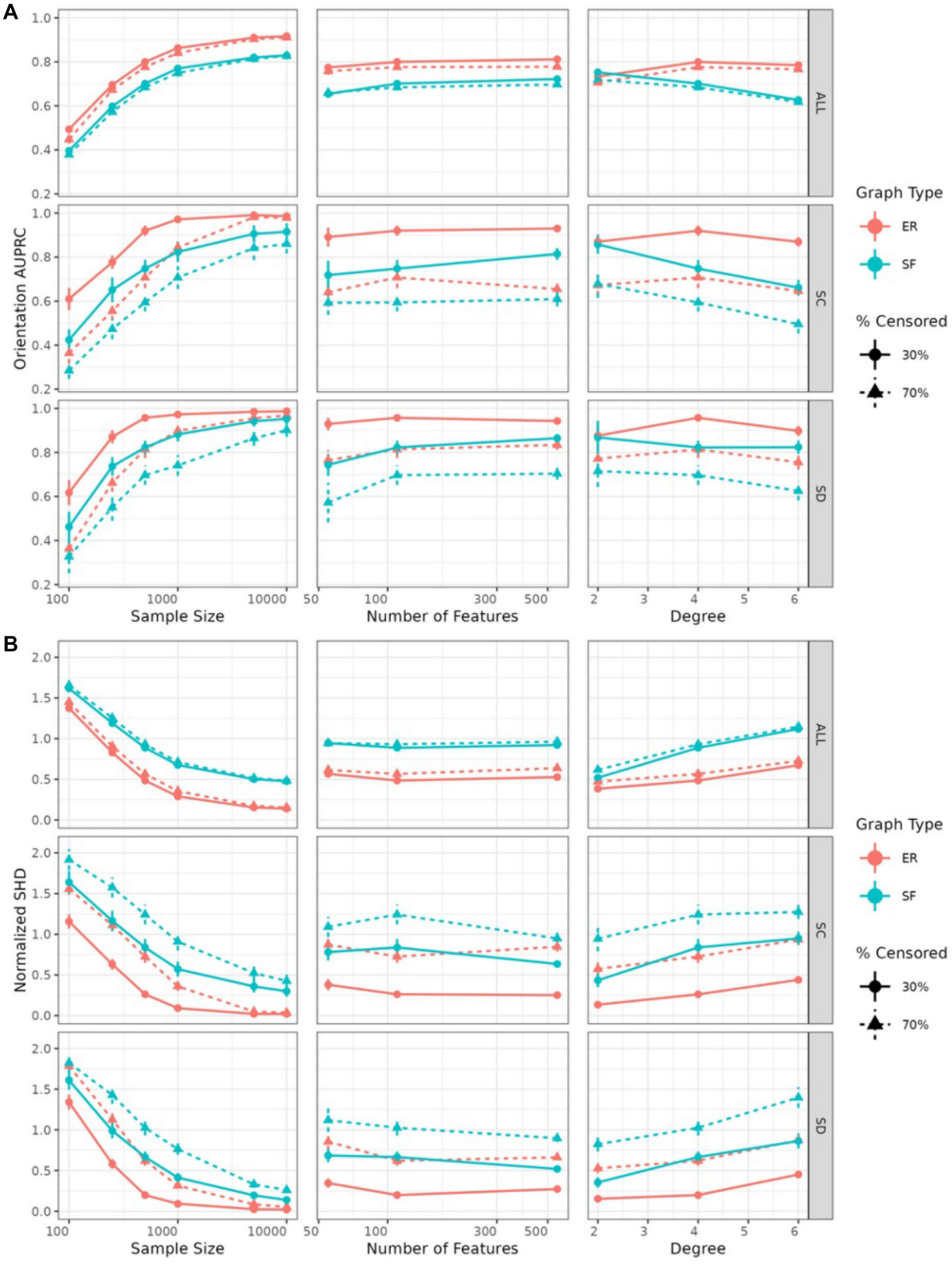
Results for CausalCoxMGM’s causal structure recovery in simulated datasets. Values are presented across all edges (ALL), continuous-censored edges (SC), and discrete-censored edges (SD). (A) Orientation AUPRC across simulation conditions. (B) Normalized SHD across simulation conditions. When sample size is varied, the number of features is 110 and degree is 4. When the number of features is varied, the sample size is 500, and the degree is 4. When the degree is varied, the sample size is 500, and the number of features is 110. Error bars denote 95% confidence intervals.

A key benefit of the CausalCoxMGM algorithm is its ability to infer directionality in the graphical model. Here, we evaluate its performance in recovering the true orientations by assessing the precision and recall of directed edge orientations in the estimated graph compared to the true causal DAG. Figure 4A illustrates orientation AUPRC across sample sizes, graph sizes, graph degrees, and censorship rates for the same ER and SF networks as Fig. 2.

As sample size increases, recovery of causal orientations again improves across all conditions. However, this occurs at a slower rate than adjacency recovery, as orienting edges is more challenging. This suggests that at lower sample sizes (*n* < 500), adjacency recovery is more reliable than orientation recovery. The orientation AUPRC also levels off asymptotically at a value below 1, as constraint-based methods like CausalCoxMGM cannot orient all edges from observational data alone and instead return the MEC. High censoring rates hinder the recovery of orientations in edges involving censored variables to a greater degree than adjacencies, although the AUPRC still increases asymptotically. Notably, high censoring rates have a greater impact on overall orientation recovery than overall adjacency recovery.

#### Combined measure of causal graph discovery

SHD is a combined metric of causal graph recovery that integrates adjacency and orientation information. As a distance metric, it enables the principled selection of the best model without the need to balance adjacency and orientation accuracy. Figure 4B displays the normalized SHD for graphs learned with α = 0.05 across various sample sizes, graph sizes, graph degrees, and censorship rates for the same networks as Fig. 2.

Increasing sample size results in lower normalized SHD across network types, with SHD values for SF graphs consistently higher than ER graphs, reflecting their challenging to recover structure. Additionally, higher censorship rates lead to poorer structure recovery for edges involving censored variables. The normalized SHD remains relatively stable across graph sizes at a fixed sample size, while increasing graph degree hampers graph recovery.

#### Feature selection performance

CausalCoxMGM not only learns the structure of causal models around censored variables but also serves as a powerful tool for feature selection for downstream prediction tasks. In our simulated networks, CausalCoxMGM more effectively selects parsimonious feature subsets compared to LASSO Cox regression. Specifically, at sample sizes greater than 500, the MB and direct neighbors of censored variables in our causal graphical models recovered the true associations with covariates more accurately, as measured by the *F*_1_ score (Supplementary Fig. S4A).

The LASSO Cox regression model selected at the minimum deviance from 10-fold cross-validation consistently underperformed, while the model selected by the one standard error (1SE) rule performed similarly or better than CausalCoxMGM at low sample sizes. Notably, even at low sample sizes, CausalCoxMGM achieved significantly higher precision than LASSO Cox regression using the 1SE rule (Supplementary Fig. S4B).

### Causal discovery in biomedical datasets with censored outcomes

Going beyond simulated data, we applied CausalCoxMGM to real-life biomedical datasets with censored variables, specifically focusing on low-dimensional clinical datasets related to cardiovascular disease. In this context, where associations between clinical features and outcomes are more easily verified against existing literature, we demonstrate that CausalCoxMGM effectively learns causal interactions involving censored variables.

#### All-cause mortality in systolic heart failure

We applied CausalCoxMGM to identify features affecting all-cause mortality in individuals with systolic heart failure from a dataset containing clinical, demographic, and cardiopulmonary stress testing features [37]. The resulting causal graphical model (Supplementary Fig. S5) identified 7 features directly linked to all-cause mortality. Notably, decreased peak oxygen consumption (Peak VO2) and treadmill exercise duration were linked to increased mortality risk. Additional factors linked to increased mortality risk include lower left ventricular ejection fraction (LVEF), male gender, and elevated blood urea nitrogen (BUN), the latter serving as an indicator of renal function. Furthermore, treatment with beta-blockers reduced mortality risk, while treatment with digoxin increased it.

#### Mortality and time-to-discharge after hospitalization for AMI

We also applied CausalCoxMGM to a small cohort [38] from the Worcester Heart Attack Study [39], focusing on outcomes for patients hospitalized for AMI. This study includes 2 censored variables: time-to-discharge (where discharge is the event and deaths before discharge are censored) and all-cause mortality after admission. The causal graphical model learned by CausalCoxMGM (Supplementary Fig. S6) captured expected associations with reasonable causal orientations and directions (positive/negative) of association. Higher BMI was associated with decreased mortality risk after AMI, while low diastolic blood pressure was linked to increased risk. Additionally, elevated heart rate and higher age at admission, both established independent risk factors for in-hospital mortality after an AMI [40], were linked to elevated mortality risk. Finally, 2 complications, congestive heart failure and cardiogenic shock, were associated with increased mortality risk post-AMI. Utilizing our time-to-discharge data, we found that these 2 complications, along with atrial fibrillation, contributed to prolonged hospital stays due to the need for additional care and monitoring.

### Causal analysis of subtype-specific breast cancer progression

**Figure 5:**
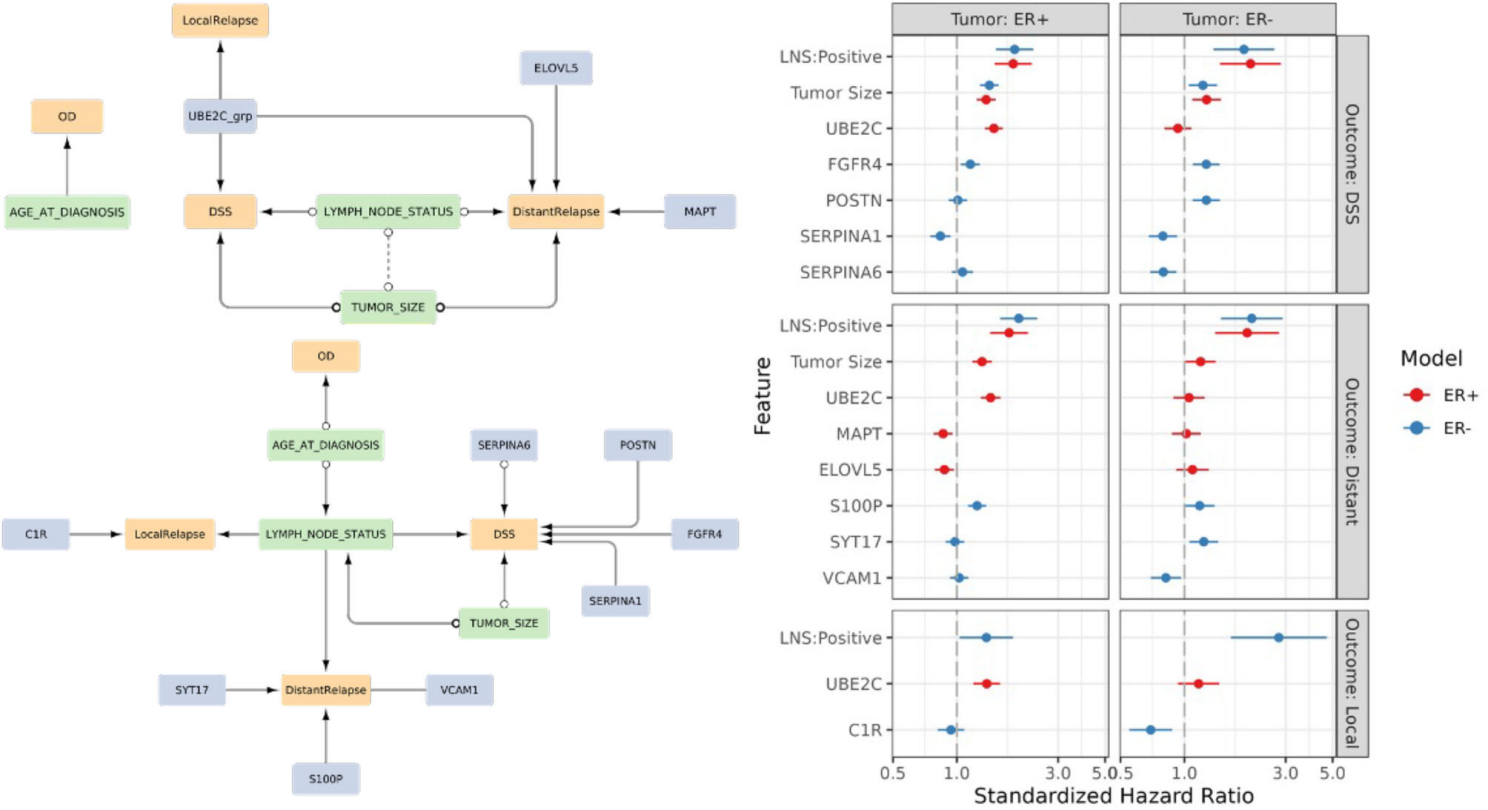
Subtype-specific causal models reveal distinct gene expression drivers of breast cancer progression. (A) Subgraph of the CausalCoxMGM model for estrogen receptor positive (ER+) tumors containing censored outcomes and their MB. (B) Subgraph of the CausalCoxMGM model for estrogen receptor negative (ER−) tumors containing censored outcomes and their MB. Orange nodes are censored outcomes, green nodes are clinical features, and blue nodes are gene expression features. A directed edge X → Y indicates X is a cause of Y, a bidirected edge X ↔ Y indicates X and Y have a shared latent confounder, a partially oriented edge X o→ Y indicates Y is not a cause of X but it is unclear whether X causes Y or they share a latent confounder, and an unoriented edge X o-o Y indicates that causal orientation cannot be inferred for that edge. (C) Forest plots depicting the standardized hazard ratios (and 95% confidence intervals) from a multivariate Cox regression model for the MB of each mode of breast cancer progression. Standardized hazard ratios for features selected by both the ER+ (red) and ER− (blue) models are estimated in ER+ (right) and ER− (left) tumors. DSS: disease-specific survival; OD: death by other causes; DR: distant relapse; LR: locoregional relapse.

We applied CausalCoxMGM to the METABRIC breast cancer dataset to identify potential causal drivers of DSS, DR, LR, and death by OD amongst gene expression features and clinical covariates. Due to distinct mechanisms of development and progression [12] and dynamics of relapse and survival [13] in ER+ and ER− breast cancers, we learned and compared 2 separate models (Fig. 5A). Notably, 2 clinical features directly describing disease progression, tumor size, and lymph node status, are linked to DSS and/or relapse in both subtype models and are established prognostic indicators of survival in breast cancer [41]. As expected, age at diagnosis is the only feature linked to death by OD.

In contrast, gene expression features linked to breast cancer progression differ between ER+ and ER− subtypes (Fig. 5B). We assessed the association of these features with DSS, DR, and LR by learning multivariate Cox proportional hazards models based on each feature’s MB in the ER+ and ER− models. In ER+ tumors, high UBE2C expression was linked to increased disease-specific mortality and relapse, and low expression of MAPT and ELOVL5 is linked to increased DR. In ER− tumors, high expression of FGFR4 and POSTN and low expression of SERPINA1 and SERPINA6 were linked to increased disease-specific mortality. Additionally, elevated S100P and SYT17 and decreased VCAM1 expression are linked to DR, and low C1R expression is associated with LR.

We also tested whether gene expression features identified in the causal model of one subtype were significantly associated with progression in the other subtype. For DSS, DR, and LR, the ER+ model genes were not significantly associated with progression in ER− tumors. Similarly, most ER− model genes, except for SERPINA1, S100P, and FGFR4, were not significantly associated with progression in ER+ tumors. These results underscore the distinct molecular drivers of progression in ER+ and ER− breast cancer subtypes.

#### Subtype-specific predictive model of breast cancer progression

Using the subset of features identified by our causal graphs, we constructed subtype-specific predictors of DSS, OD, DR, and LR. Composite outcomes such as OS, DRFS, and DFS were modeled using a multi-state Cox model based on these individual outcomes. Internal validation was performed with 10-fold cross-validation, and external validation was conducted through meta-analysis of predictive accuracy in 8 external breast cancer cohorts with at least one of DSS, OS, DRFS, and DFS. We compared our models to baseline LASSO Cox regression and RSF models.

**Figure 6:**
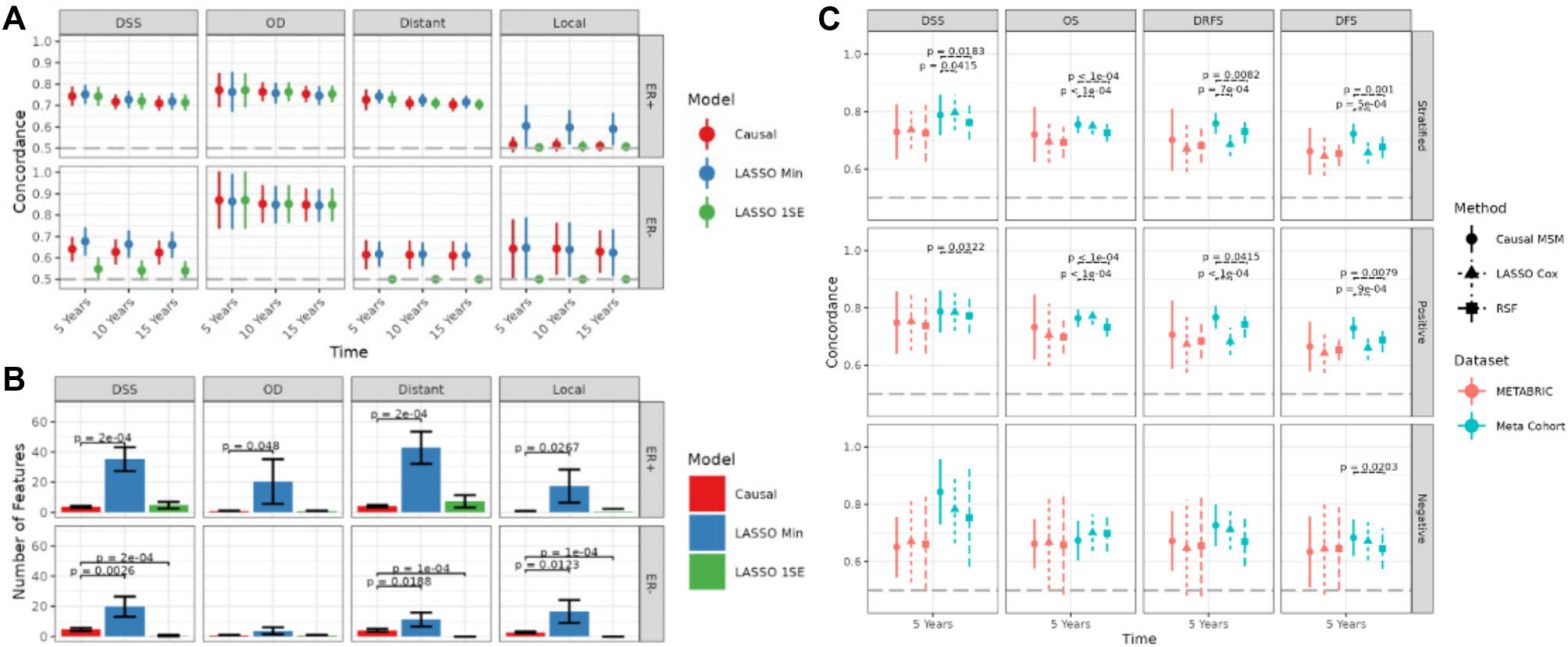
Predictive accuracy and feature selection parsimony in internal and external validation for CausalCoxMGM compared to LASSO Cox regression selected at the minimum deviance (Min) and 1SE rule and RSF baselines. (A) Harrell’s concordance statistic at 5, 10, and 15 years from baseline for each censored outcome estimated through 10-fold cross-validation. (B) Number of features selected by each predictive model for each outcome estimated through 10-fold cross-validation. (C) Harrell’s concordance statistic at 5 years for common measures of breast cancer outcomes in METABRIC (estimated through 10-fold cross-validation) and an external Meta Cohort. Dashed lines at 0.5 denote expected concordance for random predictions. FDR-adjusted *P*-values are given for all significant differences between CausalCoxMGM and baselines (FDR *P* < 0.05). DSS: disease-specific survival; OD: death by other causes; DR: distant relapse; LR: locoregional relapse; DRFS: distant relapse-free survival; DFS: disease-free survival.

In 10-fold cross-validation, CausalCoxMGM models were able to successfully predict most censored outcomes in breast cancer except for LR. In contrast, LASSO Cox regression struggled to learn parsimonious predictors. LASSO Cox regression models selected with minimum cross-validation deviance (Min) were able to successfully predict censored outcomes other than LR but required significantly more features (Fig. 6A, B). The LASSO Cox regression models selected with the 1SE rule selected a similar number of features as CausalCoxMGM but exhibited lower predictive accuracy, especially in ER− tumors, where it failed to predict DSS, DR, and LR.

To further validate our findings, we applied these models to an external composite validation cohort of 8 breast cancer datasets that recorded various measures of breast cancer progression and mortality. Apart from DSS, these cohorts recorded composite measures of breast cancer progression (OS, DRFS, DFS) instead of distinct outcomes. We predicted these outcomes using the multi-state model described above, calculating probabilities for transitions to corresponding states (e.g., DSS, OD, and DR for DRFS). LASSO Cox regression and RSF models, trained directly on these composite outcomes, served as baselines.

Figure 6C shows that CausalCoxMGM significantly predicts all 4 outcomes in the external meta cohort, both overall and in ER+ and ER− tumors individually. CausalCoxMGM performs comparably to LASSO Cox regression and RSF on simpler outcomes (DSS, OS), but outperforms them on more complex outcomes (DRFS, DFS). Additionally, the CausalCoxMGM multi-state model utilizes a smaller, more interpretable set of features than those identified by LASSO Cox regression.

An analysis of the calibration, measured by the 5-year Brier score (Supplementary Fig. S7A, B), showed that CausalCoxMGM and the baseline models were similarly calibrated in the METABRIC dataset. In contrast, none of the models achieved consistent calibration in the external validation cohorts, resulting in wide bootstrapped confidence intervals. In conjunction with our model’s strong discrimination accuracy, this lack of calibration likely reflects shifts in baseline hazards between the older METABRIC dataset, with tumors collected prior to the development of HER2-targeted therapies, and the more contemporary external validation cohorts. In the SCAN-B cohort (GSE96058; Supplementary Fig. S7C), for example, our CausalCoxMGM model consistently overestimated mortality, especially in ER− tumors.

#### Tumor microenvironment and mortality in ER− tumors

To investigate possible mechanistic roles of CausalCoxMGM prognostic genes, we performed bulk cell type deconvolution [42] of the METABRIC dataset using a single-cell breast cancer atlas as a reference [43]. Through this deconvolution, we analyzed cell-type-specific expression of the identified prognostic genes (Supplementary Fig. S8). As expected, across ER+ and ER− tumors, genes associated with breast cancer progression are predominantly expressed by cancer epithelial cells, including UBE2C, MAPT, and ELOVL5 in ER+ tumors and FGFR4, S100P, SERPINA6, and SYT17 in ER− tumors. However, in ER− tumors, certain genes associated with DSS, DR, and LR (POSTN, VCAM1, and C1R) were primarily expressed in cancer-associated fibroblasts (CAFs). This observation aligns with POSTN’s known roles in promoting metastasis and maintaining stem cell-like properties in breast cancer cells [44, 45]. Additionally, SERPINA1, linked to DSS in ER− tumors, was primarily expressed by myeloid cells, suggesting immune involvement.

## Discussion

We introduce CausalCoxMGM, a method for causal discovery in datasets with continuous, discrete, and censored variables. CausalCoxMGM combines (1) an undirected CoxMGM that models linear interactions across variable types and (2) a regression-based conditional independence test enabling constraint-based causal discovery in heterogeneous datasets with censored outcomes. These advancements make CausalCoxMGM a unique tool for causal discovery in datasets with censored outcomes, allowing robust identification of causal relationships and providing novel insights into subtype-specific breast cancer progression.

CausalCoxMGM recovered known associations with mortality in systolic heart failure and AMI patients. In systolic heart failure, it linked mortality to known predictors such as peak VO2 [46], treadmill exercise duration [37, 47], LVEF [47, 48], gender [49], and BUN [47, 50], supporting previous findings. Additionally, beta-blockers, recommended as a first-line therapy [51, 52], were associated with reduced mortality risk, while digoxin was linked to increased mortality, aligning with a recent retrospective analysis that found similar risks for patients without atrial fibrillation [53]. In individuals hospitalized with AMI, CausalCoxMGM identified known links with mortality and time-to-discharge, including a well-documented “obesity paradox” where high BMI is linked to lower mortality [54]. It also identified a link between diastolic blood pressure and mortality risk after AMI, despite its U-shaped relationship with mortality [55] violating linearity assumptions in our model. Three complications of AMI (congestive heart failure, cardiogenic shock, and atrial fibrillation) are also linked to mortality, time-to-discharge, or both with expected positive/negative associations.

In our subtype-specific analysis of breast cancer, CausalCoxMGM linked clinical features of well-established significance (lymph node status and tumor size) [41] and eleven genes with breast cancer progression. In ER+ tumors, CausalCoxMGM identified UBE2C overexpression as a driver of breast cancer progression, a finding supported by mechanistic work that showed UBE2C promotes tumor growth in ER+/HER2− tumors in both an estrogen-dependent and independent manner [56]. UBE2C is a downstream target of ERα-signaling that promotes cell cycle progression and proliferation, and constitutive overexpression promotes cell proliferation even in breast cancer cell lines treated with tamoxifen, an inhibitor of ERα-signaling. Combined with our model of breast cancer progression in ER+ tumors, these results suggest that drugs targeting UBE2C, used alone and in combination with hormone therapies such as tamoxifen, could result in better outcomes for patients with ER+ breast cancer.

In ER− breast cancer, overexpression of FGFR4 is linked to poor DSS, consistent with experimental work in triple-negative (ER−/PR−/HER2−) breast cancer cell lines showing FGFR4 promotes cell survival through PI3K/AKT activation and FGFR4 knockdown induces cell death [57]. Additionally, they provide evidence for co-expression of FGFR4 and FGF19 and show that inhibition of FGF19 also induces cell death in a dose-dependent manner, suggesting that FGFR4-FGF19 autocrine signaling promotes breast cancer cell survival in some ER− tumors [57]. Disruption of this signaling pathway could lead to treatments for triple-negative breast cancer, which is particularly aggressive and difficult to treat [58].

Additional genes were linked with DR in breast cancer. In ER+ tumors, low MAPT and ELOVL5 expression has been linked to breast cancer progression [59, 60], with experiments in mice showing that ELOVL5 knockdown promotes metastasis [60]. In triple-negative tumors, S100P overexpression has been linked to worse outcomes [61, 62], with S100P knockdown inhibiting trans-endothelial migration *in vitro* [61]. Notably, low VCAM1 expression was associated with increased risk of DR in ER− tumors, contradicting prior experimental work showing VCAM1 promotes lung and bone metastasis [63–65]. However, the association of low VCAM1 with DR is strongly significant in ER− tumors in METABRIC, and the trend is maintained in DRFS in ER− tumors in our external validation cohorts (Supplementary Fig. S9). Our cell-type-specific analysis showed VCAM1 is most highly expressed in CAFs, suggesting VCAM1’s role in the tumor microenvironment may differ from its role in metastatic cancer cells, warranting further investigation.

CausalCoxMGM’s ability to identify robust, interpretable associations between clinical and gene expression features and distinct modes of breast cancer progression in both ER+ and ER− tumors enabled the construction of a multi-state model of complex outcomes that generalized well to external cohorts. This multi-state model particularly excelled in predicting composite outcomes (DRFS, DFS), outperforming baselines trained directly on these outcomes. CausalCoxMGM’s interpretability also facilitated identification of subtype-specific genes linked to progression with potential as prognostic markers or therapeutic targets.

Despite these strengths, CausalCoxMGM has several limitations. Constraint-based causal discovery cannot definitively establish causality in observational data due to assumptions of acyclic cause-effect relationships, Markov faithfulness, and asymptotically large sample sizes. CausalCoxMGM further assumes linear interactions, though the nonparanormal transform can relax this to additive monotonic interactions. Simulations showed that certain causal structure characteristics (e.g., high degree, SF networks) can hinder causal structure recovery. Low sample sizes and high censoring rates also harm CausalCoxMGM’s reliability in recovering the ground truth. With fewer than 250 samples or rare events, graph recovery, especially edge orientation, becomes unreliable. Nevertheless, even under these unfavorable conditions, CausalCoxMGM identifies robust conditional dependencies with high precision (i.e., low false discovery rate), making it a principled tool for hypothesis generation in exploratory analyses. To assess the stability and significance of recovered adjacencies and edge orientations, strategies such as bootstrapping can be employed.

Our analysis of breast cancer progression likewise has limitations affecting generalizability. Because it is based on observational data, we cannot definitively establish causal relationships for the clinical and gene expression signatures linked to breast cancer progression. Additionally, our analysis of the METABRIC dataset was limited to complete cases, which may introduce selection bias. While the FCI causal discovery algorithm is asymptotically consistent even in the presence of latent confounders and selection bias, complete-case analysis can still bias the estimates of effect sizes. To mitigate this, we re-estimated our predictive model using multiple imputation and inverse probability weighting (Supplementary Fig. S10) and found no substantial deviations from our complete-case analysis. Finally, although our predictive model demonstrated strong discrimination across diverse external validation cohorts, its calibration was poor, likely reflecting shifts in baseline hazards between the older METABRIC dataset and more recent external validation cohorts. This suggests that recalibration on a contemporary cohort would be necessary before it could be widely applied.

As the first method capable of causal modeling in heterogeneous datasets with censored outcomes, CausalCoxMGM provides a valuable framework for clinical data analysis. By identifying robust associations and generating clinically relevant hypotheses, this approach will enable future studies to refine treatment strategies, identify novel intervention targets, and provide interpretable prognoses. Furthermore, CausalCoxMGM’s flexibility makes it adaptable to other fields that utilize censored data, such as credit risk assessment.

## Availability of source code and requirements

Project name: CausalCoxMGM

CRAN repository: https://cran.r-project.org/web/packages/rCausalMGM/index.html

Project homepage: https://github.com/tyler-lovelace1/CausalCoxMGM

Operating system: Platform independent

Programming language: R

Other requirements: R (≥4.4.0)

License: GPL-3.0 license

RRID:SCR_026906

## Additional files

**Supplementary Figure S1**: Distinct states and possible transitions in the subtype-specific multi-state models constructed for breast cancer progression. All individuals at baseline begin in the post-surgery state, and can transition to locoregional relapse, distant relapse, disease-specific death (DSS), or death by other causes (OD) along the arrows included in the model. Arrows with shared colors have a shared baseline hazard, and the probability of transitioning to each outcome is modeled with a Cox proportional hazards model conditioned on the Markov blanket of each outcome in each subtype.

**Supplementary Figure S2**: Results for CoxMGM's adjacency recovery in simulated datasets under different hyperparameter selection strategies. *F*1 scores are presented across all edges (ALL), continuous-censored edges (SC), and discrete-censored edges (SD). Four different model selection strategies are displayed: BIC, StARS, StEPS, and an oracle where the single lambda model with best *F*1 score is selected for each simulation. When sample size is varied (A), the number of features is 110 and degree is 4. When the number of features is varied (B), the sample size is 500, and the degree is 4. When the degree is varied (C), the sample size is 500, and the number of features is 110. Error bars denote 95% confidence intervals.

**Supplementary Figure S3**: Precision–recall curves for CausalCoxMGM's adjacency recovery in simulated datasets. These curves represent the average of the curves used to compute the AUPRC values in Fig. 2. Precision–recall curves are presented across all edges (ALL), continuous-censored edges (SC), and discrete-censored edges (SD). When sample size is varied (A), the number of features is 110 and degree is 4. When the number of features is varied (B), the sample size is 500, and the degree is 4. When the degree is varied (C), the sample size is 500, and the number of features is 110. Error bars denote 95% confidence intervals.

**Supplementary Figure S4**: Comparison of the ability of CausalCoxMGM and LASSO Cox regression to perform feature selection in simulated datasets. Methods compared are the direct neighbors found by CausalCoxMGM (Causal NB), the Markov blanket found by CausalCoxMGM (Causal MB), the nonzero features in LASSO Cox regression models at the 10-fold cross-validation minimum deviance (LASSO Min) and one standard error rule (LASSO 1SE). (A) Feature selection *F*1 scores across simulation conditions. (B) Feature selection precision across simulation conditions. When sample size is varied, the number of features is 110 and degree is 4. When the number of features is varied, the sample size is 500, and the degree is 4. When the degree is varied, the sample size is 500, and the number of features is 110. Error bars denote 95% confidence intervals.

**Supplementary Figure S5**: Causal model of survival in individuals with systolic heart failure recovers expected interactions. (A) Subgraph of the CausalCoxMGM model containing survival and its MB. Orange nodes are censored outcomes, green nodes are clinical features, blue nodes are pulmonary stress testing features, pink nodes are treatment features, and purple nodes are serum biomarker features. A directed edge X → Y indicates X is a cause of Y, a bidirected edge X ↔ Y indicates X and Y have a shared latent confounder, a partially oriented edge X o→ Y indicates Y is not a cause of X but it is unclear whether X causes Y or they share a latent confounder, and an unoriented edge X o-o Y indicates that causal orientation cannot be inferred for that edge. (B) Forest plot depicting the standardized hazard ratios (and 95% confidence intervals) from a multivariate Cox regression model for survival given its MB.

**Supplementary Figure S6**: Causal model of survival and time-to-discharge in individuals hospitalized with AMI recovers expected interactions. (A) Full CausalCoxMGM model constructed from the *whas500* dataset. Orange nodes are censored outcomes, green nodes are clinical features, brown nodes are demographic features, and blue nodes are complications. A directed edge X → Y indicates X is a cause of Y, a bidirected edge X ↔ Y indicates X and Y have a shared latent confounder, a partially oriented edge X o→ Y indicates Y is not a cause of X but it is unclear whether X causes Y or they share a latent confounder, and an unoriented edge X o-o Y indicates that causal orientation cannot be inferred for that edge. (B) Forest plot depicting the standardized hazard ratios (and 95% confidence intervals) from a multivariate Cox regression model of survival given its MB. (C) Forest plot depicting the standardized hazard ratios (and 95% confidence intervals) from a multivariate Cox regression model of time-to-discharge given its MB. Note that in this model, negative hazard ratios correspond to longer time-to-discharge.

**Supplementary Figure S7**: Calibration of survival probability in internal and external validation for the causal multi-state model (Causal MSM) based on CausalCoxMGM features, compared to LASSO Cox regression and random survival forest (RSF) baselines. (A) Five-year Brier scores for breast cancer outcomes in the METABRIC Study, estimated with 10-fold cross-validation. (B) Five-year Brier scores for breast cancer outcomes in METABRIC (estimated through 10-fold cross-validation, identical to panel A) and an external Meta Cohort. Error bars in (A, B) represent 95% confidence intervals. (C) Calibration plot of predicted overall survival in the SCAN-B cohort (GSE96058) for the Causal MSM model, stratified by estrogen receptor (ER) status. Individuals were grouped into deciles by predicted risk within each ER stratum (Positive, Negative, All). Mean predicted survival probabilities per decile are plotted against the actual survival probability from Kaplan Meier estimates.

**Supplementary Figure S8**: Single-cell reference-based bulk deconvolution of METABRIC gene expression. (A) Proportion of each cell type in each bulk gene expression sample split by estrogen receptor subtype. (B) Normalized expression of ER+ breast cancer progression-linked genes coming from each cell type. (C) Normalized expression of ER− breast cancer progression-linked genes coming from each cell type.

**Supplementary Figure S9**: Kaplan–Meier plots depicting the relationship between VCAM1 expression and distant relapse in ER− tumors. (A) Probability of distant relapse in ER− tumors with high and low VCAM1 expression in the METABRIC dataset. (B) Probability of distant relapse-free survival in ER tumors with high and low VCAM1 expression in external validation cohorts. VCAM1 expression values were thresholded to maximize the log-rank test statistic.

**Supplementary Figure S10**: Forest plots depicting the standardized hazard ratios (and 95% confidence intervals) from a multivariate Cox regression model for the MB of each mode of breast cancer progression under different strategies for handling missing data. Standardized hazard ratios for features selected by both the ER+ (red) and ER− (blue) models are estimated in ER+ (right) and ER− (left) tumors. (A) Standardized hazard ratios estimated using complete cases only (reproduced from Fig. 5C). (B) Standardized hazard ratios estimated with missing values imputed by missForest. (C) Standardized hazard ratios estimated using inverse probability weighting. Effect estimates of the causal factors identified by CausalCoxMGM remain consistent across methods.

**Supplementary Table S1**: Definition of true positives (TP), false positives (FP), true negatives (TN), and false negatives (FN) for the evaluation of adjacency and orientation accuracy in causal graphical models [8]. Used in the calculation of precision, recall, and *F*_1_ scores.

## Supplementary Material

giag060_Supplemental_Files

giag060_Authors_Response_To_Reviewer_Comments_original_submission

giag060_GIGA-D-25-00149_original_submission

giag060_GIGA-D-25-00149_revision_1

giag060_Reviewer_1_Report_original_submission Reviewer 1 -- 6/10/2025

giag060_Reviewer_1_Report_revision_1 Reviewer 1 -- 8/25/2025

giag060_Reviewer_2_Report_original_submission Reviewer 2 -- 6/21/2025

giag060_Reviewer_2_Report_revision_1 Reviewer 2 -- 1/6/2026

## Data Availability

We used only publicly available datasets and simulated data we generated. The datasets supporting the results of this article are available in the following repositories (see also Supplementary Methods). The rCausalMGM package is also available on CRAN [66]. All additional Supplementary Material [67] is available in the *GigaScience* repository, GigaDB [68].
