# Supplementary material for: New causal discovery algorithm over censored variables identifies subtype-specific drivers of breast cancer progression
Source: Gigascience. 2026 May 22;15:giag060. doi: 10.1093/gigascience/giag060 (PMC13235964; doi:10.1093/gigascience/giag060)
Supplement: giag060_Supplemental_Files [file giag060_supplemental_files.zip › 4a_CausalCoxMGM_Supplementary_Methods.20250204-pvb.docx]

Supplementary Methods

# Background

## Cox proportional hazards model

To model the conditional dependence of censored variables with continuous and discrete covariates in graphical models, we employ the Cox proportional hazards model^1^. This model defines a semiparametric relationship between covariates and the hazard of a censored event, where the hazard denotes the rate at which events occur at time $t$, conditioned on survival until $t$. For covariates $x$, the hazard function is expressed as:

$$h\left( t \mid x \right)=h_{0}\left( t \right)\exp\left( \beta x \right)$$

where $h_{0}\left( t \right)$ is an arbitrary baseline hazard function dependent only on $t$, and $\beta$ is a vector of regression coefficients associated with covariates $x$.

Under the proportional hazards’ assumption, the ratio of an individual’s hazard function to the baseline is constant over time. This allows estimation of $\beta$ independently of $h_{0}\left( t \right)$ by maximizing the partial log-likelihood:

$$l\left( \beta;x \right)=\sum_{i=1}^{n} \delta_{i}\left( \beta x_{i}-\log\sum_{u\in\mathcal{R}_{\mathcal{i}}} \exp\left( \beta x_{u} \right) \right)$$

where $\delta_{i}$ is an event indicator (1 if uncensored), and $\mathcal{R}_{\mathcal{i}}$ is the risk set of individual $i$, containing all individuals who have not yet experienced the event or been censored before $t_{i}$, including $i$ itself.

To compute penalized estimates of $\beta$, prior work approximates the partial log-likelihood with a second-order Taylor expansion, enabling an iteratively reweighted least squares (IRLS) solution^2^. Defining the log hazard ratio as $\eta=\beta x$, the gradient and Hessian with respect to $\eta$ are $l^{'}\left( \eta\right)$ and $l^{''}\left( \eta\right)$. For the current estimate $\hat{\eta}=\hat{\beta}x$, we approximate the partial log-likelihood as:

$$l\left( \beta;x \right)=\frac{1}{2}\left( z-\beta x \right)^{T}W\left( z-\beta x \right)$$

where $z=\hat{\eta}-l^{''}\left( \hat{\eta} \right)^{-1}l^{'}\left( \hat{\eta} \right)$ and $W$ is a diagonal weight matrix with $\text{diag}\left( W \right)=\text{diag}\left( l^{''}\left( \hat{\eta} \right) \right)$. For both $z$ and $W$, only the diagonal of the Hessian is used as it is more computationally efficient and the off-diagonals are negligible^2^.

## Mixed Graphical Models

A Mixed Graphical Model (MGM) is a type of undirected graphical model proposed by Lee and Hastie that can represent conditional independence relations between variables in a dataset that contains a mixture of continuous and discrete variables^3^. Given a dataset with $p$ continuous and $q$ discrete variables, the joint distribution over the dataset is given by

$$P\left( x,y;\Theta\right)\propto\exp\left( \sum_{s=1}^{p} \sum_{t=1}^{p} -\frac{1}{2}\beta_{st}x_{s}x_{t}+\sum_{s=1}^{p} \alpha_{s}x_{s}+\sum_{s=1}^{p} \sum_{j=1}^{q} \rho_{sj}\left( y_{j} \right)x_{s}+\sum_{j=1}^{q} \sum_{k=1}^{q} \phi_{jk}\left( y_{j},y_{k} \right) \right)$$

In this model, $\Theta$ represents the full set of parameters, $x_{s}$ represents the $s$th of $p$ continuous variables and $y_{j}$ represents the $j$th of $q$ discrete variables. The parameter $\beta_{st}$ represents the edge potential between the continuous variables $s$ and $t$, $\alpha_{s}$ represents the node potential for the continuous variable $s$, $\rho_{sj}$ represents the edge potential between the continuous variable $s$ and the discrete variable $j$, and $\phi_{jk}$ represents the edge potential between the discrete variables $j$ and $k$. This parameterization has the property that the conditional distributions for continuous and discrete variables are given by Gaussian linear regressions and multinomial logistic regressions, respectively. To avoid the computationally expensive calculation of the partition function they minimize the negative log pseudolikelihood, given below:

$$\tilde{l}\left( \Theta\mid x,y \right)=-\sum_{s=1}^{p} \log p\left( x_{s} \mid x_{\setminus s},y;\Theta\right)-\sum_{j=1}^{q} \log p\left( y_{j} \mid x,y_{\setminus j};\Theta\right)$$

To promote sparsity, a penalized form of the negative log pseudolikelihood is learned using proximal gradient descent^4^. The negative log pseudolikelihood is optimized with separate regularization parameters for each type of edge, where $\lambda_{cc}$ penalizes continuous-continuous edges, $\lambda_{cd}$ penalizes continuous-discrete edges, and $\lambda_{dd}$ penalizes discrete-discrete edges.

$\min_{\lambda} \tilde{l_{\lambda}}\left( \Theta\right)=\tilde{l}\left( \Theta\right)+\lambda_{cc}\sum_{s=1}^{p} \sum_{t=1}^{s-1} \left| \beta_{st} \right|+\lambda_{cd}\sum_{s=1}^{p} \sum_{j=1}^{q} \parallel\rho_{sj}\parallel_{2}+\lambda_{dd}\sum_{j=1}^{q} \sum_{k=1}^{j-1} \parallel\phi_{jk}\parallel_{F}$

## An independence test for mixed data

Constraint-based causal inference algorithms require a reliable conditional independence test to learn causal orientations. For mixed datasets containing both continuous and discrete variables, a conditional independence test based on linear and multinomial logistic regression has been developed^5^. First, all discrete variables, which we denote as having $K$ categories, are transformed into $K-1$ binary indicator values. Then, the regression models mentioned above are used to test whether a pair of variables $X$ and $Y$ are independent given a conditioning set $S$. In the case that both $X$ and $Y$ are continuous, a linear regression of $X$ onto $Y$ and $S$ is performed using a $t$-test on the coefficient of $Y$ to calculate its $p$ value. In the case that $X$ is discrete, a multinomial logistic regression of $X$ onto $S$ is performed (the null model) and $X$ onto $Y$ and $S$. The $p$ value is calculated from the likelihood ratio test between the two multinomial logistic regression models. Alternatively, if $Y$ is discrete and $X$ is continuous, then $X$ and $Y$ are flipped and the same procedure as the one above is performed. Once the $p$ value is calculated, if it is below the specified threshold $\alpha$ the null hypothesis that $X$ and $Y$ are independent given the conditioning set $S$ is rejected.

# Dataset preparation and analysis

## Simulated network data

To assess the performance of the proposed method, 20 Erdős–Rényi (ER; random) and 20 scale-free (SF) directed acyclic graphs (DAGs) were generated with 55, 110, or 550 nodes (5/11 continuous, 5/11 discrete, and 1/11 censored) with an edge degree of 2, 4, or 6. The data was simulated as a structural equation model: continuous nodes were simulated as a linear function of their parents with additive Gaussian noise, discrete nodes were sampled from a multinomial distribution with probabilities defined as a multinomial logistic regression on their parents, and censored nodes were sampled from a proportional hazards model with a Weibull baseline hazard and hazard ratios determined by their parents, as described in ^6^. All effect sizes in the model were drawn from $\mathcal{U}_{\left[ -1.5,-0.5 \right]\cup\left[ 0.5,1.5 \right]}$, while the standard deviation of the Gaussian noise for linear variables was sampled from $\mathcal{U}_{\left[ 1,2 \right]}$. The shape parameter $\nu$ for the Weibull distribution was sampled from $\mathcal{U}_{\left[ 2,10 \right]}$, while the scale parameter $\lambda$ was selected to give the baseline hazard an expected survival time of 1000. To assess the performance of the method under different censoring conditions, datasets were generated under light censoring where approximately 30% of samples were censored (70% observed events), and heavy censoring where approximately 70% of samples were censored (30% observed events). For both censoring conditions, half of the individuals are censored due to an end-of-study censoring, where any individuals with events after the end-of-study cutoff is censored at the cutoff value. The other half are censored due to lost-to-follow-up censoring, with censoring times sampled uniformly from the time of the earliest event to the time of the event of the individual selected for censoring. This approach allows us to incorporate the two types of right-censored data into our simulations^7^. For each of the 20 DAGs, datasets were created with sample sizes of 100, 250, 500, 1000, 5000, and 10000 to enable us to assess the performance of these methods across sample sizes.

## Evaluation of causal graph recovery

To evaluate causal graph recovery performance, we compare the estimated Markov equivalence classes of the causal networks to the true causal DAG and its Markov equivalence class. The Markov equivalence class (MEC) is defined as the set of all DAGs that represent the same set of conditional independence relationships in the dataset. For example, in the three variable DAG $X \to Y \to Z$, the only conditional independence relationship represented by the DAG is $X \perp Z \mid Y$. However, this same conditional independence relationship is also represented by the DAGs $X \leftarrow Y \to Z$ and $X \leftarrow Y \leftarrow Z$. In cases where different edge orientations result in the same set of conditional relationships, the MEC graph contains an undirected edge, such as $X - Y - Z$, as either orientation is valid in terms of the conditional independence relationships represented by the graph. Thus, MECs share the same variables, adjacencies, and conditional independence relationships as the true causal graph.

We use standard statistics to assess how well CoxMGM and CausalCoxMGM recover both adjacencies and orientations in the true causal DAG. The precision, recall, and F_1_ score are defined below:

$$Precision = \frac{TP}{TP + FP}, Recall = \frac{TP}{TP + FN}, F_{1}=\frac{2*TP}{2*TP + FP + FN}$$

with the definition of true positives (TP), false positives (FP), true negatives (TN), and false negatives (FN) for both adjacencies and orientations defined by Kummerfeld *et al.*^8^ and reproduced in **Table S1**. Precision measures the ratio of the number of correctly predicted edges over the total number of predicted edges, while recall measures the ratio of the number of correctly predicted edges over the number of true edges in the graph. The F_1_ score represents the overall recovery performance on a single graph by taking the harmonic mean of the precision and recall. As CoxMGM learns an undirected graphical model representing the moralized graph of the true causal DAG, we only compute the adjacency precision, recall, and F1 score when evaluating CoxMGM graph recovery. Additionally, because the moralized graph includes additional adjacencies connecting the parents of colliders, a modified version of the adjacency precision, recall, and F_1_ score is computed. As the inclusion of moralizing edges are not errors, they are included as true positives in the calculation of the precision of CoxMGM. However, as the exclusion of these edges does not harm the subsequent recovery of the causal adjacencies, recall is calculated based on the causal skeleton as described in **Table S1**.For both adjacency and orientation recovery, we utilize the area under the precision-recall curve (AUPRC) as a summary metric of the overall predictive power of our methods across across a range of hyperparameter values ($\lambda$ for CoxMGM and $\alpha$ for CausalCoxMGM).

Finally, to assess the overall ability of CausalCoxMGM to recover the MEC of the true causal graph, we measure the Structural Hamming Distance (SHD) between the estimated and true MEC. The SHD is a distance metric between two graphs, that represents the number of edge insertions, deletions, and orientation changes needed to convert one graph to another. To make the SHD comparable across simulation conditions, the SHD is normalized by dividing by the number of edges in the true causal graph.

**Supplementary Table S1**: Definition of true positives (TP), false positives (FP), true negatives (TN), and false negatives (FN) for the evaluation of adjacency and orientation accuracy in causal graphical models^8^. Used in the calculation of precision, recall, and F_1_ scores.

| True Edge | Predicted Edge | Adjacency Evaluation | Orientation Evaluation |
| --- | --- | --- | --- |
| X 🡪 Y | X 🡪 Y | TP | TP, TN |
|  | X 🡨 Y | TP | FP, FN |
|  | X ­— Y | TP | FN |
|  | X … Y | FN | FN |
| X … Y | X 🡪 Y | FP | FP |
|  | X 🡨 Y | FP | FP |
|  | X — Y | FP |  |
|  | X … Y | TN |  |

## Construction and evaluation of predictive models

Predictive models based on CausalCoxMGM are constructed with a Cox regression model regressed on the Markov blanket (MB) of each individual outcome. The MB of a target variable in a graphical model is the set of variables that, when conditioned on, render that target variable independent of all other variables in the dataset. Predictors of composite outcomes in the METABRIC dataset (*e.g.* DRFS) are constructed with a multistate model implemented with *mstate*. The state transitions included in the model (**Figure S1**) enable us to integrate the predictive models of individual outcomes (DSS, OD, LR, and DR) to predict composite outcomes. Baseline predictive models constructed with LASSO Cox regression and random survival forests, were learned using *glmnet* and *randomForestSRC* respectively. For these models, predictors of composite outcomes are learned directly. During internal validation, the predictive accuracy of each model is assessed with Harrell’s concordance statistic^9^ across 10 cross-validation folds. For external validation, Harrell’s concordance statistics were computed for each external dataset individually and then combined into a summary statistic (the Meta Cohort) with *survcomp*.

## Analysis of real-world cardiovascular disease datasets

Two low-dimensional cardiovascular disease datasets were used to construct causal models with censored outcomes. The first dataset, *peakVO2*^10^, included 39 baseline clinical, demographic, and exercise stress testing features and all-cause mortality collected from individuals with systolic heart failure. A CausalCoxMGM model was constructed utilizing all features available from the peakVO2 dataset available from the randomForestSRC package^11^. Hyperparameter values for $\lambda$ and $\alpha$ were selected using 5-fold cross-validation to select the model with the highest test likelihood for all-cause mortality. The second dataset, *whas500*^12^, is a small cohort constructed with 500 patients taken from the Worcester Heart Attack Study^13^. This dataset contains 13 clinical and demographic features recorded after hospitalization for an acute myocardial infarction, as well as two censored outcomes: time-to-discharge and all-cause mortality. We note that death prior to discharge corresponds to censoring in the time-to-discharge outcome. Again, the hyperparameter values for $\lambda$ and $\alpha$ were selected using 5-fold cross-validation to identify the model with the highest test likelihood for the censored outcomes.

## Analysis of subtype-specific breast cancer progression

The method described above is applied to identify clinical and gene expression features causally linked to breast cancer progression in the Molecular Taxonomy of Breast Cancer International Consortium (METABRIC) dataset^14^. Included in this analysis are clinical features known to be linked to breast cancer prognosis and subtype, including age at diagnosis, inferred menopausal state, tumor size, lymph node status, estrogen receptor (ER) status, progesterone receptor (PR) status, human epidermal growth factor receptor 2 (HER2) status, and histological subtype^15,16^. From the $\log_{2}$-transformed microarray gene expression data, the genes were filtered to the 500 most highly variant genes. To reduce the multicollinearity of the gene expression data, hierarchical clustering was used to identify 17 hubs of highly correlated genes with pairwise Spearman's correlations greater than 0.8, which were summarized by the gene with the highest mean correlation to the other gens in the cluster. This further reduced the dimensionality of the gene expression dataset used in the causal discovery analysis to 437 genes. Prior to being used for constructing causal models of breast cancer progression, the nonparanormal transformation^17^ is applied to all continuous variables. The nonparanormal transformation maps continuous variables to a multivariate Gaussian copula, which enables CoxMGM and the proposed conditional independence test to learn nonparametric rank-based associations rather than linear ones.

Different avenues of breast cancer progression and mortality are recorded as censored time-to-event variables in the METABRIC dataset. The censored variables directly included in our causal models are disease-specific survival (DSS), death by other causes (OD), time to distant relapse (DR), and time to locoregional relapse (LR). Other common metrics of breast cancer progression are the result of combining events from these different avenues of progression: overall survival (OS; DSS and OD), distant relapse free survival (DRFS; DSS, OD, and DR), and disease-free survival (DFS; DSS, OD, DR, and LR). As breast cancer progression results from different mechanisms and has significantly different baseline hazards for ER+ and ER- breast cancers, two causal models were learned to identify features causally linked to breast cancer progression: (1) an ER+ model, and (2) an ER- model.

To construct our stratified and subtype-specific causal models, we separately applied CoxMGM followed by FCI-Max on the split ER+ and ER- METABRIC datasets. The optimal regularization parameters $\hat{\lambda}$ for the CoxMGM were selected using the stability-based selection method StARS^18^. FCI-Max was run with false discovery control on the adjacencies^19^ with $\alpha=0.1$. From these causal models, we identify features that are potentially causally related to breast cancer progression. We also construct a predictive model using the causal neighbors of breast cancer progression features and validate its performance in external validation cohorts. The predictive model was built using a stratified multi-state Cox regression model, which enables us to model complex composite outcomes such as distant relapse-free survival while allowing different subtypes to have different baseline hazards for each transition^20^. Model performance is assessed by the concordance of the predicted risk and true survival times using Harrell's concordance statistic^9^. The external validation cohorts used in this study were selected to contain either microarray or RNA-seq gene expression data from primary breast tumors, key clinical features such as ER status, lymph node status, and tumor size, and either DSS (GSE3494^21^ and GSE45255^22^), OS (GSE42568^23^, GSE7390^24^, and GSE96058^25^), DRFS (GSE11121^26^, GSE19615^27^, GSE45255^22^, GSE6532^28^, GSE7390^24^, and GSE9195^29^), or DFS (GSE42568^23^, GSE19615^27^, GSE45255^22^, GSE6532^28^, GSE7390^24^, and GSE9195^29^). The Meta Cohort refers to summary statistics of the concordance indices for each metric of breast cancer progression computed using *survcomp*^30^.

# References

1 Cox, D. R. Regression Models and Life-Tables. *Journal of the Royal Statistical Society Series B: Statistical Methodology* **34**, 187-202 (1972). <https://doi.org:10.1111/j.2517-6161.1972.tb00899.x>

2 Simon, N., Friedman, J., Hastie, T. & Tibshirani, R. Regularization Paths for Cox's Proportional Hazards Model via Coordinate Descent. *J. Stat. Softw.* **39**, 1-13 (2011). <https://doi.org:10.18637/jss.v039.i05>

3 Lee, J. D. & Hastie, T. J. Learning the Structure of Mixed Graphical Models. *J. Comput. Graph. Stat.* **24**, 230-253 (2015). <https://doi.org:10.1080/10618600.2014.900500>

4 Beck, A. & Teboulle, M. A Fast Iterative Shrinkage-Thresholding Algorithm for Linear Inverse Problems. *SIAM Journal on Imaging Sciences* **2**, 183-202 (2009). <https://doi.org:10.1137/080716542>

5 Sedgewick, A. J. *et al.* Mixed graphical models for integrative causal analysis with application to chronic lung disease diagnosis and prognosis. *Bioinformatics* **35**, 1204-1212 (2019). <https://doi.org:10.1093/bioinformatics/bty769>

6 Bender, R., Augustin, T. & Blettner, M. Generating survival times to simulate Cox proportional hazards models. *Stat. Med.* **24**, 1713-1723 (2005). <https://doi.org:10.1002/sim.2059>

7 Leung, K. M., Elashoff, R. M. & Afifi, A. A. Censoring issues in survival analysis. *Annu Rev Public Health* **18**, 83-104 (1997). <https://doi.org:10.1146/annurev.publhealth.18.1.83>

8 Kummerfeld, E., Williams, L. & Ma, S. Power analysis for causal discovery. *International Journal of Data Science and Analytics* **17**, 289-304 (2023). <https://doi.org:10.1007/s41060-023-00399-4>

9 Harrell, F. E., Califf, R. M., Pryor, D. B., Lee, K. L. & Rosati, R. A. Evaluating the yield of medical tests. *Jama* **247**, 2543-2546 (1982).

10 Hsich, E. *et al.* Importance of treadmill exercise time as an initial prognostic screening tool in patients with systolic left ventricular dysfunction. *Circulation* **119**, 3189-3197 (2009). <https://doi.org:10.1161/CIRCULATIONAHA.109.848382>

11 Hsich, E., Gorodeski, E. Z., Blackstone, E. H., Ishwaran, H. & Lauer, M. S. Identifying important risk factors for survival in patient with systolic heart failure using random survival forests. *Circ Cardiovasc Qual Outcomes* **4**, 39-45 (2011). <https://doi.org:10.1161/CIRCOUTCOMES.110.939371>

12 Hosmer Jr, D. W., Lemeshow, S. & May, S. *Applied survival analysis: regression modeling of time-to-event data*. Vol. 618 (John Wiley & Sons, 2008).

13 Goldberg, R. J. Recent Changes in Attack and Survival Rates of Acute Myocardial Infarction (1975 Through 1981). *Jama* **255** (1986). <https://doi.org:10.1001/jama.1986.03370200076031>

14 Curtis, C. *et al.* The genomic and transcriptomic architecture of 2,000 breast tumours reveals novel subgroups. *Nature* **486**, 346-352 (2012). <https://doi.org:10.1038/nature10983>

15 Galea, M. H., Blamey, R. W., Elston, C. E. & Ellis, I. O. The Nottingham Prognostic Index in primary breast cancer. *Breast Cancer Res. Treat.* **22**, 207-219 (1992). <https://doi.org:10.1007/BF01840834>

16 Rakha, E. A. *et al.* Nottingham Prognostic Index Plus (NPI+): a modern clinical decision making tool in breast cancer. *Br. J. Cancer* **110**, 1688-1697 (2014). <https://doi.org:10.1038/bjc.2014.120>

17 Liu, H., Lafferty, J. & Wasserman, L. The nonparanormal: Semiparametric estimation of high dimensional undirected graphs. *J. Mach. Learn. Res.* (2009).

18 Liu, H., Roeder, K. & Wasserman, L. Stability approach to regularization selection (StARS) for high dimensional graphical models. *Adv. Neural Inf. Process. Syst.* **24**, 1432-1440 (2010).

19 Li, J. & Wang, Z. J. Controlling the false discovery rate of the association/causality structure learned with the PC algorithm. *Journal of Machine Learning Research* **10** (2009).

20 Putter, H., Fiocco, M. & Geskus, R. B. Tutorial in biostatistics: competing risks and multi-state models. *Stat Med* **26**, 2389-2430 (2007). <https://doi.org:10.1002/sim.2712>

21 Miller, L. D. *et al.* An expression signature for p53 status in human breast cancer predicts mutation status, transcriptional effects, and patient survival. *Proc. Natl. Acad. Sci. U. S. A.* **102**, 13550-13555 (2005). <https://doi.org:10.1073/pnas.0506230102>

22 Nagalla, S. *et al.* Interactions between immunity, proliferation and molecular subtype in breast cancer prognosis. *Genome Biol.* **14**, R34 (2013). <https://doi.org:10.1186/gb-2013-14-4-r34>

23 Clarke, C. *et al.* Correlating transcriptional networks to breast cancer survival: a large-scale coexpression analysis. *Carcinogenesis* **34**, 2300-2308 (2013). <https://doi.org:10.1093/carcin/bgt208>

24 Desmedt, C. *et al.* Strong time dependence of the 76-gene prognostic signature for node-negative breast cancer patients in the TRANSBIG multicenter independent validation series. *Clin Cancer Res* **13**, 3207-3214 (2007). <https://doi.org:10.1158/1078-0432.CCR-06-2765>

25 Brueffer, C. *et al.* Clinical value of RNA sequencing-based classifiers for prediction of the five conventional breast cancer biomarkers: A report from the population-based multicenter Sweden Cancerome Analysis Network-breast initiative. *JCO Precis. Oncol.* **2** (2018). <https://doi.org:10.1200/PO.17.00135>

26 Schmidt, M. *et al.* The humoral immune system has a key prognostic impact in node-negative breast cancer. *Cancer Res* **68**, 5405-5413 (2008). <https://doi.org:10.1158/0008-5472.CAN-07-5206>

27 Li, Y. *et al.* Amplification of LAPTM4B and YWHAZ contributes to chemotherapy resistance and recurrence of breast cancer. *Nat Med* **16**, 214-218 (2010). <https://doi.org:10.1038/nm.2090>

28 Loi, S. *et al.* Definition of clinically distinct molecular subtypes in estrogen receptor-positive breast carcinomas through genomic grade. *J Clin Oncol* **25**, 1239-1246 (2007). <https://doi.org:10.1200/JCO.2006.07.1522>

29 Loi, S. *et al.* Predicting prognosis using molecular profiling in estrogen receptor-positive breast cancer treated with tamoxifen. *BMC Genomics* **9**, 239 (2008). <https://doi.org:10.1186/1471-2164-9-239>

30 Schröder, M. S., Culhane, A. C., Quackenbush, J. & Haibe-Kains, B. survcomp: an R/Bioconductor package for performance assessment and comparison of survival models. *Bioinformatics* **27**, 3206-3208 (2011). <https://doi.org:10.1093/bioinformatics/btr511>
