# Supplementary material for: New causal discovery algorithm over censored variables identifies subtype-specific drivers of breast cancer progression
Source: Gigascience. 2026 May 22;15:giag060. doi: 10.1093/gigascience/giag060 (PMC13235964; doi:10.1093/gigascience/giag060)
Supplement: giag060_Supplemental_Files [file giag060_supplemental_files.zip › 4b_CausalCoxMGM_Suppl_Figures.20250204-pvb.pdf]

SUPPLEMENTARY FIGURES

Supplement: State transitions in the multi-state model of breast cancer progression

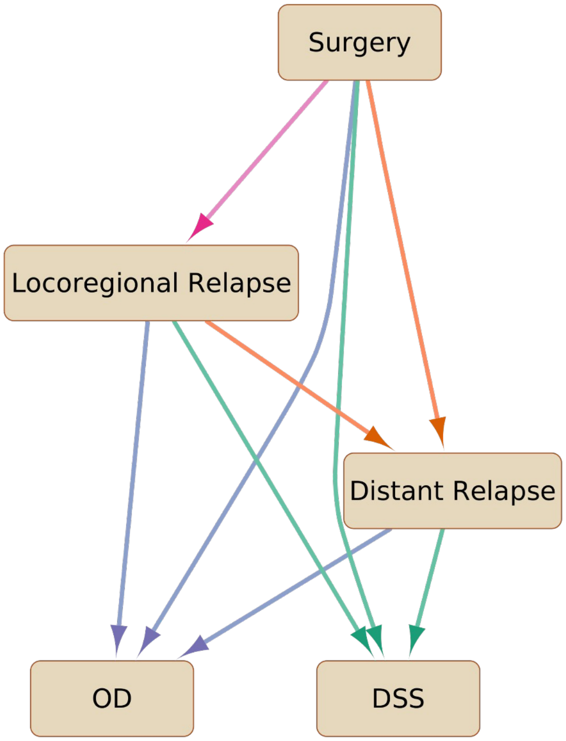

Supplementary Figure S1: Distinct states and possible transitions in the subtype-specific multi-state models constructed for breast cancer progression. All individuals at baseline begin in the post-surgery state, and can transition to Locoregional relapse, distant relapse, disease-specific death (DSS), or death by other causes (OD) along the arrows included in the model. Arrows with shared colors have a shared baseline hazard, and the probability of transitioning to each outcome is modeled with a Cox proportional hazards model conditioned on the Markov blanket of each outcome in each subtype.

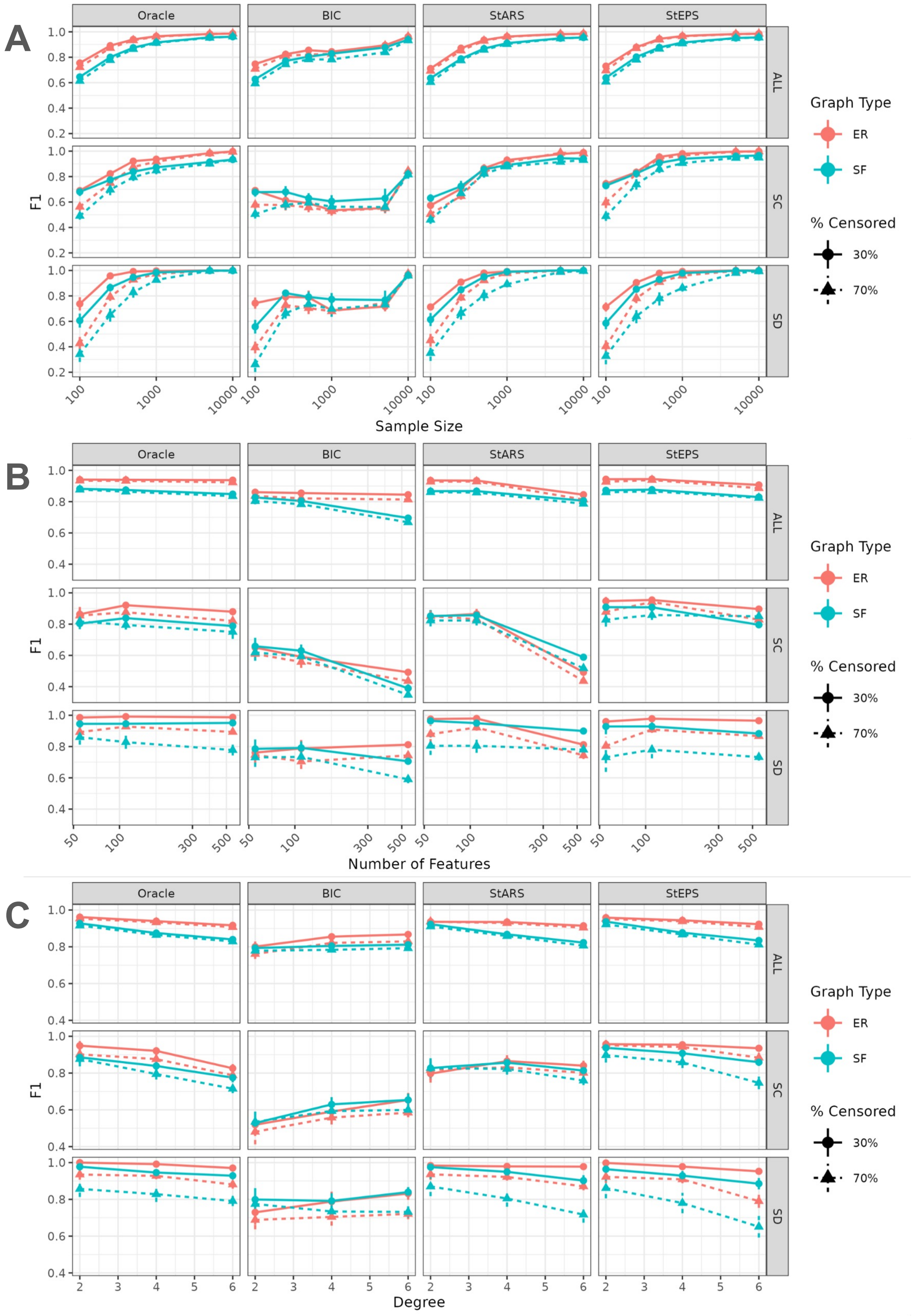

Supplementary Figure S2: Results for CoxMGM's adjacency recovery in simulated datasets under different hyperparameter selection strategies. F_1 scores are presented across all edges (ALL), continuous-censored edges (SC), and discrete-censored edges (SD). Four different model selection strategies are displayed: BIC, StARS, StEPS, and an oracle where the single lambda model with best F_1 score is selected for each simulation. When sample size is varied (**A**), the number of features is 110 and degree is 4. When the number of features is varied (**B**), the sample size is 500 and the degree is 4. When the degree is varied (**C**), the sample size is 500 and the number of features is 110. Error bars denote 95% confidence intervals.

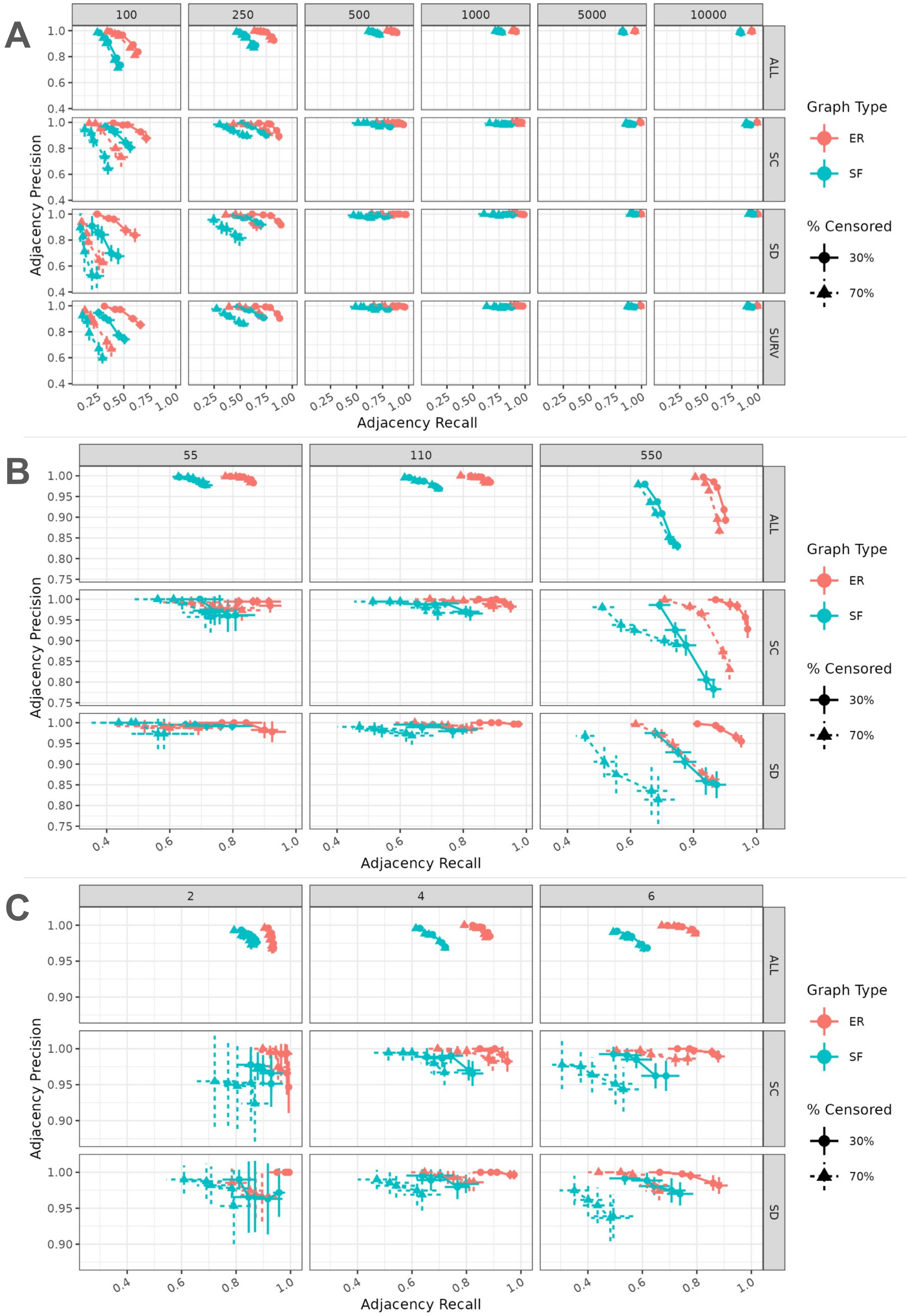

Supplementary Figure S3: Precision-recall curves for CausalCoxMGM's adjacency recovery in simulated datasets. These curves represent the average of the curves used to compute the AUPRC values in **Figure 2**. Precision-recall curves are presented across all edges (ALL), continuous-censored edges (SC), and discrete-censored edges (SD). When sample size is varied (**A**), the number of features is 110 and degree is 4. When the number of features is varied (**B**), the sample size is 500 and the degree is 4. When the degree is varied (**C**), the sample size is 500 and the number of features is 110. Error bars denote 95% confidence intervals.

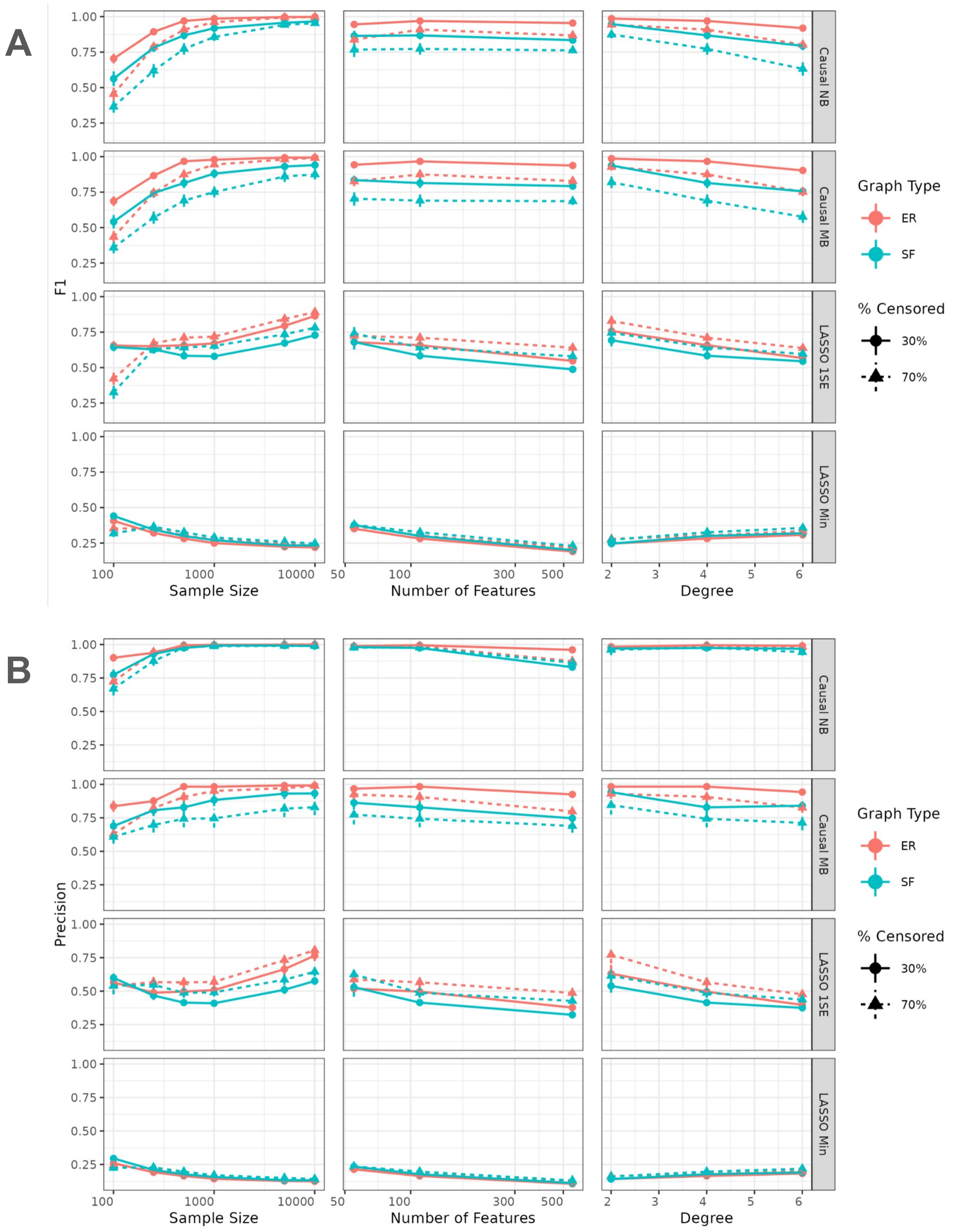

Supplementary Figure S4: Comparison of the ability of CausalCoxMGM and LASSO Cox regression to perform feature selection in simulated datasets. Methods compared are the direct neighbors found by CausalCoxMGM (Causal NB), the Markov blanket found by CausalCoxMGM (Causal MB), the nonzero features in LASSO Cox regression models at the 10-fold cross-validation minimum deviance (LASSO Min) and one standard error rule (LASSO 1SE). (**A**) Feature selection F_1 scores across simulation conditions. (**B**) Feature selection precision across simulation conditions. When sample size is varied, the number of features is 110 and degree is 4. When the number of features is varied, the sample size is 500 and the degree is 4. When the degree is varied, the sample size is 500 and the number of features is 110. Error bars denote 95% confidence intervals.

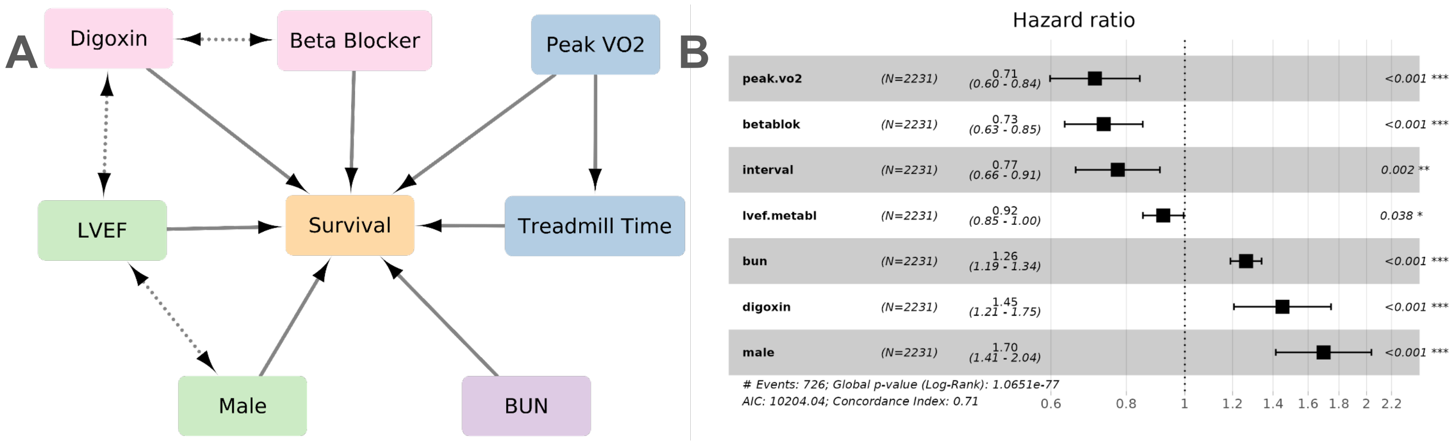

Supplementary Figure S5: Causal model of survival in individuals with systolic heart failure recovers expected interactions. (**A**) Subgraph of the CausalCoxMGM model containing survival and its MB. Orange nodes are censored outcomes, green nodes are clinical features, blue nodes are pulmonary stress testing features, pink nodes are treatment features, and purple nodes are serum biomarker features. A directed edge X → Y indicates X is a cause of Y, a bidirected edge X ↔ Y indicates X and Y have a shared latent confounder, a partially oriented edge X o→ Y indicates Y is not a cause of X but it is unclear whether X causes Y or they share a latent confounder, and an unoriented edge X o-o Y indicates that causal orientation cannot be inferred for that edge. (**B**) Forest plot depicting the standardized hazard ratios (and 95% confidence intervals) from a multivariate Cox regression model for survival given its MB.

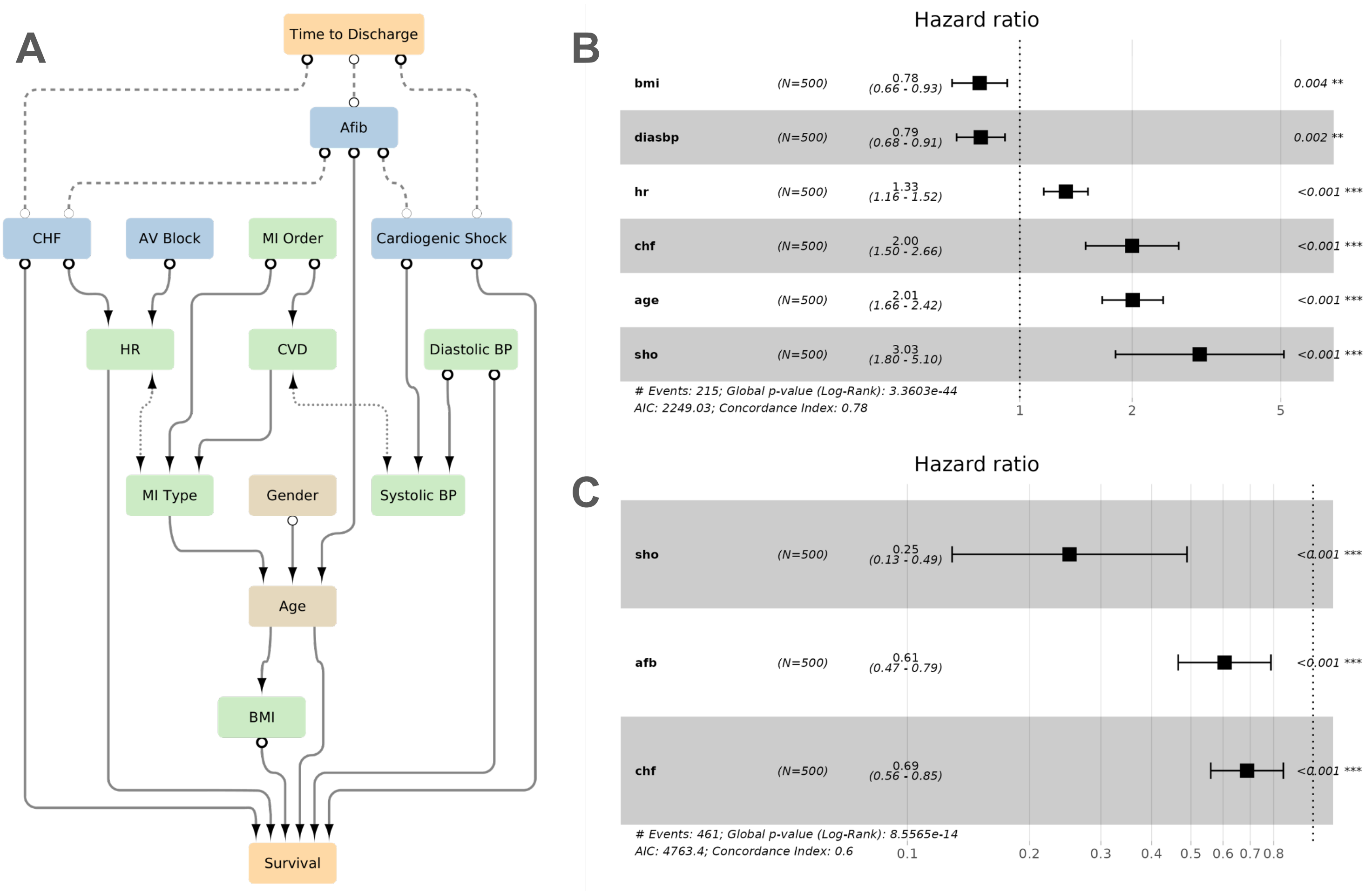

Supplementary Figure S6: Causal model of survival and time-to-discharge in individuals hospitalized with AMI recovers expected interactions. (**A**) Full CausalCoxMGM model constructed from the *whas500* dataset. Orange nodes are censored outcomes, green nodes are clinical features, brown nodes are demographic features, and blue nodes are complications. A directed edge X → Y indicates X is a cause of Y, a bidirected edge X ↔ Y indicates X and Y have a shared latent confounder, a partially oriented edge X o→ Y indicates Y is not a cause of X but it is unclear whether X causes Y or they share a latent confounder, and an unoriented edge X o-o Y indicates that causal orientation cannot be inferred for that edge. (**B**) Forest plot depicting the standardized hazard ratios (and 95% confidence intervals) from a multivariate Cox regression model of survival given its MB. (**C**) Forest plot depicting the standardized hazard ratios (and 95% confidence intervals) from a multivariate Cox regression model of time-to-discharge given its MB. Note that in this model negative hazard ratios correspond to longer time-to-discharge.

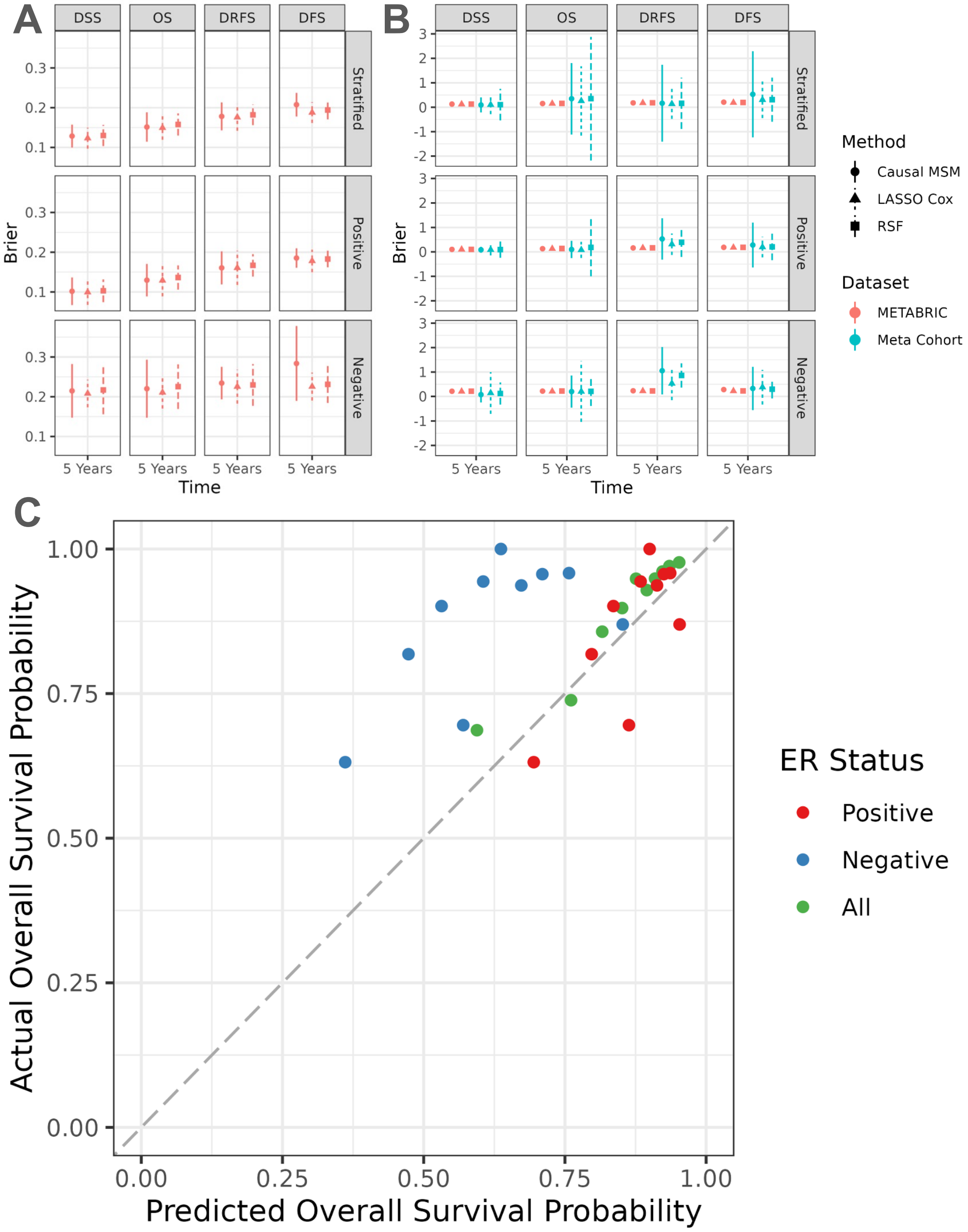

Supplementary Figure S7: Calibration of survival probability in internal and external validation for the causal multi-state model (Causal MSM) based on CausalCoxMGM features, compared to LASSO Cox regression and Random Survival Forest (RSF) baselines. (**A**) Five-year Brier scores for breast cancer outcomes in the METABRIC Study, estimated with 10-fold cross-validation. (**B**) Five-year Brier scores for breast cancer outcomes in METABRIC (estimated through 10-fold cross-validation, identical to panel **A**) and an external Meta Cohort. Error bars in (**AB**) represent 95% confidence intervals. (**C**) Calibration plot of predicted overall survival in the SCAN-B cohort (GSE96058) for the Causal MSM model, stratified by estrogen receptor (ER) status. Individuals were grouped into deciles by predicted risk within each ER stratum (Positive, Negative, All). Mean predicted survival probabilities per decile are plotted against the actual survival probability from Kaplan Meier estimates.

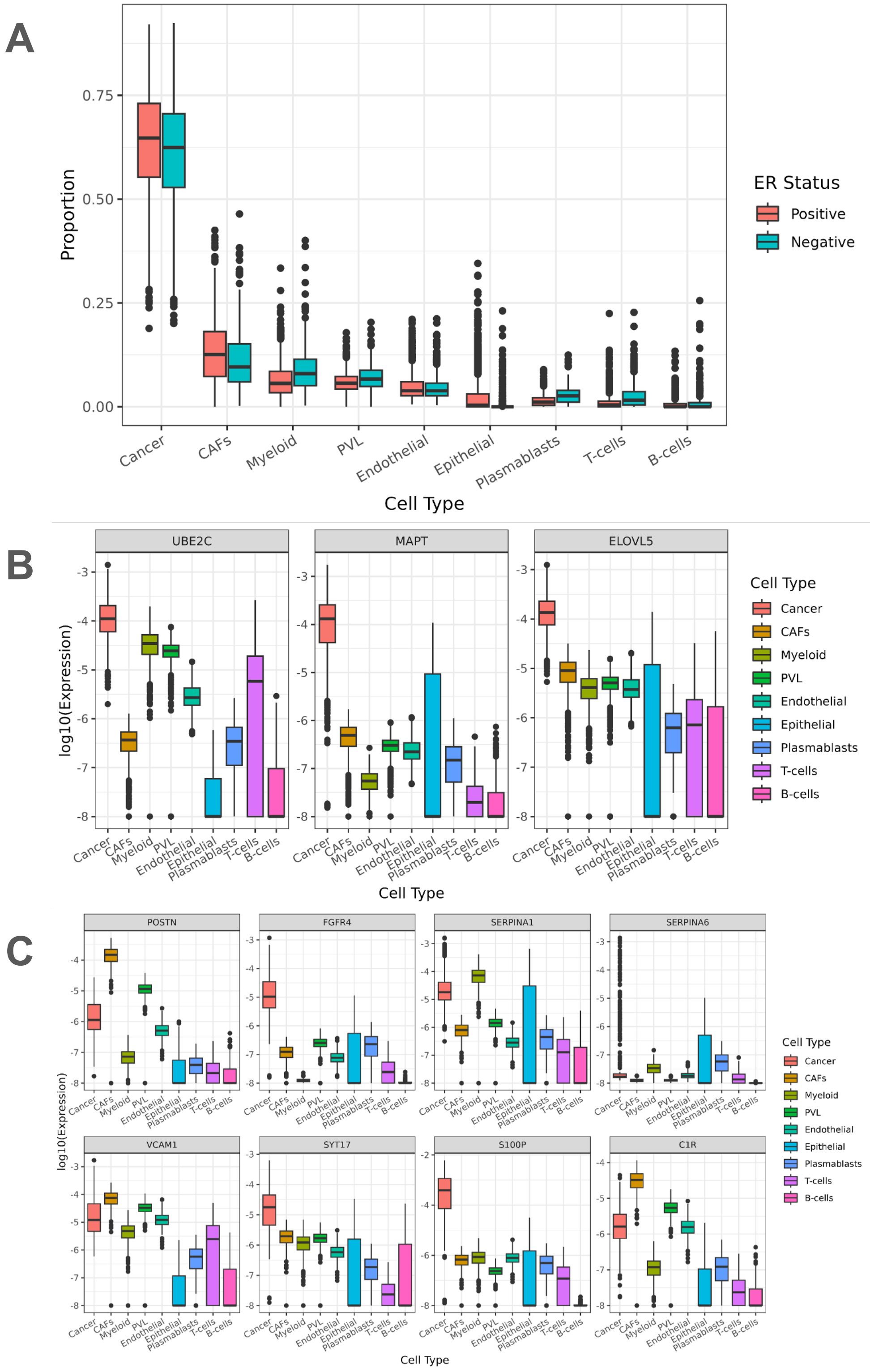

Supplementary Figure S8: Single-cell reference-based bulk deconvolution of METABRIC gene expression. (**A**) Proportion of each cell-type in each bulk gene expression sample split by estrogen receptor subtype. (**B**) Normalized expression of ER+ breast cancer progression-linked genes coming from each cell-type. (**C**) Normalized expression of ER- breast cancer progression-linked genes coming from each cell-type.

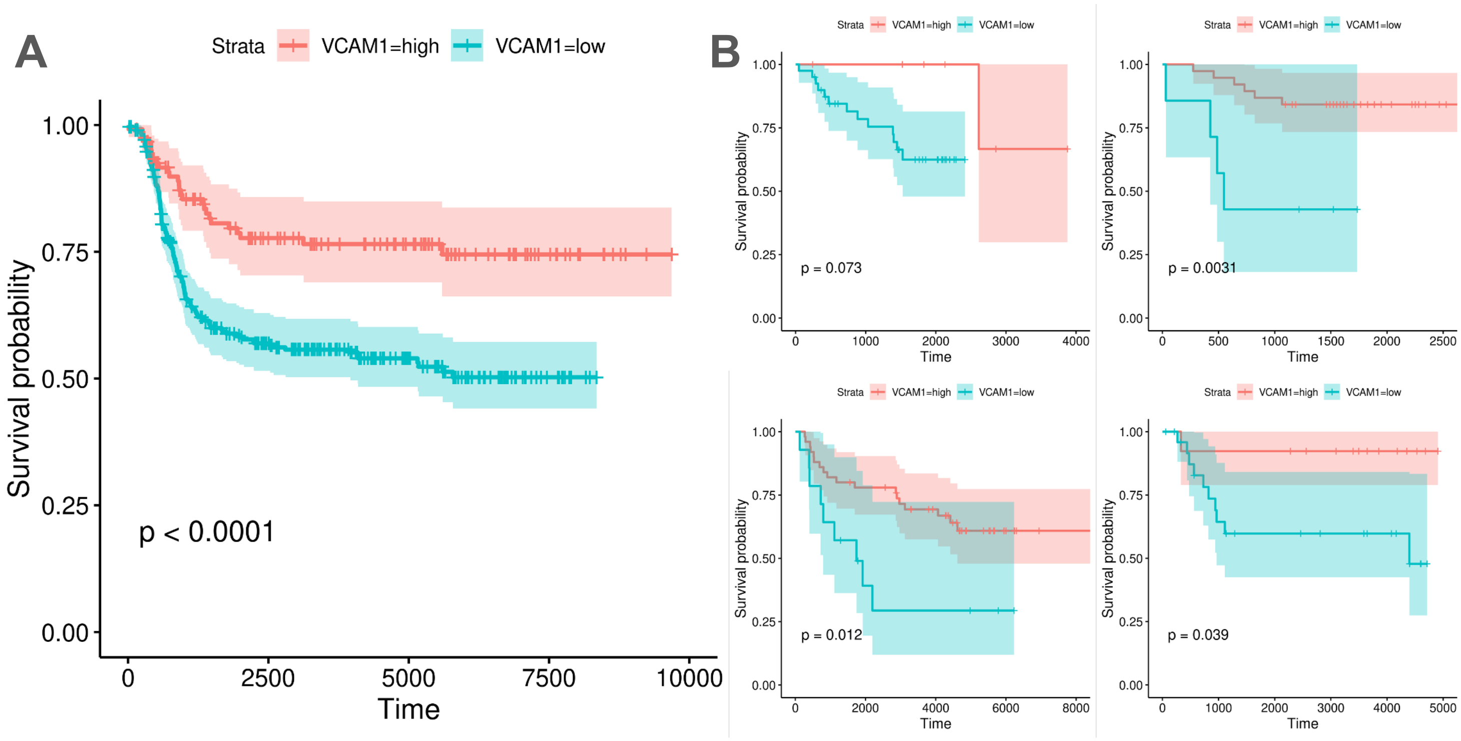

Supplementary Figure S9: Kaplan-Meier plots depicting the relationship between VCAM1 expression and distant relapse in ER- tumors. (**A**) Probability of distant relapse in ER- tumors with high and low VCAM1 expression in the METABRIC dataset. (**B**) Probability of distant relapse-free survival in ER- tumors with high and low VCAM1 expression in external validation cohorts. VCAM1 expression values were thresholded to maximize the log-rank test statistic.

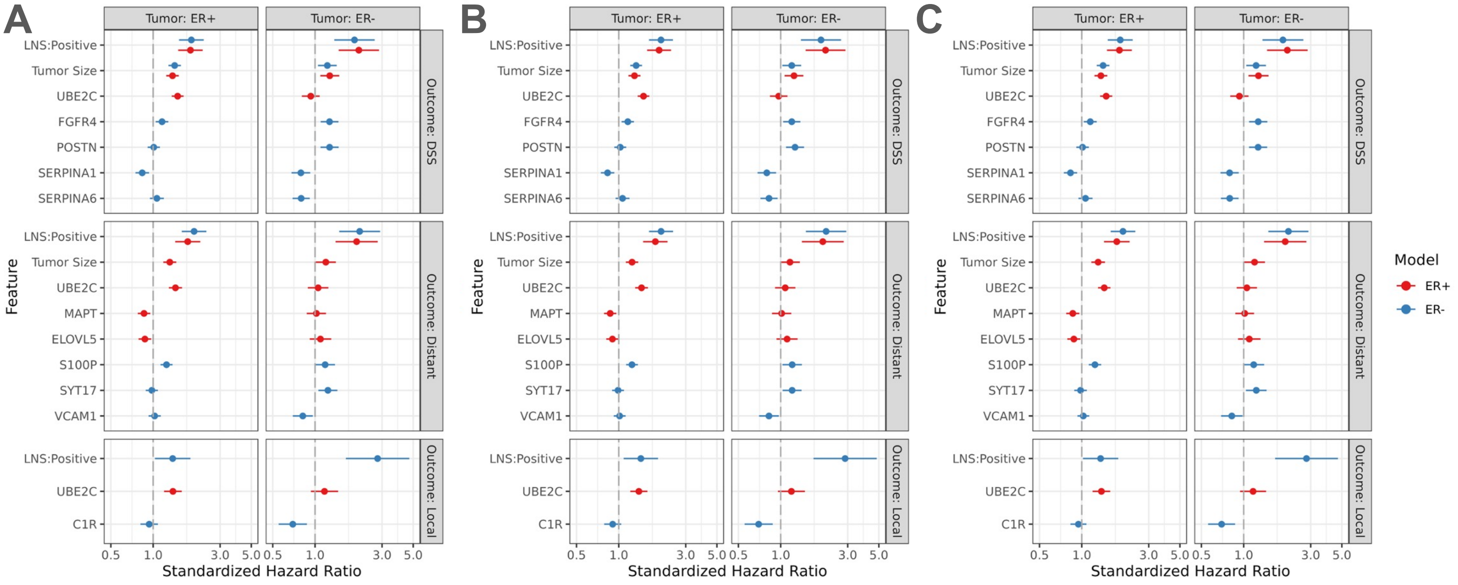

Supplementary Figure S10: Forest plots depicting the standardized hazard ratios (and 95% confidence intervals) from a multivariate Cox regression model for the MB of each mode of breast cancer progression under different strategies for handling missing data. Standardized hazard ratios for features selected by both the ER+ (red) and ER- (blue) models are estimated in ER+ (right) and ER- (left) tumors. (**A**) Standardized hazard ratios estimated using complete cases only (reproduced from **Fig. 5C**). (**B**) Standardized hazard ratios estimated with missing values imputed by missForest. (**C**) Standardized hazard ratios estimated using inverse probability weighting. Effect estimates of the causal factors identified by CausalCoxMGM remain consistent across methods.
