# Supplementary material for: New causal discovery algorithm over censored variables identifies subtype-specific drivers of breast cancer progression
Source: Gigascience. 2026 May 22;15:giag060. doi: 10.1093/gigascience/giag060 (PMC13235964; doi:10.1093/gigascience/giag060)
Supplement: giag060_GIGA-D-25-00149_original_submission [file giag060_giga-d-25-00149_original_submission.pdf]

GigaScience

New causal discovery algorithm over censored variables identifies subtype-specific drivers of breast cancer progression

--Manuscript Draft--

**Manuscript Number:**	GIGA-D-25-00149
**Full Title:**	New causal discovery algorithm over censored variables identifies subtype-specific drivers of breast cancer progression
**Article Type:**	Research
**Funding Information:**	National Heart, Lung, and Blood Institute (R01HL159805) — Prof. Panayiotis V Benos<br>National Heart, Lung, and Blood Institute (R01HL178032) — Prof. Panayiotis V Benos<br>National Institute of Diabetes and Digestive and Kidney Diseases (R01DK130294) — Prof. Panayiotis V Benos<br>U.S. National Library of Medicine (F31LM013966) — Prof. Panayiotis V Benos
**Abstract:**	Many research domains are producing large, multi-scale, multi-modal datasets at growing rates with mixed variable types (continuous, discrete, censored). Identifying possible cause-effect associations in such datasets is essential for predicting outcomes and proposing possible interventions. Probabilistic graphical models (PGMs) have emerged as a robust, interpretable way to analyze such datasets, but current graph learning algorithms cannot incorporate time-to-event (censored) variables, which are important in many systems (e.g., patient survival). Instead, regression models are typically used for survival analysis of single censored variables, but these cannot assess cause-effect interactions. Here, we present a new mathematical framework to incorporate multiple censored variables into mixed graphical models. A novel efficient algorithm, CausalCoxMGM, is implemented, which is extensively evaluated on synthetic and real-life high-dimensional biomedical datasets (cardiovascular disease, breast cancer). CausalCoxMGM was able to recover effectors of censored variables, supported by literature, and provided new mechanistic insights on the differences between ER+ and ER- breast cancers.
**Corresponding Author:**	Panayiotis V Benos, PhD<br>University of Florida<br>Gainesville, Florida UNITED STATES
**Corresponding Author Secondary Information:**	
**Corresponding Author's Institution:**	University of Florida
**Corresponding Author's Secondary Institution:**	
**First Author:**	Panayiotis V Benos, PhD
**First Author Secondary Information:**	
**Order of Authors:**	Panayiotis V Benos, PhD
	Tyler C Lovelace, PhD
**Order of Authors Secondary Information:**	
**Additional Information:**	
**Question**	**Response**
Are you submitting this manuscript to a special series or article collection?	No
**Experimental design and statistics**	Yes

Powered by Editorial Manager® and ProduXion Manager® from Aries Systems Corporation

Full details of the experimental design and statistical methods used should be given in the Methods section, as detailed in our Minimum Standards Reporting Checklist. Information essential to interpreting the data presented should be made available in the figure legends.<br><br>Have you included all the information requested in your manuscript?	
**Resources**<br><br>A description of all resources used, including antibodies, cell lines, animals and software tools, with enough information to allow them to be uniquely identified, should be included in the Methods section. Authors are strongly encouraged to cite Research Resource Identifiers (RRIDs) for antibodies, model organisms and tools, where possible.<br><br>Have you included the information requested as detailed in our Minimum Standards Reporting Checklist?	No
If not, please give reasons for any omissions below.<br><br>as follow-up to "**Resources**<br><br>A description of all resources used, including antibodies, cell lines, animals and software tools, with enough information to allow them to be uniquely identified, should be included in the Methods section. Authors are strongly encouraged to cite Research Resource Identifiers (RRIDs) for antibodies, model organisms and tools, where possible.<br><br>Have you included the information	We did not perform any wet lab experimentation. The software we developed is deposited in GitHub, as we mention in the paper (Section "Data sharing plans").<br><br>The GitHub directory is: https://github.com/tyler-lovelace1/CausalCoxMGM

requested as detailed in our Minimum Standards Reporting Checklist?<br><br>"	
**Availability of data and materials**<br><br><br>All datasets and code on which the conclusions of the paper rely must be either included in your submission or deposited in publicly available repositories (where available and ethically appropriate), referencing such data using a unique identifier in the references and in the “Availability of Data and Materials” section of your manuscript.<br><br><br>Have you have met the above requirement as detailed in our Minimum Standards Reporting Checklist?	Yes
GigaScience has policies and guidelines in place for the use of generative AI-writing tools such as ChatGPT. If you have used such writing tools to assist with writing the manuscript this must be declared and cited in the text. Authors should not list AI-writing tools and other AI-assisted technologies as an author or co-author and should acknowledge that they are fully responsible for text generated or refined by AI-writing tools.<p><br><br>A summary of use (particularly in the introduction or among methods) needs to be included at the end of the paper, and the outputs should also be included as a supplementary file hosted in GigaDB or other open repositories. Please read our guidelines for more information. <p><br><br>By submitting to GigaScience, you are aware of the journal's AI-writing tools policy, and if you have declared use of such tools below, you have acknowledged this where appropriate in your manuscript and have made a summary of use and outputs available. <p><br><b>AI-assisted writing tools have been used in the preparation of this manuscript?	No

Manuscript

Click here to access/download;Manuscript;CausalCoxMGM_manuscr.v20250

New causal discovery algorithm over censored variables identifies subtype-specific drivers of breast cancer progression

Tyler C. Lovelace[1,2], Panayiotis V. Benos[1,2,3]

[1]Department of Computational & Systems Biology, University of Pittsburgh School of Medicine, Pittsburgh, PA, USA

[2]Joint CMU-Pitt PhD Program in Computational Biology, Pittsburgh, PA, USA

[3]Department of Epidemiology, University of Florida, Gainesville, FL, USA

Keywords: mixed graphical models; survival analysis; machine learning

Abstract

Many research domains are producing large, multi-scale, multi-modal datasets at growing rates with mixed variable types (continuous, discrete, censored). Identifying possible cause-effect associations in such datasets is essential for predicting outcomes and proposing possible interventions. Probabilistic graphical models (PGMs) have emerged as a robust, interpretable way to analyze such datasets, but current graph learning algorithms cannot incorporate time-to-event (censored) variables, which are important in many systems (e.g., patient survival). Instead, regression models are typically used for survival analysis of *single* censored variables, but these cannot assess cause-effect interactions. Here, we present a new mathematical framework to incorporate *multiple censored variables* into mixed graphical models. A novel efficient algorithm, *CausalCoxMGM*, is implemented, which is extensively evaluated on synthetic and real-life high-dimensional biomedical datasets (cardiovascular disease, breast cancer). *CausalCoxMGM* was able to recover effectors of censored variables, supported by literature, and provided new mechanistic insights on the differences between ER+ and ER- breast cancers.

Significance statement

Censored (*i.e.*, time-to-event) variables are critically important in many scientific fields, including engineering and medicine. In biomedicine, they represent patients' progression-free time and survival, the time ICU patients remain ventilated to name few examples. Identifying factors that affect these time-dependent patient outcomes can help improve patient care. Classical regression-based analyses cannot identify true causes of censored variables and only operate on one censored variable. Here, we present CausalCoxMGM, the first method that can learn a directed (causal) graphs over multiple censored, continuous, and discrete variables from observational data. We demonstrate its utility across diverse biomedical settings. In breast cancer, we identified estrogen receptor subtype-specific drivers of disease progression, including factors linked to disease-specific survival and time to distant/local relapse.

Introduction

There has been an explosion of data collected in various fields in terms of both type and volume, including from biological and biomedical systems. Mining these complex, multi-modal, and multi-scale datasets for mechanistic insights is essential, especially when it involves time-to-event (censored) variables, like patient survival, time to cancer remission, or a machine's time-to-failure. Machine learning has proven quite efficient for general analysis needs[1]. However, model interpretability remains a challenge. Though they often perform well in classification, they are not designed to identify the complex network of direct (cause-effect) interactions in a dataset, which is critical for gaining mechanistic insights and suggesting interventions to improve outcomes. Probabilistic graphical models (PGMs) have gained popularity in filling this gap. PGMs learn and represent conditional independence relations among all variables in a dataset, and prior work on directed[2,3] and undirected[4,5] PGMs has demonstrated their ability to improve inference through interpretable modeling of biological systems. Additionally, in the last decade, new algorithms have enabled PGMs to be learned on mixed datasets containing continuous and discrete variables[6-10].

However, in clinical settings and other fields, key features of interest are censored (i.e., "time-to-event" features, like survival time, time to cancer remission, etc.). Currently, graph-learning methods cannot incorporate censored variables. We note that a censored variable (partially observed) is different from the standard missing value problem, since censoring provides partial information (*e.g.*, the patient did not die 16 months after the treatment).

We present the mathematical and algorithmic framework for learning mixed graphical models[6-8,] incorporating (multiple) censored variables. We demonstrate its ability to recover adjacencies and edge orientations on synthetic datasets and to recover well-supported associations with censored outcomes in real-world cardiovascular disease datasets.

Additionally, we demonstrate the ability of CausalCoxMGM to mine complex high-dimensional datasets for biological insights through the identification of clinical and gene expression signatures of breast cancer progression in estrogen receptor (ER) positive and negative subtypes. Despite having a common tissue of origin, ER+ and ER- breast cancers are distinct diseases[11] that undergo different pathways of progression[12,13] associated with different biological processes[14].

Methods

Simulated data

We generated 20 Erdős–Rényi (ER; random) and 20 scale-free (SF) directed acyclic graphs (DAGs) each of three different node counts: 55, 110, and 550. Each graph included 5/11 continuous features, 5/11 discrete features, and 1/11 censored features, with an average node degree of 4. Additional ER and SF graphs with 110 nodes were generated to have average node degrees of 2 and 6 to study the effect of graph density.

To assess the effect of sample size on model learning, we simulated datasets of 100, 250, 500, 1000, 5000, and 10000 samples for ER and SF graphs with 110 nodes and an average degree of 4. To assess the effect of censoring rate on graph recovery, each dataset was simulated under light censoring (30% censored; 70% observed) and heavy censoring (70% censored; 30% observed) conditions. For full simulation details, see **Supplementary Methods**.

Biological data

Two cardiovascular disease datasets were used to construct causal models with censored outcomes. The first, *peakVO2*[6], includes 39 clinical, demographic, and exercise stress

test features and all-cause mortality data from individuals with systolic heart failure. The second, *whas500*[8], contains 13 clinical and demographic features recorded from 500 patients hospitalized for acute myocardial infarction (AMI) in the Worcester Heart Attack Study[9]. Two censored outcomes were recorded: time-to-discharge and all-cause mortality, with in-hospital death treated as censoring in the time-to-discharge outcome.

Breast cancer gene expression and clinical data for the METABRIC study[15] were obtained from cBioPortal, while validation cohorts[16-24] were downloaded from GEO (see **Supplementary Methods** for accession numbers). The $\log_2$-transformed METABRIC microarray data were used as provided by cBioPortal. For each microarray validation cohort, raw CEL files were processed with *oligo*[25] to give $\log_2$-transformed data. For RNA-seq data, a variance-stabilizing transform was applied to raw counts with *DESeq2*[26]. ComBat, implemented in *sva*[27], was used to align validation cohorts with METABRIC gene expression data. We selected clinical features linked to breast cancer prognosis and subtype, including age at diagnosis, menopausal state, tumor size, lymph node status, histologic grade, histologic subtype, and receptor statuses (ER, PR, HER2)[11,12]. Samples missing clinical data were excluded. After selecting highly variant genes and filtering to reduce multicollinearity (see **Supplementary Methods**), 437 gene expression features were included. The nonparanormal transform[13] was applied to continuous features to enable CoxMGM and CausalCoxMGM to capture rank-based associations.

The METABRIC dataset records breast cancer progression and mortality as censored time-to-event variables, including disease-specific survival (DSS), death by other causes (OD), distant relapse (DR), and locoregional relapse (LR). We also analyzed common combined metrics: overall survival (OS; DSS and OD), distant relapse-free survival (DRFS; DSS, OD, and DR), and disease-free survival (DFS; DSS, OD, DR, and LR). Due to differing progression pathways and baseline hazards for ER+ and ER- breast cancers, separate causal models were

learned for each subtype. Further details on hyperparameter selection are provided in the **Supplementary Methods**.

Statistical analysis

To evaluate causal graph recovery on simulated networks, we measure adjacency and orientation precision, recall, and F_1 score[28] (**Supplementary Methods; Table S1**). Precision and recall metrics for CoxMGM and CausalCoxMGM are summarized over a range of hyperparameters by the area under the precision-recall curve (AUPRC), while individual model comparisons use F_1 scores. Overall graph recovery is assessed with the Structural Hamming Distance (SHD) from the Markov Equivalence Class (MEC) of the true causal graph. The MEC is a partially directed graph that representing DAGs that yield the same conditional independence relationships (**Supplementary Methods**). The F_1 score is also used to assess feature selection in CausalCoxMGM and LASSO Cox regression.

Predictive models constructed with CausalCoxMGM are Cox regression models regressed on the Markov blanket (MB) of each outcome, where the MB is the set of variables that renders the outcome independent of all other variables when conditioned upon. Predictors of composite outcomes in METABRIC (*e.g.* DRFS) are constructed with a multistate model (**Supplementary Figure S1**) implemented with *mstate*[29]. Baseline models using LASSO Cox regression and random survival forests (RSF) were learned with *glmnet*[30] and *randomForestSRC*[31] respectively, with composite outcomes predicted directly. Predictive accuracy for censored variables is measured with Harrell's concordance[32]. Internal validation uses 10-fold cross-validation, while external validation combines accuracy in individual datasets into a summary statistic for the Meta Cohort using *survcomp*[33].

Results

Overview of the method

CausalCoxMGM (**Figure 1**) provides a framework for learning interpretable causal probabilistic graphical models over heterogenous datasets containing continuous, discrete, and censored variables. This approach enables the integration biomedical data from varying modalities (*e.g.* transcriptomics, metabolomics) with clinical and demographic data and censored outcomes. CausalCoxMGM learns the graphical model through a two-step process: (1) learning an initial estimate of the adjacencies with the undirected graphical model CoxMGM, and (2) pruning spurious adjacencies and orienting edges with constraint-based causal discovery algorithms such as PC or FCI. The resulting causal graphical model not only enables the construction of parsimonious and robust predictors of censored outcomes, but also provides insights for hypothesis generation, risk stratification, and understanding mechanisms driving patient outcomes.

Cox Mixed Graphical Models

The CoxMGM utilizes the Cox proportional hazards model (**Supplementary Methods**) to expand the Mixed Graphical Model[6] (MGM; **Supplementary Methods**) framework to incorporate censored variables along with continuous and discrete data types. Here, we denote continuous and discrete variables with x and y respectively, while censored variables are represented as pairs $\{t, \delta\}$, where t is the event time or last follow-up and δ is the event indicator. This model allows flexible penalization across edge types by assigning different edge potentials: γ for continuous-censored edges and ψ for discrete-censored edges.

Using a second-order approximation of the Cox proportional hazards partial log-likelihood, CoxMGM defines a joint distribution over x, y, and $\{t, \delta\}$ as:

$$P(x,y,t,\delta;\Theta) \propto \exp\left(\sum_{s=1}^{p}\sum_{t=1}^{p} -\frac{1}{2}\beta_{st}x_s x_t + \sum_{s=1}^{p}\alpha_s x_s + \sum_{s=1}^{p}\sum_{j=1}^{q}\rho_{sj}(y_j)x_s + \sum_{j=1}^{q}\sum_{k=1}^{q}\phi_{jk}(y_j,y_k) + \sum_{s=1}^{p}\sum_{m=1}^{r}\gamma_{sm}x_s W_m z_m + \sum_{j=1}^{q}\sum_{m=1}^{r}\psi_{jm}(y_j)W_m z_m\right)$$

where $\widehat{\eta_m} = \widehat{\gamma_m}x + \widehat{\psi_m}(y)$, $z_m = \widehat{\eta_m} - l''(\widehat{\eta_m})^{-1}l'(\widehat{\eta_m})$, and W_m is a diagonal weight matrix such that $\mathrm{diag}(W_m) = \mathrm{diag}\left(l''(\widehat{\eta_m})\right)$. Efron's correction for tied times[35] is used when multiple samples experience events or censoring at the same time.

Node-wise conditional distributions under CoxMGM are modeled by Gaussian linear regressions for continuous variables, multinomial logistic regressions for discrete variables, and Cox regressions for censored variables. This model is fit by minimizing the negative log-pseudolikelihood:

$$\tilde{\mathrm{l}}(\Theta \mid x,y,t,\delta) = -\sum_{s=1}^{p}\log p\left(x_s \mid x_{\backslash s},y,t,\delta;\Theta\right) - \sum_{j=1}^{q}\log p\left(y_j \mid x,y_{\backslash j},t,\delta;\Theta\right) - \sum_{m=1}^{r}\log p\left(T_m,\delta_m \mid x,y;\Theta\right)$$

To flexibly encourage sparsity, CoxMGM applies separate penalties for each edge type: λ_{cc} for continuous-continuous edges, λ_{cd} for continuous-discrete edges, λ_{dd} for discrete-discrete edges, λ_{sc} for continuous-censored edges, and λ_{sd} for discrete-censored edges. The penalized pseudolikelihood is:

$$\min_{\lambda}\tilde{l_\lambda}(\Theta) = \tilde{l}(\Theta) + \lambda_{cc}\sum_{s=1}^{p}\sum_{t=1}^{s-1}|\beta_{st}| + \lambda_{cd}\sum_{s=1}^{p}\sum_{j=1}^{q}\parallel \rho_{sj}\parallel_2 + \lambda_{dd}\sum_{j=1}^{q}\sum_{k=1}^{j-1}\parallel \phi_{jk}\parallel_F + \lambda_{sc}\sum_{s=1}^{p}\sum_{m=1}^{r}|\gamma_{sm}| + \lambda_{sd}\sum_{j=1}^{q}\sum_{m=1}^{r}\parallel \psi_{jm}\parallel_2$$

Each penalty value is selected using the Stable Edge-specific Penalty Selection (StEPS) algorithm[7], optimizing regularization to maintain stability across subsamples. Nonzero regression coefficients indicate edges in the learned graph structure, enabling CoxMGM to model relationships among continuous, discrete, and censored variables.

Independence test for censored variables in mixed datasets

For constraint-based causal discovery on datasets containing censored, continuous, and discrete variables, we need an independence test that accommodates censoring. Expanding on an independence test for mixed datasets (**Supplementary Methods**), our method must test: (1) whether a censored variable X is independent of another variable Y given conditioning set S, and (2) whether X is independent of Y given a conditioning set S that includes censored variables.

The first task can be accomplished using Cox regression as follows:

- When Y is continuous, we use Cox regression to model X with respect to Y and S, then perform a t-test on Y's coefficient to calculate its p-value.
- When Y is discrete, we compare two Cox models of X: one conditioned on S alone (null model) and the other on Y and S. A likelihood ratio test between these two models yields the p-value.

If the p-value is below the threshold α we reject the null hypothesis of conditional independence.

The second task, involving conditioning on censored variables in S, is more complex due to *expansion bias*[36]. Expansion bias occurs because censoring constrains reported values to be less than the true event time, skewing regression coefficients when both uncensored and censored values are included in the analysis. A common but limited workaround is to perform

the regression using only samples where all censored variables in S are observed. While unbiased under uninformative censoring, this approach reduces test power as censorship increases or as more censored variables are included in S.

We propose an alternative approach to conditioning on censored variables that leverages the CoxMGM formulation. Instead of directly using censored values, we condition on censored covariates through the term Wz derived from the second-order approximation of the Cox model. Specifically, for each censored covariate, we use its null model to compute Wz, where Wz reduces to the Martingale residuals of the null model. These residuals measure the difference between the number of observed and expected events up to the observation time[37], approximating the functional associations of censored covariates in conditional independence tests. This approach has a two key benefits: (1) we retain all samples in conditional independence tests and (2) Martingale residuals have been shown to effectively approximate functional relationship between covariates and censored variables in the Cox model[37], making them ideal for representing censored covariates in our test.

Simulated network recovery

To evaluate the performance and limitations of our causal discovery algorithms, we generated synthetic Erdős–Rényi (ER) and scale-free (SF) directed acyclic graphs (DAGs) across a range of different graph sizes, graph degrees, sample sizes, and censorship conditions. As graph recovery depends on hyperparameter selection, we assess the performance of both CoxMGM (undirected graph) and CausalCoxMGM (directed graph) across a range of hyperparameters, summarizing results using AUPRC. Additionally, we demonstrate CausalCoxMGM's effectiveness in feature selection compared to LASSO Cox regression.

Undirected graph recovery with CoxMGM

Figure 2 illustrates CoxMGM's adjacency recovery across a range of sample sizes, graph sizes, and graph degrees, showing that AUPRC approaches 1 with increasing sample sizes for all edges, including those with censored variables (SC, SD), confirming the algorithm's asymptotic correctness. Notably, at a fixed sample size of 500, increasing the number of nodes or graph degree results harms graph recovery, especially in scale-free graphs. As expected, scale-free (SF) graphs are more difficult to learn than Erdős–Rényi (ER) graphs due to the limited number of high-degree nodes, although this difference diminishes as sample size increases.

A key aspect for assessing CoxMGM's performance is the effect of censoring rates on the recovery of the causal skeleton. High censorship (i.e., few observed events) hampers graph recovery across all edge types (ALL) and especially for edges involving censored variables (SC, SD). This effect is most pronounced at low sample sizes, large graphs, high graph degrees, and for edges connecting censored and discrete variables (SD). As expected, the power to recover edges involving censored variables is directly related to the number of observed events, though this limitation decreases as sample size increases.

Finally, stability-based hyperparameter selection strategies for undirected graphical models (StARS, StEPS) perform well compared to an oracle and outperform the BIC score at larger sample sizes (**Supplementary Figure S2A**). At lower sample sizes, the models selected by BIC, StARS, and StEPS have similar F_1 scores across all edge types. However, as sample size increases, StARS and StEPS significantly outperform BIC. Furthermore, in large and high degree graphs StEPS significantly outperforms StARS, particularly in edges between censored and continuous variables (**Supplementary Figure S2BC**).

Causal skeleton recovery with CausalCoxMGM

We assess CausalCoxMGM's ability to recover the adjacencies in the true causal graph after applying MPC-Stable (Majority PC) to the initial skeleton estimated by CoxMGM selected via StEPS. **Figure 3** shows the AUPRC for causal skeleton recovery in the same simulated networks as **Figure 2**, allowing us to evaluate performance across various α values, sample sizes, graph sizes, graph degrees, and censorship rates. The results mirror the trends observed with CoxMGM, with AUPRC asymptotically approaching 1 as sample size increases.

The effect of high graph degree and SF graph architectures is more pronounced in CausalCoxMGM, highlighting a known limitation of constraint-based causal discovery methods. Despite this, precision remains relatively high across all conditions, while the recall increases substantially as sample size increases (**Supplementary Figure S3**). This is especially pronounced in SF networks and in edges involving censored variables. High censoring rates significantly reduce recall in these edges, but this effect diminishes as sample size increases.

Causal orientation recovery with CausalCoxMGM

A key benefit of the CausalCoxMGM algorithm is its ability to infer directionality in the graphical model. Here, we evaluate its performance in recovering the true orientations by assessing the precision and recall of directed edge orientations in the estimated graph compared to the true causal DAG. **Figure 4A** illustrates orientation AUPRC across sample sizes, graph sizes, graph degrees, and censorship rates for the same ER and SF networks as **Figure 2**.

As sample size increases, recovery of causal orientations improves across all conditions, although at a slower rate than adjacency recovery, as orienting edges is more challenging. The orientation AUPRC levels off asymptotically at a value below 1, as constraint-based methods like CausalCoxMGM cannot orient all edges from observational data alone and

instead return the MEC. High censoring rates hinder the recovery of orientations in edges involving censored variables to a greater degree than adjacencies, although the AUPRC still increases asymptotically. Notably, high censoring rates have a greater impact on overall orientation recovery than overall adjacency recovery.

Combined measure of causal graph discovery

Structural Hamming Distance (SHD) is a combined metric of causal graph recovery that integrates adjacency and orientation information. As a distance metric, it enables the principled selection of the best model without the need to balance adjacency and orientation accuracy. **Figure 4B** displays the normalized SHD for graphs learned with $\alpha=0.05$ across various sample sizes, graph sizes, graph degrees, and censorship rates for the same networks as **Figure 2**.

Increasing sample size results in lower normalized SHD across network types, with SHD values for SF graphs consistently higher than ER graphs, reflecting their challenging to recover structure. Additionally, higher censorship rates lead to poorer structure recovery for edges involving censored variables. The normalized SHD remains relatively stable across graph sizes at a fixed sample size, while increasing graph degree hampers graph recovery.

Feature selection performance

CausalCoxMGM not only learns the structure of causal models around censored variables but also serves as a powerful tool for feature selection for downstream prediction tasks. In our simulated networks, CausalCoxMGM more effectively selects parsimonious feature subsets compared to LASSO Cox regression. Specifically, at sample sizes greater than 500, the MB and direct neighbors of censored variables in our causal graphical models recovered the true associations with covariates more accurately, as measured by the F_1 score (**Supplementary Figure S4A**).

The LASSO Cox regression model selected at the minimum deviance from 10-fold cross-validation consistently underperformed, while the model selected by the one standard error rule performed similarly or better than CausalCoxMGM at low sample sizes. Notably, even at low sample sizes, CausalCoxMGM achieved significantly higher precision than LASSO Cox regression using the one standard error rule (**Supplementary Figure S4B**).

Causal discovery in biomedical datasets with censored outcomes

Going beyond simulated data, we applied CausalCoxMGM to real-life biomedical datasets with censored variables, specifically focusing on low-dimensional clinical datasets related to cardiovascular disease. In this context, where associations between clinical features and outcomes are more easily verified against existing literature, we demonstrate that CausalCoxMGM effectively learns causal interactions involving censored variables.

All-cause mortality in systolic heart failure

We applied CausalCoxMGM to identify features affecting all-cause mortality in individuals with systolic heart failure from a dataset containing clinical, demographic, and cardiopulmonary stress testing features[38]. The resulting causal graphical model (**Supplementary Figure S5**) identified seven features directly linked to all-cause mortality. Notably, decreased peak oxygen consumption (Peak VO2) and treadmill exercise duration were linked to increased mortality risk. Additional factors linked to increased mortality risk include lower left ventricular ejection fraction (LVEF), male gender, and elevated blood urea nitrogen (BUN), the latter serving as an indicator of renal function. Furthermore, treatment with beta-blockers reduced mortality risk, while treatment with digoxin increased it.

Mortality and time-to-discharge after hospitalization for acute myocardial infarction

We also applied CausalCoxMGM to a small cohort[39] from the Worcester Heart Attack Study[40], focusing on outcomes for patients hospitalized for acute myocardial infarction (AMI).

This study includes two censored variables: time-to-discharge (where discharge is the event and deaths before discharge are censored) and all-cause mortality after admission. The causal graphical model learned by CausalCoxMGM (**Supplementary Figure S6**) captured expected associations with reasonable causal orientations and directions (positive/negative) of association. Higher BMI was associated with decreased mortality risk after AMI, while low diastolic blood pressure was linked to increased risk. Additionally, elevated heart rate and higher age at admission, both established independent risk factors for in-hospital mortality after an AMI[41], were linked to elevated mortality risk. Finally, two complications, congestive heart failure and cardiogenic shock, were associated with increased mortality risk post-AMI. Utilizing our time-to-discharge data, we found that these two complications, along with atrial fibrillation, contributed to prolonged hospital stays due to the need for additional care and monitoring.

Causal analysis of subtype-specific breast cancer progression

We applied CausalCoxMGM to the METABRIC breast cancer dataset to identify potential causal drivers of DSS, distant relapse, locoregional relapse, and death by other causes amongst gene expression features and clinical covariates. Due to distinct mechanisms of development and progression[12] and dynamics of relapse and survival[13] in ER+ and ER- breast cancers, we learned and compared two separate models (**Figure 5A**). Notably, two clinical features directly describing disease progression, tumor size and lymph node status, are linked to DSS and/or relapse in both subtype models and are established prognostic indicators of survival in breast cancer[42]. As expected, age at diagnosis is the only feature linked to death by other causes.

In contrast, gene expression features linked to breast cancer progression differ between ER+ and ER- subtypes (**Figure 5B**). We assessed the association of these features with DSS, distant relapse and locoregional relapse by learning multivariate Cox proportional hazards models based on each feature's MB in the ER+ and ER- models. In ER+ tumors, high UBE2C

expression was linked to increased disease-specific mortality and relapse, and low expression of MAPT and ELOVL5 are linked to increased distant relapse. In ER- tumors, high expression of FGFR4 and POSTN and low expression of SERPINA1 and SERPINA6 were linked to increased disease-specific mortality. Additionally, elevated S100P and SYT17 and decreased VCAM1 expression are linked to distant relapse, and low C1R expression is associated with locoregional relapse.

We also tested whether gene expression features identified in the causal model of one subtype were significantly associated with progression in the other subtype. For DSS, distant relapse, and locoregional relapse, the ER+ model genes were not significantly associated with progression in ER- tumors. Similarly, most ER- model genes, except for SERPINA1, S100P, and FGFR4, were not significantly associated with progression in ER+ tumors. These results underscore the distinct molecular drivers of progression in ER+ and ER- breast cancer subtypes.

Subtype-specific predictive model of breast cancer progression

Using the subset of features identified by our causal graphs, we constructed subtype-specific predictors of DSS, OD, distant relapse, and locoregional relapse. Composite outcomes such as OS, DRFS, and DFS were modeled using a multi-state Cox model based on these individual outcomes. Internal validation was performed with 10-fold cross-validation, and external validation was conducted through meta-analysis of predictive accuracy in eight external breast cancer cohorts with at least one of DSS, OS, DRFS, and DFS. We compared our models to baseline LASSO Cox regression and random survival forest (RSF) models.

In 10-fold cross-validation, CausalCoxMGM models were able to successfully predict most censored outcomes in breast cancer except for locoregional relapse. In contrast, LASSO Cox regression struggled to learn parsimonious predictors. LASSO Cox regression models

selected with minimum cross-validation deviance (Min) were able to successfully predict censored outcomes other than locoregional relapse but required significantly more features (**Figure 6AB**). The LASSO Cox regression models selected with the one standard error rule (1SE) selected a similar number of features as CausalCoxMGM but exhibited lower predictive accuracy, especially in ER- tumors, where it failed to predict DSS, distant relapse, and locoregional relapse.

To further validate our findings, we applied these models to an external composite validation cohort of eight breast cancer datasets that recorded various measures of breast cancer progression and mortality. Apart from DSS, these cohorts recorded composite measures of breast cancer progression (OS, DRFS, DFS) instead of distinct outcomes. We predicted these outcomes using the multi-state model described above, calculating probabilities for transitions to corresponding states (*e.g.* DSS, OD, and distant relapse for DRFS). LASSO Cox regression and RSF models, trained directly on these composite outcomes, served as baselines.

Figure 6C shows that CausalCoxMGM significantly predicts all four outcomes in the external meta cohort, both overall and in ER+ and ER- tumors individually. CausalCoxMGM performs comparably to LASSO Cox regression and RSF on simpler outcomes (DSS, OS), but outperforms them on more complex outcomes (DRFS, DFS). Additionally, the CausalCoxMGM multi-state model utilizes a smaller, more interpretable set of features than those identified by LASSO Cox regression.

Tumor microenvironment and mortality in ER- tumors

To investigate possible mechanistic roles of CausalCoxMGM prognostic genes, we performed bulk cell type deconvolution[43] of the METABRIC dataset using a single-cell breast cancer atlas as a reference[44]. Through this deconvolution, we analyzed cell-type-specific

expression of the identified prognostic genes (**Supplementary Figure S7**). As expected, across ER+ and ER- tumors, genes associated with breast cancer progression are predominantly expressed by cancer epithelial cells, including UBE2C, MAPT, and ELOVL5 in ER+ tumors and FGFR4, S100P, SERPINA6, and SYT17 in ER- tumors. However, in ER- tumors, certain genes associated with DSS, distant, and locoregional relapse (POSTN, VCAM1, and C1R) were primarily expressed in cancer-associated fibroblasts (CAFs). This observation aligns with POSTN's known roles in promoting metastasis and maintaining stem cell-like properties in breast cancer cells[45,46]. Additionally, SERPINA1, linked to DSS in ER- tumors, was primarily expressed by myeloid cells, suggesting immune involvement.

Discussion

We introduce CausalCoxMGM, a method for causal discovery in datasets with continuous, discrete, and censored variables. CausalCoxMGM combines (1) an undirected CoxMGM that models linear interactions across variable types, and (2) a regression-based conditional independence test enabling constraint-based causal discovery in heterogeneous datasets with censored outcomes. These advancements make CausalCoxMGM a unique tool for causal discovery in datasets with censored outcomes, allowing robust identification of causal relationships and providing novel insights into subtype-specific breast cancer progression.

CausalCoxMGM recovered known associations with mortality in systolic heart failure and AMI patients. In systolic heart failure, it linked mortality to known predictors such as peak VO2[47], treadmill exercise duration[38,48], LVEF[48,49], gender[50], and BUN[48,51], supporting previous findings. Additionally, beta-blockers, recommended as a first-line therapy[52,53], were associated with reduced mortality risk, while digoxin was linked to increased mortality, aligning with a recent retrospective analysis that found similar risks for patients without atrial fibrillation[54]. In individuals

hospitalized with AMI, CausalCoxMGM identified known links with mortality and time-to-discharge, including a well-documented "obesity paradox" where high BMI is linked to lower mortality[55]. It also identified a link between diastolic blood pressure and mortality risk after AMI, despite its U-shaped relationship with mortality[56] violating linearity assumptions in our model. Three complications of AMI (congestive heart failure, cardiogenic shock, and atrial fibrillation) are also linked to mortality, time-to-discharge, or both with expected positive/negative associations.

In our subtype-specific analysis of breast cancer, CausalCoxMGM linked clinical features of well-established significance (Lymph node status and tumor size)[42] and eleven genes with breast cancer progression. In ER+ tumors, CausalCoxMGM identified UBE2C overexpression as a driver of breast cancer progression, a finding supported by mechanistic work that showed UBE2C promotes tumor growth in ER+/HER2- tumors in both an estrogen-dependent and independent manner[57]. UBE2C is a downstream target of ERα-signaling that promotes cell cycle progression and proliferation, and constitutive overexpression promotes cell proliferation even in breast cancer cell lines treated with tamoxifen, an inhibitor of ERα-signaling. Combined with our model of breast cancer progression in ER+ tumors, these results suggest that drugs targeting UBE2C, used alone and in combination with hormone therapies such as tamoxifen, could result in better outcomes for patients with ER+ breast cancer.

In ER- breast cancer, overexpression of FGFR4 is linked to poor disease-specific survival, consistent with experimental work in triple-negative (ER-/PR-/HER2-) breast cancer cell lines showing FGFR4 promotes cell survival through PI3K/AKT activation and FGFR4 knockdown induces cell death[58]. Additionally, they provide evidence for co-expression of FGFR4 and FGF19 and show that inhibition of FGF19 also induces cell death in a dose-dependent manner, suggesting that FGFR4-FGF19 autocrine signaling promotes breast cancer cell survival

in some ER- tumors[58]. Disruption of this signaling pathway could lead to treatments for triple-negative breast cancer, which is particularly aggressive and difficult to treat[59].

Additional genes were linked with distant relapse in breast cancer. In ER+ tumors, low MAPT and ELOVL5 expression has been linked to breast cancer progression[60,61], with experiments in mice showing that ELOVL5 knockdown promotes metastasis[61]. In triple-negative tumors, S100P overexpression has been linked to worse outcomes[62,63], with S100P knockdown inhibiting trans-endothelial migration *in vitro*[62]. Notably, low VCAM1 expression was associated with increased risk of distant relapse in ER- tumors, contradicting prior experimental work showing VCAM1 promotes lung and bone metastasis[64-66]. However, the association of low VCAM1 with distant relapse is strongly significant in ER- tumors in METABRIC, and the trend is maintained in DRFS in ER- tumors in our external validation cohorts (**Supplementary Figure S8**). Our cell-type-specific analysis showed VCAM1 is most highly expressed in CAFs, suggesting VCAM1's role in the tumor microenvironment may differ from its role in metastatic cancer cells, warranting further investigation.

CausalCoxMGM's ability to identify robust, interpretable associations between clinical and gene expression features and distinct modes of breast cancer progression in both ER+ and ER- tumors enabled the construction of a multi-state model of complex outcomes that generalized well to external cohorts. This multi-state model particularly excelled in predicting composite outcomes (DRFS, DFS), outperforming baselines trained directly on these outcomes. CausalCoxMGM's interpretability also facilitated identification of subtype-specific genes linked to progression with potential as prognostic markers or therapeutic targets.

Despite these strengths, CausalCoxMGM has several limitations. Constraint-based causal discovery cannot definitively establish causality in observational data due to assumptions of acyclic cause-effect relationships, Markov faithfulness, and asymptotically large sample sizes. CausalCoxMGM further assumes linear interactions, though the nonparanormal transform can

relax this to additive monotonic interactions. Simulations showed that certain causal structure characteristics (*e.g.* high degree, scale-free networks) can hinder causal structure recovery. Low sample sizes and high censoring rates also harm CausalCoxMGM's reliability in recovering the ground truth. Nevertheless, even under these unfavorable conditions, CausalCoxMGM identifies robust conditional dependencies with high precision, making it a principled tool for hypothesis generation in exploratory analyses.

As the first method capable of causal modeling in heterogeneous datasets with censored outcomes, CausalCoxMGM provides a valuable framework for clinical data analysis. By identifying robust associations and generating clinically relevant hypotheses, this approach will enable future studies to refine treatment strategies, identify novel intervention targets, and provide interpretable prognoses. Furthermore, CausalCoxMGM's flexibility makes it adaptable to other fields that utilize censored data, such as credit risk assessment.

Competing interests. The authors declare no competing interests.

Data availability. We used only publicly vailable datasets and simulated data we generated. The datasets supporting the results of this article are available in the following repositories (see also Supplementary Methods).

PeakVO2 - https://github.com/cran/randomForestSRC/blob/master/data/peakVO2.rda - Used in analysis of mortality in individuals with systolic heart failure.

whas500 - https://github.com/cran/smoothHR/blob/master/data/whas500.rda - Used in analysis of mortality and time to discharge after hospitalization for acute myocardial infarction.

METABRIC - https://www.cbioportal.org/study/summary?id=brca_metabric - Used in analysis of subtype-specific modeling of breast cancer progression.

GSE3494 - https://www.ncbi.nlm.nih.gov/geo/query/acc.cgi?acc=GSE3494 - Used as part of Meta Cohort for external validation for prediction of breast cancer progression.

GSE45255 - https://www.ncbi.nlm.nih.gov/geo/query/acc.cgi?acc=GSE45255 - Used as part of Meta Cohort for external validation for prediction of breast cancer progression.

GSE7390 - https://www.ncbi.nlm.nih.gov/geo/query/acc.cgi?acc=GSE7390 - Used as part of Meta Cohort for external validation for prediction of breast cancer progression.

GSE19615 - https://www.ncbi.nlm.nih.gov/geo/query/acc.cgi?acc=GSE19615 - Used as part of Meta Cohort for external validation for prediction of breast cancer progression.

GSE42568 - https://www.ncbi.nlm.nih.gov/geo/query/acc.cgi?acc=GSE42568 - Used as part of Meta Cohort for external validation for prediction of breast cancer progression.

GSE6532 - https://www.ncbi.nlm.nih.gov/geo/query/acc.cgi?acc=GSE6532 - Used as part of Meta Cohort for external validation for prediction of breast cancer progression.

GSE9195 - https://www.ncbi.nlm.nih.gov/geo/query/acc.cgi?acc=GSE9195 - Used as part of Meta Cohort for external validation for prediction of breast cancer progression.

GSE96058 - https://www.ncbi.nlm.nih.gov/geo/query/acc.cgi?acc=GSE96058 - Used as part of Meta Cohort for external validation for prediction of breast cancer progression.

GSE176078 - https://www.ncbi.nlm.nih.gov/geo/query/acc.cgi?acc=GSE176078 - scRNA-seq breast cancer atlas used for bulk deconvolution of METABRIC data.

Availability of source code. The code is available through GitHub (https://github.com/tyler-lovelace1/CausalCoxMGM).

Funding information. This work was supported by the National Institutes of Health (R01HL159805, R01DK130294, R01HL178032, F31LM013966).

References

1 Yu, M. K. *et al.* Visible Machine Learning for Biomedicine. *Cell* **173**, 1562-1565 (2018). https://doi.org:10.1016/j.cell.2018.05.056

2 Friedman, N. Inferring cellular networks using probabilistic graphical models. *Science* **303**, 799-805 (2004). https://doi.org:10.1126/science.1094068

3 Sachs, K., Perez, O., Pe'er, D., Lauffenburger, D. A. & Nolan, G. P. Causal protein-signaling networks derived from multiparameter single-cell data. *Science* **308**, 523-529 (2005). https://doi.org:10.1126/science.1105809

4 Menendez, P., Kourmpetis, Y. A., ter Braak, C. J. & van Eeuwijk, F. A. Gene regulatory networks from multifactorial perturbations using Graphical Lasso: application to the DREAM4 challenge. *PLoS One* **5**, e14147 (2010). https://doi.org:10.1371/journal.pone.0014147

5 Zuo, Y., Cui, Y., Yu, G., Li, R. & Ressom, H. W. Incorporating prior biological knowledge for network-based differential gene expression analysis using differentially weighted graphical LASSO. *BMC Bioinformatics* **18**, 99 (2017). https://doi.org:10.1186/s12859-017-1515-1

6 Lee, J. D. & Hastie, T. J. Learning the Structure of Mixed Graphical Models. *J. Comput. Graph. Stat.* **24**, 230-253 (2015). https://doi.org:10.1080/10618600.2014.900500

7 Sedgewick, A. J., Shi, I., Donovan, R. M. & Benos, P. V. Learning mixed graphical models with separate sparsity parameters and stability-based model selection. *BMC Bioinformatics* **17 Suppl 5**, 175 (2016). https://doi.org:10.1186/s12859-016-1039-0

8 Sedgewick, A. J. *et al.* Mixed graphical models for integrative causal analysis with application to chronic lung disease diagnosis and prognosis. *Bioinformatics* **35**, 1204-1212 (2019). https://doi.org:10.1093/bioinformatics/bty769

9 Andrews, B., Ramsey, J. & Cooper, G. F. Scoring Bayesian Networks of Mixed Variables. *Int J Data Sci Anal* **6**, 3-18 (2018). https://doi.org:10.1007/s41060-017-0085-7

10 Raghu, V. K. *et al.* Comparison of strategies for scalable causal discovery of latent variable models from mixed data. *Int J Data Sci Anal* **6**, 33-45 (2018). https://doi.org:10.1007/s41060-018-0104-3

11 Sotiriou, C. & Pusztai, L. Gene-expression signatures in breast cancer. *N Engl J Med* **360**, 790-800 (2009). https://doi.org:10.1056/NEJMra0801289

12 Lopez-Garcia, M. A., Geyer, F. C., Lacroix-Triki, M., Marchio, C. & Reis-Filho, J. S. Breast cancer precursors revisited: molecular features and progression pathways. *Histopathology* **57**, 171-192 (2010). https://doi.org:10.1111/j.1365-2559.2010.03568.x

13 Rueda, O. M. *et al.* Dynamics of breast-cancer relapse reveal late-recurring ER-positive genomic subgroups. *Nature* **567**, 399-404 (2019). https://doi.org:10.1038/s41586-019-1007-8

14 Desmedt, C. *et al.* Biological processes associated with breast cancer clinical outcome depend on the molecular subtypes. *Clin Cancer Res* **14**, 5158-5165 (2008). https://doi.org:10.1158/1078-0432.CCR-07-4756

15 Curtis, C. *et al.* The genomic and transcriptomic architecture of 2,000 breast tumours reveals novel subgroups. *Nature* **486**, 346-352 (2012). https://doi.org:10.1038/nature10983

16 Miller, L. D. *et al.* An expression signature for p53 status in human breast cancer predicts mutation status, transcriptional effects, and patient survival. *Proc. Natl. Acad. Sci. U. S. A.* **102**, 13550-13555 (2005). https://doi.org:10.1073/pnas.0506230102

17 Nagalla, S. *et al.* Interactions between immunity, proliferation and molecular subtype in breast cancer prognosis. *Genome Biol.* **14**, R34 (2013). https://doi.org:10.1186/gb-2013-14-4-r34
18 Clarke, C. *et al.* Correlating transcriptional networks to breast cancer survival: a large-scale coexpression analysis. *Carcinogenesis* **34**, 2300-2308 (2013). https://doi.org:10.1093/carcin/bgt208
19 Desmedt, C. *et al.* Strong time dependence of the 76-gene prognostic signature for node-negative breast cancer patients in the TRANSBIG multicenter independent validation series. *Clin Cancer Res* **13**, 3207-3214 (2007). https://doi.org:10.1158/1078-0432.CCR-06-2765
20 Brueffer, C. *et al.* Clinical value of RNA sequencing-based classifiers for prediction of the five conventional breast cancer biomarkers: A report from the population-based multicenter Sweden Cancerome Analysis Network-breast initiative. *JCO Precis. Oncol.* **2** (2018). https://doi.org:10.1200/PO.17.00135
21 Schmidt, M. *et al.* The humoral immune system has a key prognostic impact in node-negative breast cancer. *Cancer Res* **68**, 5405-5413 (2008). https://doi.org:10.1158/0008-5472.CAN-07-5206
22 Li, Y. *et al.* Amplification of LAPTM4B and YWHAZ contributes to chemotherapy resistance and recurrence of breast cancer. *Nat Med* **16**, 214-218 (2010). https://doi.org:10.1038/nm.2090
23 Loi, S. *et al.* Definition of clinically distinct molecular subtypes in estrogen receptor-positive breast carcinomas through genomic grade. *J Clin Oncol* **25**, 1239-1246 (2007). https://doi.org:10.1200/JCO.2006.07.1522
24 Loi, S. *et al.* Predicting prognosis using molecular profiling in estrogen receptor-positive breast cancer treated with tamoxifen. *BMC Genomics* **9**, 239 (2008). https://doi.org:10.1186/1471-2164-9-239
25 Carvalho, B. S. & Irizarry, R. A. A framework for oligonucleotide microarray preprocessing. *Bioinformatics* **26**, 2363-2367 (2010).
26 Love, M. I., Huber, W. & Anders, S. Moderated estimation of fold change and dispersion for RNA-seq data with DESeq2. *Genome Biol* **15**, 550 (2014). https://doi.org:10.1186/s13059-014-0550-8
27 Leek, J. T., Johnson, W. E., Parker, H. S., Jaffe, A. E. & Storey, J. D. The sva package for removing batch effects and other unwanted variation in high-throughput experiments. *Bioinformatics* **28**, 882-883 (2012). https://doi.org:10.1093/bioinformatics/bts034
28 Kummerfeld, E., Williams, L. & Ma, S. Power analysis for causal discovery. *International Journal of Data Science and Analytics* **17**, 289-304 (2023). https://doi.org:10.1007/s41060-023-00399-4
29 de Wreede, L. C., Fiocco, M. & Putter, H. mstate: an R package for the analysis of competing risks and multi-state models. *Journal of statistical software* **38**, 1-30 (2011).
30 Simon, N., Friedman, J., Hastie, T. & Tibshirani, R. Regularization Paths for Cox's Proportional Hazards Model via Coordinate Descent. *J. Stat. Softw.* **39**, 1-13 (2011). https://doi.org:10.18637/jss.v039.i05
31 Ishwaran, H., Kogalur, U. B., Blackstone, E. H. & Lauer, M. S. Random survival forests. *aoas* **2**, 841-860 (2008). https://doi.org:10.1214/08-AOAS169
32 Harrell, F. E., Califf, R. M., Pryor, D. B., Lee, K. L. & Rosati, R. A. Evaluating the yield of medical tests. *Jama* **247**, 2543-2546 (1982).
33 Schröder, M. S., Culhane, A. C., Quackenbush, J. & Haibe-Kains, B. survcomp: an R/Bioconductor package for performance assessment and comparison of survival models. *Bioinformatics* **27**, 3206-3208 (2011). https://doi.org:10.1093/bioinformatics/btr511

34 Cox, D. R. Regression Models and Life-Tables. *Journal of the Royal Statistical Society Series B: Statistical Methodology* **34**, 187-202 (1972). https://doi.org:10.1111/j.2517-6161.1972.tb00899.x
35 Efron, B. The Efficiency of Cox's Likelihood Function for Censored Data. *Journal of the American Statistical Association* **72** (1977). https://doi.org:10.1080/01621459.1977.10480613
36 Rigobon, R. & Stoker, T. M. Bias From Censored Regressors. *Journal of Business & Economic Statistics* **27**, 340-353 (2009). https://doi.org:10.1198/jbes.2009.06119
37 Therneau, T. M., Grambsch, P. M. & Fleming, T. R. Martingale-Based Residuals for Survival Models. *Biometrika* **77** (1990). https://doi.org:10.1093/biomet/77.1.147
38 Hsich, E. *et al.* Importance of treadmill exercise time as an initial prognostic screening tool in patients with systolic left ventricular dysfunction. *Circulation* **119**, 3189-3197 (2009). https://doi.org:10.1161/CIRCULATIONAHA.109.848382
39 Hosmer Jr, D. W., Lemeshow, S. & May, S. *Applied survival analysis: regression modeling of time-to-event data*. Vol. 618 (John Wiley & Sons, 2008).
40 Goldberg, R. J. Recent Changes in Attack and Survival Rates of Acute Myocardial Infarction (1975 Through 1981). *Jama* **255** (1986). https://doi.org:10.1001/jama.1986.03370200076031
41 McNamara, R. L. *et al.* Predicting In-Hospital Mortality in Patients With Acute Myocardial Infarction. *J Am Coll Cardiol* **68**, 626-635 (2016). https://doi.org:10.1016/j.jacc.2016.05.049
42 Galea, M. H., Blamey, R. W., Elston, C. E. & Ellis, I. O. The Nottingham Prognostic Index in primary breast cancer. *Breast Cancer Res. Treat.* **22**, 207-219 (1992). https://doi.org:10.1007/BF01840834
43 Chu, T., Wang, Z., Pe'Er, D. & Danko, C. G. Cell type and gene expression deconvolution with BayesPrism enables Bayesian integrative analysis across bulk and single-cell RNA sequencing in oncology. *Nature Cancer* **3**, 505-517 (2022). https://doi.org:10.1038/s43018-022-00356-3
44 Wu, S. Z. *et al.* A single-cell and spatially resolved atlas of human breast cancers. *Nat Genet* **53**, 1334-1347 (2021). https://doi.org:10.1038/s41588-021-00911-1
45 Cui, D., Huang, Z., Liu, Y. & Ouyang, G. The multifaceted role of periostin in priming the tumor microenvironments for tumor progression. *Cell Mol Life Sci* **74**, 4287-4291 (2017). https://doi.org:10.1007/s00018-017-2646-2
46 Wang, X. *et al.* Periostin contributes to the acquisition of multipotent stem cell-like properties in human mammary epithelial cells and breast cancer cells. *PLoS One* **8**, e72962 (2013). https://doi.org:10.1371/journal.pone.0072962
47 Mancini, D. M. *et al.* Value of peak exercise oxygen consumption for optimal timing of cardiac transplantation in ambulatory patients with heart failure. *Circulation* **83**, 778-786 (1991). https://doi.org:10.1161/01.cir.83.3.778
48 O'Connor, C. M. *et al.* Factors related to morbidity and mortality in patients with chronic heart failure with systolic dysfunction: the HF-ACTION predictive risk score model. *Circ Heart Fail* **5**, 63-71 (2012). https://doi.org:10.1161/CIRCHEARTFAILURE.111.963462
49 McDonagh, T. A. *et al.* 2023 Focused Update of the 2021 ESC Guidelines for the diagnosis and treatment of acute and chronic heart failure. *Eur Heart J* **44**, 3627-3639 (2023). https://doi.org:10.1093/eurheartj/ehad195
50 Shah, R. U., Klein, L. & Lloyd-Jones, D. M. Heart failure in women: epidemiology, biology and treatment. *Womens Health (Lond)* **5**, 517-527 (2009). https://doi.org:10.2217/whe.09.50
51 Duan, S., Li, Y. & Yang, P. Predictive value of blood urea nitrogen in heart failure: a systematic review and meta-analysis. *Front Cardiovasc Med* **10**, 1189884 (2023). https://doi.org:10.3389/fcvm.2023.1189884

52 Hunt, S. A. *et al.* ACC/AHA 2005 Guideline Update for the Diagnosis and Management of Chronic Heart Failure in the Adult—Summary Article. *Circulation* **112**, 1825-1852 (2005). https://doi.org:10.1161/circulationaha.105.167587
53 Masarone, D., Martucci, M. L., Errigo, V. & Pacileo, G. The Use of beta-Blockers in Heart Failure with Reduced Ejection Fraction. *J Cardiovasc Dev Dis* **8** (2021). https://doi.org:10.3390/jcdd8090101
54 Kapelios, C. J. *et al.* Digoxin use in contemporary heart failure with reduced ejection fraction: an analysis from the Swedish Heart Failure Registry. *Eur Heart J Cardiovasc Pharmacother* **8**, 756-767 (2022). https://doi.org:10.1093/ehjcvp/pvab079
55 Wang, L. *et al.* Association of overweight and obesity with patient mortality after acute myocardial infarction: a meta-analysis of prospective studies. *Int J Obes (Lond)* **40**, 220-228 (2016). https://doi.org:10.1038/ijo.2015.176
56 D'Agostino, R. B., Belanger, A. J., Kannel, W. B. & Cruickshank, J. M. Relation of low diastolic blood pressure to coronary heart disease death in presence of myocardial infarction: the Framingham Study. *BMJ* **303**, 385-389 (1991). https://doi.org:10.1136/bmj.303.6799.385
57 Kim, Y. J. *et al.* UBE2C Overexpression Aggravates Patient Outcome by Promoting Estrogen-Dependent/Independent Cell Proliferation in Early Hormone Receptor-Positive and HER2-Negative Breast Cancer. *Front Oncol* **9**, 1574 (2019). https://doi.org:10.3389/fonc.2019.01574
58 Tiong, K. H. *et al.* Fibroblast growth factor receptor 4 (FGFR4) and fibroblast growth factor 19 (FGF19) autocrine enhance breast cancer cells survival. *Oncotarget* **7**, 57633-57650 (2016). https://doi.org:10.18632/oncotarget.9328
59 Bianchini, G., De Angelis, C., Licata, L. & Gianni, L. Treatment landscape of triple-negative breast cancer - expanded options, evolving needs. *Nat Rev Clin Oncol* **19**, 91-113 (2022). https://doi.org:10.1038/s41571-021-00565-2
60 Mihaly, Z. *et al.* A meta-analysis of gene expression-based biomarkers predicting outcome after tamoxifen treatment in breast cancer. *Breast Cancer Res Treat* **140**, 219-232 (2013). https://doi.org:10.1007/s10549-013-2622-y
61 Kieu, T. L. *et al.* Downregulation of Elovl5 promotes breast cancer metastasis through a lipid-droplet accumulation-mediated induction of TGF-beta receptors. *Cell Death Dis* **13**, 758 (2022). https://doi.org:10.1038/s41419-022-05209-6
62 Kikuchi, K. *et al.* S100P and Ezrin promote trans-endothelial migration of triple negative breast cancer cells. *Cell Oncol (Dordr)* **42**, 67-80 (2019). https://doi.org:10.1007/s13402-018-0408-2
63 Maierthaler, M. *et al.* S100P and HYAL2 as prognostic markers for patients with triple-negative breast cancer. *Exp Mol Pathol* **99**, 180-187 (2015). https://doi.org:10.1016/j.yexmp.2015.06.010
64 Chen, Q. & Massague, J. Molecular pathways: VCAM-1 as a potential therapeutic target in metastasis. *Clin Cancer Res* **18**, 5520-5525 (2012). https://doi.org:10.1158/1078-0432.CCR-11-2904
65 Chen, Q., Zhang, X. H. & Massague, J. Macrophage binding to receptor VCAM-1 transmits survival signals in breast cancer cells that invade the lungs. *Cancer Cell* **20**, 538-549 (2011). https://doi.org:10.1016/j.ccr.2011.08.025
66 Lu, X. *et al.* VCAM-1 promotes osteolytic expansion of indolent bone micrometastasis of breast cancer by engaging alpha4beta1-positive osteoclast progenitors. *Cancer Cell* **20**, 701-714 (2011). https://doi.org:10.1016/j.ccr.2011.11.002

Supplementary Figures

Click here to access/download
Supplementary Material
3a_CausalCoxMGM_suppl_figures.20250204.pdf

Click here to access/download
Supplementary Material
3b_CausalCoxMGM_Supplementary_Methods.20250204-pvb.pdf

Cover letter
Click here to access/download;Personal Cover;1_Cover Letter - Lovelace - GigaScience.pdf

UF UNIVERSITY of FLORIDA

2004 Mowry Road
Gainesville, FL 32610

Email: pbenos @ ufl.edu
Tel: +1 352 273-5048
Fax: +1 352 273-5365

Panayiotis V. Benos Ph.D.
William Bushnell Presidential Chaired Professor

College of Public Health & Health Professions,
College of Medicine
Department of Epidemiology

April 20, 2025

Editorial Office
GigaScience
Scott Edmunds, PhD, Editor-in-Chief

Dear Dr. Edmunds and colleagues of the editorial board,

We are excited to submit our manuscript titled "*New causal discovery algorithm over censored variables identifies subtype-specific drivers of breast cancer progression*" for consideration for publication in the journal *GigaScience*. This work fills an important knowledge gap in causal discovery: how to incorporate censored variables in the same causal graph learning framework with continuous and discrete variables.

Censored variables (also known as time-to-event variables) are important for many scientific fields. In biomedical sciences for example, censored variables represent attributes like patient survival, the time a cancer patient remains relapse-free, the time a patient in ICU will stay in the ventilator and many others. Thus, **being able to infer potential direct (causal) effectors of censored variables is of outmost importance**, since such analyses can identify underlying mechanisms and suggest interventions that may improve patients' outcomes. Current methods utilize penalized Cox or other types of regression towards a single censored variable (e.g., see a recent paper in *Nature Machine Intelligence*, DOI: 10.1038/s42256-024-00932-5). However, these methods have three drawbacks: **(1)** only one censored variable can be modeled at a time, **(2)** the identified features are correlates to the censored variable rather than true cause-effect interactions, and **(3)** the models cannot infer other potentially important cause-effect interactions between the non-censored variables that may confound the result. This is known to cause false associations to the target variable, which significantly hinders scientific discoveries.

In this paper, we present a comprehensive mathematical framework to learn both undirected and directed (causal) graphs over a set of continuous, discrete, and (multiple) censored variables using conditional independence tests. We implemented a novel algorithm, *CausalCoxMGM*, to learn such graphs from observational data. Of note, CausalCoxMGM is the first graph algorithm that can analyze datasets with multiple censored variables. We tested CausalMGM extensively on synthetic data at different sample sizes, graph characteristics, and censoring rates to better understand its limits in recovering the true edges and true orientations.

Next, we tested CausalCoxMGM on three real-life datasets: two cardiovascular disease datasets and one high-dimensional breast cancer dataset. In the former datasets we tested its ability to successfully identify known clinical predictors of (a) all-cause mortality in individuals with systolic heart failure, and (b) all-cause mortality and time to discharge in individuals hospitalized after acute myocardial infarction. In the breast cancer dataset, we were interested in identifying and comparing the genes and clinical variables that are differentially affecting the time to one of four events in ER+ and ER- breast cancers. The events are DSS: disease-specific survival; OD: death by other causes; DR: distant relapse; LR: locoregional relapse. Notably, all four outcomes were included in the learned graph together with clinical and gene expression measurements. We identified clinical predictors of progression that are consistent with prior knowledge as well as

novel subtype-specific gene expression signatures of progression. We found that **UBE2C** strongly predicts progression in ER+ tumors, corroborating recent *in vitro* mechanistic studies, but was independent of progression in ER- tumors, suggesting a subtype-dependent mechanism. In ER- tumors, **FGFR4**, a gene being investigated as a possible drug target in solid tumors, is directly linked to DSS, suggesting it as a therapeutic target in triple negative breast cancer, a difficult to treat ER- subtype. Surprisingly, **VCAM1** was protective for distant metastases in ER- tumors, a result supported in our external validation cohorts but contradicted in prior studies in mouse models, suggesting that its role in metastasis is not fully understood.

Finally, since ground truth is not typically available for real-life datasets, we indirectly validated the efficiency of CausalCoxMGM by using the Markov blanket variables to build predictive models of commonly used composite censored outcomes (OS: overall survival; DRFS: distant relapse-free survival; DFS: disease-free survival). We compared CausalCoxMGM to LASSO Cox regression and Random Survival Forests in a meta-analysis of external cohorts. In all cases, CausalCoxMGM concordance at 5 years outcome was the same or better than the other two, with CausalCoxMGM performing particularly well in composite outcomes such as DRFS and DFS.

In summary, our key contributions include:

1. **A novel theoretical framework** for performing conditional independence test on a set of continuous, discrete and (multiple) censored variables.
2. **A novel algorithm** to learn undirected and directed (causal) graphs from observational mixed data (continuous, discrete, censored variables) and build informative predictors of multiple censored variables.
3. **Identification of UBE2C, FGFR4, and VCAM1** as subtype-specific direct effectors of four different outcomes in ER+ and ER- breast cancers (DSS, OD, DR, LR).
4. **Parsimonious predictive model of 4 outcomes** in ER+ and ER- cases.

We'd like to emphasize that **our method is agnostic to the type of data it processes, and thus it can be applied to any dataset that includes censored variables**.

Thank you for considering our manuscript. We are excited with the possibility of sharing our findings with your esteemed journal. Please do not hesitate to contact us if any additional information is needed.

We look forward to hearing back from you in due time.

Yours sincerely,

Panayiotis (Takis) Benos, PhD
William Bushnell Presidential Chaired Professor,
Department of Epidemiology, PHHP and COM, University of Florida
Tel.: 352-273-5048
e-mail: pbenos@ufl.edu
Lab URL: https://benos.epidemiology.phhp.ufl.edu

(on behalf of all authors)
