# Supplementary material for: New causal discovery algorithm over censored variables identifies subtype-specific drivers of breast cancer progression
Source: Gigascience. 2026 May 22;15:giag060. doi: 10.1093/gigascience/giag060 (PMC13235964; doi:10.1093/gigascience/giag060)
Supplement: giag060_GIGA-D-25-00149_revision_1 [file giag060_giga-d-25-00149_revision_1.pdf]

GigaScience

New causal discovery algorithm over censored variables identifies subtype-specific drivers of breast cancer progression

--Manuscript Draft--

**Manuscript Number:**	GIGA-D-25-00149R1
**Full Title:**	New causal discovery algorithm over censored variables identifies subtype-specific drivers of breast cancer progression
**Article Type:**	Research
**Funding Information:**	National Heart, Lung, and Blood Institute (R01HL159805) — Prof. Panayiotis V Benos<br>National Heart, Lung, and Blood Institute (R01HL178032) — Prof. Panayiotis V Benos<br>National Institute of Diabetes and Digestive and Kidney Diseases (R01DK130294) — Prof. Panayiotis V Benos<br>U.S. National Library of Medicine (F31LM013966) — Prof. Panayiotis V Benos
**Abstract:**	Many research domains are producing large, multi-scale, multi-modal datasets at growing rates with mixed variable types (continuous, discrete, censored). Identifying possible cause-effect associations in such datasets is essential for predicting outcomes and proposing possible interventions. Probabilistic graphical models (PGMs) have emerged as a robust, interpretable way to analyze such datasets, but current graph learning algorithms cannot incorporate time-to-event (censored) variables, which are important in many systems (e.g., patient survival). Instead, regression models are typically used for survival analysis of single censored variables, but these cannot assess cause-effect interactions. Here, we present a new mathematical framework to incorporate multiple censored variables into mixed graphical models. A novel efficient algorithm, CausalCoxMGM, is implemented, which is extensively evaluated on synthetic and real-life high-dimensional biomedical datasets (cardiovascular disease, breast cancer). CausalCoxMGM was able to recover effectors of censored variables, supported by literature, and provided new mechanistic insights on the differences between ER+ and ER- breast cancers.
**Corresponding Author:**	Panayiotis V Benos, PhD<br>University of Florida<br>Gainesville, Florida UNITED STATES
**Corresponding Author Secondary Information:**	
**Corresponding Author's Institution:**	University of Florida
**Corresponding Author's Secondary Institution:**	
**First Author:**	Panayiotis V Benos, PhD
**First Author Secondary Information:**	
**Order of Authors:**	Panayiotis V Benos, PhD<br>Tyler C Lovelace, PhD
**Order of Authors Secondary Information:**	
**Response to Reviewers:**	Please see included file with detailed point-by-point responses
**Additional Information:**	
**Question**	**Response**
Are you submitting this manuscript to a special series or article collection?	No

Powered by Editorial Manager® and ProduXion Manager® from Aries Systems Corporation

**Experimental design and statistics**<br><br>Full details of the experimental design and statistical methods used should be given in the Methods section, as detailed in our Minimum Standards Reporting Checklist. Information essential to interpreting the data presented should be made available in the figure legends.<br><br>Have you included all the information requested in your manuscript?	Yes
**Resources**<br><br>A description of all resources used, including antibodies, cell lines, animals and software tools, with enough information to allow them to be uniquely identified, should be included in the Methods section. Authors are strongly encouraged to cite Research Resource Identifiers (RRIDs) for antibodies, model organisms and tools, where possible.<br><br>Have you included the information requested as detailed in our Minimum Standards Reporting Checklist?	No
If not, please give reasons for any omissions below.<br><br>as follow-up to "**Resources**<br><br>A description of all resources used, including antibodies, cell lines, animals and software tools, with enough information to allow them to be uniquely identified, should be included in the Methods section. Authors are strongly encouraged to cite Research Resource Identifiers (RRIDs) for antibodies, model organisms and tools, where possible.	We did not perform any wet lab experimentation. The software we developed is deposited in GitHub, as we mention in the paper (Section "Data sharing plans").<br><br>The GitHub directory is: https://github.com/tyler-lovelace1/CausalCoxMGM

Powered by Editorial Manager® and ProduXion Manager® from Aries Systems Corporation

Have you included the information requested as detailed in our Minimum Standards Reporting Checklist?<br><br>"	
**Availability of data and materials**<br><br>All datasets and code on which the conclusions of the paper rely must be either included in your submission or deposited in publicly available repositories (where available and ethically appropriate), referencing such data using a unique identifier in the references and in the "Availability of Data and Materials" section of your manuscript.<br><br>Have you have met the above requirement as detailed in our Minimum Standards Reporting Checklist?	Yes
GigaScience has policies and guidelines in place for the use of generative AI-writing tools such as ChatGPT. If you have used such writing tools to assist with writing the manuscript this must be declared and cited in the text. Authors should not list AI-writing tools and other AI-assisted technologies as an author or co-author and should acknowledge that they are fully responsible for text generated or refined by AI-writing tools.<p><br><br>A summary of use (particularly in the introduction or among methods) needs to be included at the end of the paper, and the outputs should also be included as a supplementary file hosted in GigaDB or other open repositories. Please read our guidelines for more information. <p><br><br>By submitting to GigaScience, you are aware of the journal's AI-writing tools policy, and if you have declared use of such tools below, you have acknowledged this where appropriate in your manuscript and have made a summary of use and outputs available. <p><br><b>AI-assisted writing tools have been used in the preparation of this manuscript?	No

Manuscript

Click here to access/download;Manuscript;3b_CausalCoxMGM_manuscr.final

New causal discovery algorithm over censored variables identifies subtype-specific drivers of breast cancer progression

Tyler C. Lovelace[1,2], Panayiotis V. Benos[1,2,3]

[1]Department of Computational & Systems Biology, University of Pittsburgh School of Medicine, Pittsburgh, PA, USA

[2]Joint CMU-Pitt PhD Program in Computational Biology, Pittsburgh, PA, USA

[3]Department of Epidemiology, University of Florida, Gainesville, FL, USA

Keywords: mixed graphical models; survival analysis; machine learning

Abstract

Many research domains are producing large, multi-scale, multi-modal datasets at growing rates with mixed variable types (continuous, discrete, censored). Identifying possible cause-effect associations in such datasets is essential for predicting outcomes and proposing possible interventions. Probabilistic graphical models (PGMs) have emerged as a robust, interpretable way to analyze such datasets, but current graph learning algorithms cannot incorporate time-to-event (censored) variables, which are important in many systems (e.g., patient survival). Instead, regression models are typically used for survival analysis of *single* censored variables, but these cannot assess cause-effect interactions. Here, we present a new mathematical framework to incorporate *multiple censored variables* into mixed graphical models. A novel efficient algorithm, *CausalCoxMGM*, is implemented, which is extensively evaluated on synthetic and real-life high-dimensional biomedical datasets (cardiovascular disease, breast cancer). *CausalCoxMGM* was able to recover effectors of censored variables, supported by literature, and provided new mechanistic insights on the differences between ER+ and ER- breast cancers.

Significance statement

Censored (*i.e.*, time-to-event) variables are critically important in many scientific fields, including engineering and medicine. In biomedicine, they represent patients' progression-free time and survival, the time ICU patients remain ventilated to name few examples. Identifying factors that affect these time-dependent patient outcomes can help improve patient care. Classical regression-based analyses cannot identify true causes of censored variables and only operate on one censored variable. Here, we present CausalCoxMGM, the first method that can learn a directed (causal) graphs over multiple censored, continuous, and discrete variables from observational data. We demonstrate its utility across diverse biomedical settings. In breast cancer, we identified estrogen receptor subtype-specific drivers of disease progression, including factors linked to disease-specific survival and time to distant/local relapse.

Introduction

There has been an explosion of data collected in various fields in terms of both type and volume, including from biological and biomedical systems. Mining these complex, multi-modal, and multi-scale datasets for mechanistic insights is essential, especially when it involves time-to-event (censored) variables, like patient survival, time to cancer remission, or a machine's time-to-failure. Machine learning has proven quite efficient for general analysis needs[1]. However, model interpretability remains a challenge. Though they often perform well in classification, they are not designed to identify the complex network of direct (cause-effect) interactions in a dataset, which is critical for gaining mechanistic insights and suggesting interventions to improve outcomes. Probabilistic graphical models (PGMs) have gained popularity in filling this gap. PGMs learn and represent conditional independence relations among all variables in a dataset, and prior work on directed[2, 3] and undirected[4, 5] PGMs has demonstrated their ability to improve inference through interpretable modeling of biological systems. Additionally, in the last decade, new algorithms have enabled PGMs to be learned on mixed datasets containing continuous and discrete variables[6-10].

However, in clinical settings and other fields, key features of interest are censored (i.e., "time-to-event" features, like survival time, time to cancer remission, etc.). Currently, graph-learning methods cannot incorporate censored variables. We note that a censored variable (partially observed) is different from the standard missing value problem, since censoring provides partial information (*e.g.*, the patient did <u>not</u> die 16 months after the treatment).

We present the mathematical and algorithmic framework for learning mixed graphical models[6-8], incorporating (multiple) censored variables. We demonstrate its ability to recover adjacencies and edge orientations on synthetic datasets and to recover well-supported associations with censored outcomes in real-world cardiovascular disease datasets.

Additionally, we demonstrate the ability of CausalCoxMGM to mine complex high-dimensional datasets for biological insights through the identification of clinical and gene expression signatures of breast cancer progression in estrogen receptor (ER) positive and negative subtypes. Despite having a common tissue of origin, ER+ and ER- breast cancers are distinct diseases[11] that undergo different pathways of progression[12, 13] associated with different biological processes[14].

Methods

Simulated data

We generated 20 Erdős–Rényi (ER; random) and 20 scale-free (SF) directed acyclic graphs (DAGs) each of three different node counts: 55, 110, and 550. Each graph included 5/11 continuous features, 5/11 discrete features, and 1/11 censored features, with an average node degree of 4. Additional ER and SF graphs with 110 nodes were generated to have average node degrees of 2 and 6 to study the effect of graph density.

To assess the effect of sample size on model learning, we simulated datasets of 100, 250, 500, 1000, 5000, and 10000 samples for ER and SF graphs with 110 nodes and an average degree of 4. To assess the effect of censoring rate on graph recovery, each dataset was simulated under light censoring (30% censored; 70% observed) and heavy censoring (70% censored; 30% observed) conditions. For full simulation details, see **Supplementary Methods**.

Biological data

Two cardiovascular disease datasets were used to construct causal models with censored outcomes. The first, *peakVO2*[6], includes 39 clinical, demographic, and exercise stress

test features and all-cause mortality data from individuals with systolic heart failure. The second, *whas500*[8], contains 13 clinical and demographic features recorded from 500 patients hospitalized for acute myocardial infarction (AMI) in the Worcester Heart Attack Study[9]. Two censored outcomes were recorded: time-to-discharge and all-cause mortality, with in-hospital death treated as censoring in the time-to-discharge outcome.

Breast cancer gene expression and clinical data for the METABRIC study[15] were obtained from cBioPortal, while validation cohorts[16-24] were downloaded from GEO (see **Supplementary Methods** for accession numbers). The $\log_2$-transformed METABRIC microarray data were used as provided by cBioPortal. For each microarray validation cohort, raw CEL files were processed with *oligo[25]* to give $\log_2$-transformed data. For RNA-seq data, a variance-stabilizing transform was applied to raw counts with *DESeq2[26]*. ComBat*,* implemented in *sva[27],* was used to align validation cohorts with METABRIC gene expression data. We selected clinical features linked to breast cancer prognosis and subtype, including age at diagnosis, menopausal state, tumor size, lymph node status, histologic grade, histologic subtype, and receptor statuses (ER, PR, HER2)[11,12]. Samples missing clinical data were excluded. After selecting highly variant genes and filtering to reduce multicollinearity (see **Supplementary Methods**), 437 gene expression features were included. The nonparanormal transform[13] was applied to continuous features to enable CoxMGM and CausalCoxMGM to capture rank-based associations.

The METABRIC dataset records breast cancer progression and mortality as censored time-to-event variables, including disease-specific survival (DSS), death by other causes (OD), distant relapse (DR), and locoregional relapse (LR). We also analyzed common combined metrics: overall survival (OS; DSS and OD), distant relapse-free survival (DRFS; DSS, OD, and DR), and disease-free survival (DFS; DSS, OD, DR, and LR). Due to differing progression pathways and baseline hazards for ER+ and ER- breast cancers, separate causal models were

learned for each subtype. Further details on hyperparameter selection are provided in the **Supplementary Methods**.

Statistical analysis

To evaluate causal graph recovery on simulated networks, we measure adjacency and orientation precision, recall, and F_1 score[28] (**Supplementary Methods; Table S1**). Precision and recall metrics for CoxMGM and CausalCoxMGM are summarized over a range of hyperparameters by the area under the precision-recall curve (AUPRC), while individual model comparisons use F_1 scores. Overall graph recovery is assessed with the Structural Hamming Distance (SHD) from the Markov Equivalence Class (MEC) of the true causal graph. The MEC is a partially directed graph that representing DAGs that yield the same conditional independence relationships (**Supplementary Methods**). The F_1 score is also used to assess feature selection in CausalCoxMGM and LASSO Cox regression.

Predictive models constructed with CausalCoxMGM are Cox regression models regressed on the Markov blanket (MB) of each outcome, where the MB is the set of variables that renders the outcome independent of all other variables when conditioned upon. Predictors of composite outcomes in METABRIC (*e.g.* DRFS) are constructed with a multistate model (**Supplementary Figure S1**) implemented with *mstate[29]*. Baseline models using LASSO Cox regression and random survival forests (RSF) were learned with *glmnet*[30] and *randomForestSRC*[31] respectively, with composite outcomes predicted directly. Predictive accuracy for censored variables is measured with Harrell's concordance[32]. Internal validation uses 10-fold cross-validation, while external validation combines accuracy in individual datasets into a summary statistic for the Meta Cohort using *survcomp[33]*.

Results

Overview of the method

CausalCoxMGM (**Figure 1**) provides a framework for learning interpretable causal probabilistic graphical models over heterogenous datasets containing continuous, discrete, and censored variables. This approach enables the integration biomedical data from varying modalities (*e.g.* transcriptomics, metabolomics) with clinical and demographic data and censored outcomes. CausalCoxMGM learns the graphical model through a two-step process: (1) learning an initial estimate of the adjacencies with the undirected graphical model CoxMGM, and (2) pruning spurious adjacencies and orienting edges with constraint-based causal discovery algorithms such as PC or FCI. The resulting causal graphical model not only enables the construction of parsimonious and robust predictors of censored outcomes, but also provides insights for hypothesis generation, risk stratification, and understanding mechanisms driving patient outcomes.

Cox Mixed Graphical Models

The CoxMGM utilizes the Cox proportional hazards model (**Supplementary Methods**) to expand the Mixed Graphical Model[6] (MGM; **Supplementary Methods**) framework to incorporate censored variables along with continuous and discrete data types. Here, we denote continuous and discrete variables with x and y respectively, while censored variables are represented as pairs $\{t, \delta\}$, where t is the event time or last follow-up and δ is the event indicator. This model allows flexible penalization across edge types by assigning different edge potentials: γ for continuous-censored edges and ψ for discrete-censored edges.

Using a second-order approximation of the Cox proportional hazards partial log-likelihood, CoxMGM defines a joint distribution over x, y, and $\{t, \delta\}$ as:

$$P(x, y, t, \delta; \Theta) \propto \exp\left(\sum_{s=1}^{p}\sum_{t=1}^{p} -\frac{1}{2}\beta_{st}x_s x_t + \sum_{s=1}^{p}\alpha_s x_s + \sum_{s=1}^{p}\sum_{j=1}^{q}\rho_{sj}(y_j)x_s + \sum_{j=1}^{q}\sum_{k=1}^{q}\phi_{jk}(y_j, y_k)\right.$$

$$\left. + \sum_{s=1}^{p}\sum_{m=1}^{r}\gamma_{sm}x_s W_m z_m + \sum_{j=1}^{q}\sum_{m=1}^{r}\psi_{jm}(y_j)W_m z_m\right)$$

where $\widehat{\eta_m} = \widehat{\gamma_m}x + \widehat{\psi_m}(y)$, $z_m = \widehat{\eta_m} - l''(\widehat{\eta_m})^{-1}l'(\widehat{\eta_m})$, and W_m is a diagonal weight matrix such that $\mathrm{diag}(W_m) = \mathrm{diag}\big(l''(\widehat{\eta_m})\big)$. Efron's correction for tied times[34] is used when multiple samples experience events or censoring at the same time.

Node-wise conditional distributions under CoxMGM are modeled by Gaussian linear regressions for continuous variables, multinomial logistic regressions for discrete variables, and Cox regressions for censored variables. This model is fit by minimizing the negative log-pseudolikelihood:

$$\tilde{l}(\Theta \mid x, y, t, \delta) = -\sum_{s=1}^{p}\log p\left(x_s \mid x_{\backslash s}, y, t, \delta; \Theta\right) - \sum_{j=1}^{q}\log p\left(y_j \mid x, y_{\backslash j}, t, \delta; \Theta\right)$$

$$-\sum_{m=1}^{r}\log p\left(T_m, \delta_m \mid x, y; \Theta\right)$$

To flexibly encourage sparsity, CoxMGM applies separate penalties for each edge type: λ_{cc} for continuous-continuous edges, λ_{cd} for continuous-discrete edges, λ_{dd} for discrete-discrete edges, λ_{sc} for continuous-censored edges, and λ_{sd} for discrete-censored edges. The penalized pseudolikelihood is:

$$\min_{\lambda}\widetilde{l_\lambda}(\Theta) = \tilde{l}(\Theta) + \lambda_{cc}\sum_{s=1}^{p}\sum_{t=1}^{s-1}|\beta_{st}| + \lambda_{cd}\sum_{s=1}^{p}\sum_{j=1}^{q}\|\rho_{sj}\|_2 + \lambda_{dd}\sum_{j=1}^{q}\sum_{k=1}^{j-1}\|\phi_{jk}\|_F + \lambda_{sc}\sum_{s=1}^{p}\sum_{m=1}^{r}|\gamma_{sm}|$$

$$+ \lambda_{sd}\sum_{j=1}^{q}\sum_{m=1}^{r}\|\psi_{jm}\|_2$$

Each penalty value is selected using the Stable Edge-specific Penalty Selection (StEPS) algorithm[7], optimizing regularization to maintain stability across subsamples. Nonzero regression coefficients indicate edges in the learned graph structure, enabling CoxMGM to model relationships among continuous, discrete, and censored variables.

Independence test for censored variables in mixed datasets

For constraint-based causal discovery on datasets containing censored, continuous, and discrete variables, we need an independence test that accommodates censoring. Expanding on an independence test for mixed datasets (**Supplementary Methods**), our method must test: (1) whether a censored variable X is independent of another variable Y given conditioning set S, and (2) whether X is independent of Y given a conditioning set S that includes censored variables.

The first task can be accomplished using Cox regression as follows:

- When Y is continuous, we use Cox regression to model X with respect to Y and S, then perform a t-test on Y's coefficient to calculate its p-value.
- When Y is discrete, we compare two Cox models of X: one conditioned on S alone (null model) and the other on Y and S. A likelihood ratio test between these two models yields the p-value.

If the p-value is below the threshold α we reject the null hypothesis of conditional independence.

The second task, involving conditioning on censored variables in S, is more complex due to *expansion bias*[35]. Expansion bias occurs because censoring constrains reported values to be less than the true event time, skewing regression coefficients when both uncensored and censored values are included in the analysis. A common but limited workaround is to perform

the regression using only samples where all censored variables in S are observed. While unbiased under uninformative censoring, this approach reduces test power as censorship increases or as more censored variables are included in S.

We propose an alternative approach to conditioning on censored variables that leverages the CoxMGM formulation. Instead of directly using censored values, we condition on censored covariates through the term Wz derived from the second-order approximation of the Cox model. Specifically, for each censored covariate, we use its null model to compute Wz, where Wz reduces to the Martingale residuals of the null model. These residuals measure the difference between the number of observed and expected events up to the observation time[36], approximating the functional associations of censored covariates in conditional independence tests. This approach has a two key benefits: (1) we retain all samples in conditional independence tests and (2) Martingale residuals have been shown to effectively approximate functional relationship between covariates and censored variables in the Cox model[36], making them ideal for representing censored covariates in our test.

Simulated network recovery

To evaluate the performance and limitations of our causal discovery algorithms, we generated synthetic Erdős–Rényi (ER) and scale-free (SF) directed acyclic graphs (DAGs) across a range of different graph sizes, graph degrees, sample sizes, and censorship conditions. As graph recovery depends on hyperparameter selection, we assess the performance of both CoxMGM (undirected graph) and CausalCoxMGM (directed graph) across a range of hyperparameters, summarizing results using AUPRC. Additionally, we demonstrate CausalCoxMGM's effectiveness in feature selection compared to LASSO Cox regression.

Undirected graph recovery with CoxMGM

Figure 2 illustrates CoxMGM's adjacency recovery across a range of sample sizes, graph sizes, and graph degrees, showing that AUPRC approaches 1 with increasing sample sizes for all edges, including those with censored variables (SC, SD), confirming the algorithm's asymptotic correctness. Notably, at a fixed sample size of 500, increasing the number of nodes or graph degree results harms graph recovery, especially in scale-free graphs. As expected, scale-free (SF) graphs are more difficult to learn than Erdős–Rényi (ER) graphs due to the limited number of high-degree nodes, although this difference diminishes as sample size increases.

A key aspect for assessing CoxMGM's performance is the effect of censoring rates on the recovery of the causal skeleton. High censorship (i.e., few observed events) hampers graph recovery across all edge types (ALL) and especially for edges involving censored variables (SC, SD). This effect is most pronounced at low sample sizes, large graphs, high graph degrees, and for edges connecting censored and discrete variables (SD). As expected, the power to recover edges involving censored variables is directly related to the number of observed events, though this limitation decreases as sample size increases.

Finally, stability-based hyperparameter selection strategies for undirected graphical models (StARS, StEPS) perform well compared to an oracle and outperform the BIC score at larger sample sizes (**Supplementary Figure S2A**). At lower sample sizes, the models selected by BIC, StARS, and StEPS have similar F_1 scores across all edge types. However, as sample size increases, StARS and StEPS significantly outperform BIC. Furthermore, in large and high degree graphs StEPS significantly outperforms StARS, particularly in edges between censored and continuous variables (**Supplementary Figure S2BC**).

Causal skeleton recovery with CausalCoxMGM

We assess CausalCoxMGM's ability to recover the adjacencies in the true causal graph after applying MPC-Stable (Majority PC) to the initial skeleton estimated by CoxMGM selected via StEPS. **Figure 3** shows the AUPRC for causal skeleton recovery in the same simulated networks as **Figure 2**, allowing us to evaluate performance across various α values, sample sizes, graph sizes, graph degrees, and censorship rates. The results mirror the trends observed with CoxMGM, with AUPRC asymptotically approaching 1 as sample size increases. As expected for causal discovery algorithms, low sample sizes (<250 samples) significantly decreases the adjacency AUPRC. However, we note that even at low sample sizes (n = 100) it is possible to recover adjacencies with high precision (*i.e.* with a low false discovery rate; **Supplementary Figure S3**).

The effect of high graph degree and SF graph architectures is more pronounced in CausalCoxMGM, highlighting a known limitation of constraint-based causal discovery methods. Despite this, precision remains relatively high across all conditions, while the recall increases substantially as sample size increases (**Supplementary Figure S3**). This is especially pronounced in SF networks and in edges involving censored variables. High censoring rates significantly reduce recall in these edges, but this effect diminishes as sample size increases.

Causal orientation recovery with CausalCoxMGM

A key benefit of the CausalCoxMGM algorithm is its ability to infer directionality in the graphical model. Here, we evaluate its performance in recovering the true orientations by assessing the precision and recall of directed edge orientations in the estimated graph compared to the true causal DAG. **Figure 4A** illustrates orientation AUPRC across sample sizes, graph sizes, graph degrees, and censorship rates for the same ER and SF networks as **Figure 2**.

As sample size increases, recovery of causal orientations again improves across all conditions. However, this occurs at a slower rate than adjacency recovery, as orienting edges is more challenging. This suggests that at lower sample sizes ($n<500$), adjacency recovery is more reliable than orientation recovery. The orientation AUPRC also levels off asymptotically at a value below 1, as constraint-based methods like CausalCoxMGM cannot orient all edges from observational data alone and instead return the MEC. High censoring rates hinder the recovery of orientations in edges involving censored variables to a greater degree than adjacencies, although the AUPRC still increases asymptotically. Notably, high censoring rates have a greater impact on overall orientation recovery than overall adjacency recovery.

Combined measure of causal graph discovery

Structural Hamming Distance (SHD) is a combined metric of causal graph recovery that integrates adjacency and orientation information. As a distance metric, it enables the principled selection of the best model without the need to balance adjacency and orientation accuracy. **Figure 4B** displays the normalized SHD for graphs learned with $\alpha=0.05$ across various sample sizes, graph sizes, graph degrees, and censorship rates for the same networks as **Figure 2**.

Increasing sample size results in lower normalized SHD across network types, with SHD values for SF graphs consistently higher than ER graphs, reflecting their challenging to recover structure. Additionally, higher censorship rates lead to poorer structure recovery for edges involving censored variables. The normalized SHD remains relatively stable across graph sizes at a fixed sample size, while increasing graph degree hampers graph recovery.

Feature selection performance

CausalCoxMGM not only learns the structure of causal models around censored variables but also serves as a powerful tool for feature selection for downstream prediction tasks. In our simulated networks, CausalCoxMGM more effectively selects parsimonious feature

subsets compared to LASSO Cox regression. Specifically, at sample sizes greater than 500, the MB and direct neighbors of censored variables in our causal graphical models recovered the true associations with covariates more accurately, as measured by the F_1 score (**Supplementary Figure S4A**).

The LASSO Cox regression model selected at the minimum deviance from 10-fold cross-validation consistently underperformed, while the model selected by the one standard error rule performed similarly or better than CausalCoxMGM at low sample sizes. Notably, even at low sample sizes, CausalCoxMGM achieved significantly higher precision than LASSO Cox regression using the one standard error rule (**Supplementary Figure S4B**).

Causal discovery in biomedical datasets with censored outcomes

Going beyond simulated data, we applied CausalCoxMGM to real-life biomedical datasets with censored variables, specifically focusing on low-dimensional clinical datasets related to cardiovascular disease. In this context, where associations between clinical features and outcomes are more easily verified against existing literature, we demonstrate that CausalCoxMGM effectively learns causal interactions involving censored variables.

All-cause mortality in systolic heart failure

We applied CausalCoxMGM to identify features affecting all-cause mortality in individuals with systolic heart failure from a dataset containing clinical, demographic, and cardiopulmonary stress testing features[37]. The resulting causal graphical model (**Supplementary Figure S5**) identified seven features directly linked to all-cause mortality. Notably, decreased peak oxygen consumption (Peak VO2) and treadmill exercise duration were linked to increased mortality risk. Additional factors linked to increased mortality risk include lower left ventricular ejection fraction (LVEF), male gender, and elevated blood urea nitrogen

(BUN), the latter serving as an indicator of renal function. Furthermore, treatment with beta-blockers reduced mortality risk, while treatment with digoxin increased it.

Mortality and time-to-discharge after hospitalization for acute myocardial infarction

We also applied CausalCoxMGM to a small cohort[38] from the Worcester Heart Attack Study[39], focusing on outcomes for patients hospitalized for acute myocardial infarction (AMI). This study includes two censored variables: time-to-discharge (where discharge is the event and deaths before discharge are censored) and all-cause mortality after admission. The causal graphical model learned by CausalCoxMGM (**Supplementary Figure S6**) captured expected associations with reasonable causal orientations and directions (positive/negative) of association. Higher BMI was associated with decreased mortality risk after AMI, while low diastolic blood pressure was linked to increased risk. Additionally, elevated heart rate and higher age at admission, both established independent risk factors for in-hospital mortality after an AMI[40], were linked to elevated mortality risk. Finally, two complications, congestive heart failure and cardiogenic shock, were associated with increased mortality risk post-AMI. Utilizing our time-to-discharge data, we found that these two complications, along with atrial fibrillation, contributed to prolonged hospital stays due to the need for additional care and monitoring.

Causal analysis of subtype-specific breast cancer progression

We applied CausalCoxMGM to the METABRIC breast cancer dataset to identify potential causal drivers of DSS, distant relapse, locoregional relapse, and death by other causes amongst gene expression features and clinical covariates. Due to distinct mechanisms of development and progression[12] and dynamics of relapse and survival[13] in ER+ and ER- breast cancers, we learned and compared two separate models (**Figure 5A**). Notably, two clinical features directly describing disease progression, tumor size and lymph node status, are linked to DSS and/or relapse in both subtype models and are established prognostic indicators

of survival in breast cancer[41]. As expected, age at diagnosis is the only feature linked to death by other causes.

In contrast, gene expression features linked to breast cancer progression differ between ER+ and ER- subtypes (**Figure 5B**)**.** We assessed the association of these features with DSS, distant relapse and locoregional relapse by learning multivariate Cox proportional hazards models based on each feature's MB in the ER+ and ER- models. In ER+ tumors, high UBE2C expression was linked to increased disease-specific mortality and relapse, and low expression of MAPT and ELOVL5 are linked to increased distant relapse. In ER- tumors, high expression of FGFR4 and POSTN and low expression of SERPINA1 and SERPINA6 were linked to increased disease-specific mortality. Additionally, elevated S100P and SYT17 and decreased VCAM1 expression are linked to distant relapse, and low C1R expression is associated with locoregional relapse.

We also tested whether gene expression features identified in the causal model of one subtype were significantly associated with progression in the other subtype. For DSS, distant relapse, and locoregional relapse, the ER+ model genes were not significantly associated with progression in ER- tumors. Similarly, most ER- model genes, except for SERPINA1, S100P, and FGFR4, were not significantly associated with progression in ER+ tumors. These results underscore the distinct molecular drivers of progression in ER+ and ER- breast cancer subtypes.

Subtype-specific predictive model of breast cancer progression

Using the subset of features identified by our causal graphs, we constructed subtype-specific predictors of DSS, OD, distant relapse, and locoregional relapse. Composite outcomes such as OS, DRFS, and DFS were modeled using a multi-state Cox model based on these individual outcomes. Internal validation was performed with 10-fold cross-validation, and

external validation was conducted through meta-analysis of predictive accuracy in eight external breast cancer cohorts with at least one of DSS, OS, DRFS, and DFS. We compared our models to baseline LASSO Cox regression and random survival forest (RSF) models.

In 10-fold cross-validation, CausalCoxMGM models were able to successfully predict most censored outcomes in breast cancer except for locoregional relapse. In contrast, LASSO Cox regression struggled to learn parsimonious predictors. LASSO Cox regression models selected with minimum cross-validation deviance (Min) were able to successfully predict censored outcomes other than locoregional relapse but required significantly more features (**Figure 6AB**). The LASSO Cox regression models selected with the one standard error rule (1SE) selected a similar number of features as CausalCoxMGM but exhibited lower predictive accuracy, especially in ER- tumors, where it failed to predict DSS, distant relapse, and locoregional relapse.

To further validate our findings, we applied these models to an external composite validation cohort of eight breast cancer datasets that recorded various measures of breast cancer progression and mortality. Apart from DSS, these cohorts recorded composite measures of breast cancer progression (OS, DRFS, DFS) instead of distinct outcomes. We predicted these outcomes using the multi-state model described above, calculating probabilities for transitions to corresponding states (*e.g.* DSS, OD, and distant relapse for DRFS). LASSO Cox regression and RSF models, trained directly on these composite outcomes, served as baselines.

Figure 6C shows that CausalCoxMGM significantly predicts all four outcomes in the external meta cohort, both overall and in ER+ and ER- tumors individually. CausalCoxMGM performs comparably to LASSO Cox regression and RSF on simpler outcomes (DSS, OS), but outperforms them on more complex outcomes (DRFS, DFS). Additionally, the CausalCoxMGM

multi-state model utilizes a smaller, more interpretable set of features than those identified by LASSO Cox regression.

An analysis of the calibration, measured by the 5-year Brier score (**Supplementary Figure S7AB**), showed that CausalCoxMGM and the baseline models were similarly calibrated in the METABRIC dataset. In contrast, none of the models achieved consistent calibration in the external validation cohorts, resulting in wide bootstrapped confidence intervals. In conjunction with our model's strong discrimination accuracy, this lack of calibration likely reflects shifts in baseline hazards between the older METABRIC dataset, with tumors collected prior to the development of HER2-targeted therapies, and the more contemporary external validation cohorts. In the SCAN-B cohort (GSE96058; **Supplementary Figure S7C**), for example, our CausalCoxMGM model consistently overestimated mortality, especially in ER- tumors.

Tumor microenvironment and mortality in ER- tumors

To investigate possible mechanistic roles of CausalCoxMGM prognostic genes, we performed bulk cell type deconvolution[42] of the METABRIC dataset using a single-cell breast cancer atlas as a reference[43]. Through this deconvolution, we analyzed cell-type-specific expression of the identified prognostic genes (**Supplementary Figure S8**). As expected, across ER+ and ER- tumors, genes associated with breast cancer progression are predominantly expressed by cancer epithelial cells, including UBE2C, MAPT, and ELOVL5 in ER+ tumors and FGFR4, S100P, SERPINA6, and SYT17 in ER- tumors. However, in ER- tumors, certain genes associated with DSS, distant, and locoregional relapse (POSTN, VCAM1, and C1R) were primarily expressed in cancer-associated fibroblasts (CAFs). This observation aligns with POSTN's known roles in promoting metastasis and maintaining stem cell-like properties in breast cancer cells[44, 45]. Additionally, SERPINA1, linked to DSS in ER- tumors, was primarily expressed by myeloid cells, suggesting immune involvement.

Discussion

We introduce CausalCoxMGM, a method for causal discovery in datasets with continuous, discrete, and censored variables. CausalCoxMGM combines (1) an undirected CoxMGM that models linear interactions across variable types, and (2) a regression-based conditional independence test enabling constraint-based causal discovery in heterogeneous datasets with censored outcomes. These advancements make CausalCoxMGM a unique tool for causal discovery in datasets with censored outcomes, allowing robust identification of causal relationships and providing novel insights into subtype-specific breast cancer progression.

CausalCoxMGM recovered known associations with mortality in systolic heart failure and AMI patients. In systolic heart failure, it linked mortality to known predictors such as peak VO2[46], treadmill exercise duration[37, 47], LVEF[47, 48], gender[49], and BUN[47, 50], supporting previous findings. Additionally, beta-blockers, recommended as a first-line therapy[51, 52], were associated with reduced mortality risk, while digoxin was linked to increased mortality, aligning with a recent retrospective analysis that found similar risks for patients without atrial fibrillation[53]. In individuals hospitalized with AMI, CausalCoxMGM identified known links with mortality and time-to-discharge, including a well-documented "obesity paradox" where high BMI is linked to lower mortality[54]. It also identified a link between diastolic blood pressure and mortality risk after AMI, despite its U-shaped relationship with mortality[55] violating linearity assumptions in our model. Three complications of AMI (congestive heart failure, cardiogenic shock, and atrial fibrillation) are also linked to mortality, time-to-discharge, or both with expected positive/negative associations.

In our subtype-specific analysis of breast cancer, CausalCoxMGM linked clinical features of well-established significance (Lymph node status and tumor size)[41] and eleven genes with

breast cancer progression. In ER+ tumors, CausalCoxMGM identified UBE2C overexpression as a driver of breast cancer progression, a finding supported by mechanistic work that showed UBE2C promotes tumor growth in ER+/HER2- tumors in both an estrogen-dependent and independent manner[56]. UBE2C is a downstream target of ERα-signaling that promotes cell cycle progression and proliferation, and constitutive overexpression promotes cell proliferation even in breast cancer cell lines treated with tamoxifen, an inhibitor of ERα-signaling. Combined with our model of breast cancer progression in ER+ tumors, these results suggest that drugs targeting UBE2C, used alone and in combination with hormone therapies such as tamoxifen, could result in better outcomes for patients with ER+ breast cancer.

In ER- breast cancer, overexpression of FGFR4 is linked to poor disease-specific survival, consistent with experimental work in triple-negative (ER-/PR-/HER2-) breast cancer cell lines showing FGFR4 promotes cell survival through PI3K/AKT activation and FGFR4 knockdown induces cell death[57]. Additionally, they provide evidence for co-expression of FGFR4 and FGF19 and show that inhibition of FGF19 also induces cell death in a dose-dependent manner, suggesting that FGFR4-FGF19 autocrine signaling promotes breast cancer cell survival in some ER- tumors[57]. Disruption of this signaling pathway could lead to treatments for triple-negative breast cancer, which is particularly aggressive and difficult to treat[58].

Additional genes were linked with distant relapse in breast cancer. In ER+ tumors, low MAPT and ELOVL5 expression has been linked to breast cancer progression[59, 60], with experiments in mice showing that ELOVL5 knockdown promotes metastasis[60]. In triple-negative tumors, S100P overexpression has been linked to worse outcomes[61, 62], with S100P knockdown inhibiting trans-endothelial migration *in vitro*[61]. Notably, low VCAM1 expression was associated with increased risk of distant relapse in ER- tumors, contradicting prior experimental work showing VCAM1 promotes lung and bone metastasis[63-65]. However,

the association of low VCAM1 with distant relapse is strongly significant in ER- tumors in METABRIC, and the trend is maintained in DRFS in ER- tumors in our external validation cohorts (**Supplementary Figure S9**). Our cell-type-specific analysis showed VCAM1 is most highly expressed in CAFs, suggesting VCAM1's role in the tumor microenvironment may differ from its role in metastatic cancer cells, warranting further investigation.

CausalCoxMGM's ability to identify robust, interpretable associations between clinical and gene expression features and distinct modes of breast cancer progression in both ER+ and ER- tumors enabled the construction of a multi-state model of complex outcomes that generalized well to external cohorts. This multi-state model particularly excelled in predicting composite outcomes (DRFS, DFS), outperforming baselines trained directly on these outcomes. CausalCoxMGM's interpretability also facilitated identification of subtype-specific genes linked to progression with potential as prognostic markers or therapeutic targets.

Despite these strengths, CausalCoxMGM has several limitations. Constraint-based causal discovery cannot definitively establish causality in observational data due to assumptions of acyclic cause-effect relationships, Markov faithfulness, and asymptotically large sample sizes. CausalCoxMGM further assumes linear interactions, though the nonparanormal transform can relax this to additive monotonic interactions. Simulations showed that certain causal structure characteristics (*e.g.* high degree, scale-free networks) can hinder causal structure recovery. Low sample sizes and high censoring rates also harm CausalCoxMGM's reliability in recovering the ground truth. With fewer than 250 samples or rare events, graph recovery, especially edge orientation, becomes unreliable. Nevertheless, even under these unfavorable conditions, CausalCoxMGM identifies robust conditional dependencies with high precision (*i.e.* low false discovery rate), making it a principled tool for hypothesis generation in exploratory analyses. To assess the stability and significance of recovered adjacencies and edge orientations, strategies such as bootstrapping can be employed.

Our analysis of breast cancer progression likewise has limitations affecting generalizability. Because it is based on observational data, we cannot definitively establish causal relationships for the clinical and gene expression signatures linked to breast cancer progression. Additionally, our analysis of the METABRIC dataset was limited to complete cases, which may introduce selection bias. While the FCI causal discovery algorithm is asymptotically consistent even in the presence of latent confounders and selection bias, complete-case analysis can still bias the estimates of effect sizes. To mitigate this, we re-estimated our predictive model using multiple imputation and inverse probability weighting (**Supplementary Figure S10**) and found no substantial deviations from our complete-case analysis. Finally, although our predictive model demonstrated strong discrimination across diverse external validation cohorts, its calibration was poor, likely reflecting shifts in baseline hazards between the older METABRIC dataset and more recent external validation cohorts. This suggests that recalibration on a contemporary cohort would be necessary before it could be widely applied.

As the first method capable of causal modeling in heterogeneous datasets with censored outcomes, CausalCoxMGM provides a valuable framework for clinical data analysis. By identifying robust associations and generating clinically relevant hypotheses, this approach will enable future studies to refine treatment strategies, identify novel intervention targets, and provide interpretable prognoses. Furthermore, CausalCoxMGM's flexibility makes it adaptable to other fields that utilize censored data, such as credit risk assessment.

Competing interests. The authors declare no competing interests.

Availability of Source Code and Requirements

Project name: CausalCoxMGM

Project homepage: https://github.com/tyler-lovelace1/CausalCoxMGM

Operating system: Platform independent

Programming language: R

Other requirements: R (>=4.4.0)

License: GPL-3.0 license

RRID: SCR_026906

Data availability. We used only publicly available datasets and simulated data we generated. The datasets supporting the results of this article are available in the following repositories (see also **Supplementary Methods**). The rCausalMGM package is also available on CRAN [66]. All additional supporting data [67] is available in the *GigaScience* repository, GigaDB [68].

Funding information. This work was supported by the National Institutes of Health (R01HL159805, R01DK130294, R01HL178032, F31LM013966).

References

1. Yu MK, Ma J, Fisher J, Kreisberg JF, Raphael BJ and Ideker T. Visible Machine Learning for Biomedicine. Cell. 2018;173 7:1562-5. doi:10.1016/j.cell.2018.05.056.
2. Friedman N. Inferring cellular networks using probabilistic graphical models. Science. 2004;303 5659:799-805. doi:10.1126/science.1094068.
3. Sachs K, Perez O, Pe'er D, Lauffenburger DA and Nolan GP. Causal protein-signaling networks derived from multiparameter single-cell data. Science. 2005;308 5721:523-9. doi:10.1126/science.1105809.
4. Menendez P, Kourmpetis YA, ter Braak CJ and van Eeuwijk FA. Gene regulatory networks from multifactorial perturbations using Graphical Lasso: application to the DREAM4 challenge. PLoS One. 2010;5 12:e14147. doi:10.1371/journal.pone.0014147.
5. Zuo Y, Cui Y, Yu G, Li R and Ressom HW. Incorporating prior biological knowledge for network-based differential gene expression analysis using differentially weighted graphical LASSO. BMC Bioinformatics. 2017;18 1:99. doi:10.1186/s12859-017-1515-1.
6. Lee JD and Hastie TJ. Learning the Structure of Mixed Graphical Models. J Comput Graph Stat. 2015;24 1:230-53. doi:10.1080/10618600.2014.900500.
7. Sedgewick AJ, Shi I, Donovan RM and Benos PV. Learning mixed graphical models with separate sparsity parameters and stability-based model selection. BMC Bioinformatics. 2016;17 Suppl 5:175. doi:10.1186/s12859-016-1039-0.
8. Sedgewick AJ, Buschur K, Shi I, Ramsey JD, Raghu VK, Manatakis DV, et al. Mixed graphical models for integrative causal analysis with application to chronic lung disease diagnosis and prognosis. Bioinformatics. 2019;35 7:1204-12. doi:10.1093/bioinformatics/bty769.
9. Andrews B, Ramsey J and Cooper GF. Scoring Bayesian Networks of Mixed Variables. Int J Data Sci Anal. 2018;6 1:3-18. doi:10.1007/s41060-017-0085-7.
10. Raghu VK, Ramsey JD, Morris A, Manatakis DV, Sprites P, Chrysanthis PK, et al. Comparison of strategies for scalable causal discovery of latent variable models from mixed data. Int J Data Sci Anal. 2018;6 1:33-45. doi:10.1007/s41060-018-0104-3.
11. Sotiriou C and Pusztai L. Gene-expression signatures in breast cancer. N Engl J Med. 2009;360 8:790-800. doi:10.1056/NEJMra0801289.
12. Lopez-Garcia MA, Geyer FC, Lacroix-Triki M, Marchio C and Reis-Filho JS. Breast cancer precursors revisited: molecular features and progression pathways. Histopathology. 2010;57 2:171-92. doi:10.1111/j.1365-2559.2010.03568.x.
13. Rueda OM, Sammut SJ, Seoane JA, Chin SF, Caswell-Jin JL, Callari M, et al. Dynamics of breast-cancer relapse reveal late-recurring ER-positive genomic subgroups. Nature. 2019;567 7748:399-404. doi:10.1038/s41586-019-1007-8.
14. Desmedt C, Haibe-Kains B, Wirapati P, Buyse M, Larsimont D, Bontempi G, et al. Biological processes associated with breast cancer clinical outcome depend on the molecular subtypes. Clin Cancer Res. 2008;14 16:5158-65. doi:10.1158/1078-0432.CCR-07-4756.
15. Curtis C, Shah SP, Chin S-F, Turashvili G, Rueda OM, Dunning MJ, et al. The genomic and transcriptomic architecture of 2,000 breast tumours reveals novel subgroups. Nature. 2012;486 7403:346-52. doi:10.1038/nature10983.
16. Miller LD, Smeds J, George J, Vega VB, Vergara L, Ploner A, et al. An expression signature for p53 status in human breast cancer predicts mutation status, transcriptional effects, and patient survival. Proc Natl Acad Sci U S A. 2005;102 38:13550-5. doi:10.1073/pnas.0506230102.

17. Nagalla S, Chou JW, Willingham MC, Ruiz J, Vaughn JP, Dubey P, et al. Interactions between immunity, proliferation and molecular subtype in breast cancer prognosis. Genome Biol. 2013;14 4:R34. doi:10.1186/gb-2013-14-4-r34.
18. Clarke C, Madden SF, Doolan P, Aherne ST, Joyce H, O'Driscoll L, et al. Correlating transcriptional networks to breast cancer survival: a large-scale coexpression analysis. Carcinogenesis. 2013;34 10:2300-8. doi:10.1093/carcin/bgt208.
19. Desmedt C, Piette F, Loi S, Wang Y, Lallemand F, Haibe-Kains B, et al. Strong time dependence of the 76-gene prognostic signature for node-negative breast cancer patients in the TRANSBIG multicenter independent validation series. Clin Cancer Res. 2007;13 11:3207-14. doi:10.1158/1078-0432.CCR-06-2765.
20. Brueffer C, Vallon-Christersson J, Grabau D, Ehinger A, Häkkinen J, Hegardt C, et al. Clinical value of RNA sequencing-based classifiers for prediction of the five conventional breast cancer biomarkers: A report from the population-based multicenter Sweden Cancerome Analysis Network-breast initiative. JCO Precis Oncol. 2018;2 doi:10.1200/PO.17.00135.
21. Schmidt M, Bohm D, von Torne C, Steiner E, Puhl A, Pilch H, et al. The humoral immune system has a key prognostic impact in node-negative breast cancer. Cancer Res. 2008;68 13:5405-13. doi:10.1158/0008-5472.CAN-07-5206.
22. Li Y, Zou L, Li Q, Haibe-Kains B, Tian R, Li Y, et al. Amplification of LAPTM4B and YWHAZ contributes to chemotherapy resistance and recurrence of breast cancer. Nat Med. 2010;16 2:214-8. doi:10.1038/nm.2090.
23. Loi S, Haibe-Kains B, Desmedt C, Lallemand F, Tutt AM, Gillet C, et al. Definition of clinically distinct molecular subtypes in estrogen receptor-positive breast carcinomas through genomic grade. J Clin Oncol. 2007;25 10:1239-46. doi:10.1200/JCO.2006.07.1522.
24. Loi S, Haibe-Kains B, Desmedt C, Wirapati P, Lallemand F, Tutt AM, et al. Predicting prognosis using molecular profiling in estrogen receptor-positive breast cancer treated with tamoxifen. BMC Genomics. 2008;9:239. doi:10.1186/1471-2164-9-239.
25. Carvalho BS and Irizarry RA. A framework for oligonucleotide microarray preprocessing. Bioinformatics. 2010;26 19:2363-7.
26. Love MI, Huber W and Anders S. Moderated estimation of fold change and dispersion for RNA-seq data with DESeq2. Genome Biol. 2014;15 12:550. doi:10.1186/s13059-014-0550-8.
27. Leek JT, Johnson WE, Parker HS, Jaffe AE and Storey JD. The sva package for removing batch effects and other unwanted variation in high-throughput experiments. Bioinformatics. 2012;28 6:882-3. doi:10.1093/bioinformatics/bts034.
28. Kummerfeld E, Williams L and Ma S. Power analysis for causal discovery. International Journal of Data Science and Analytics. 2023;17 3:289-304. doi:10.1007/s41060-023-00399-4.
29. de Wreede LC, Fiocco M and Putter H. mstate: an R package for the analysis of competing risks and multi-state models. Journal of statistical software. 2011;38:1-30.
30. Simon N, Friedman J, Hastie T and Tibshirani R. Regularization Paths for Cox's Proportional Hazards Model via Coordinate Descent. J Stat Softw. 2011;39 5:1-13. doi:10.18637/jss.v039.i05.
31. Ishwaran H, Kogalur UB, Blackstone EH and Lauer MS. Random survival forests. aoas. 2008;2 3:841-60. doi:10.1214/08-AOAS169.
32. Harrell FE, Califf RM, Pryor DB, Lee KL and Rosati RA. Evaluating the yield of medical tests. Jama. 1982;247 18:2543-6.
33. Schröder MS, Culhane AC, Quackenbush J and Haibe-Kains B. survcomp: an R/Bioconductor package for performance assessment and comparison of survival models. Bioinformatics. 2011;27 22:3206-8. doi:10.1093/bioinformatics/btr511.

34. Efron B. The Efficiency of Cox's Likelihood Function for Censored Data. Journal of the American Statistical Association. 1977;72 359 doi:10.1080/01621459.1977.10480613.
35. Rigobon R and Stoker TM. Bias From Censored Regressors. Journal of Business & Economic Statistics. 2009;27 3:340-53. doi:10.1198/jbes.2009.06119.
36. Therneau TM, Grambsch PM and Fleming TR. Martingale-Based Residuals for Survival Models. Biometrika. 1990;77 1 doi:10.1093/biomet/77.1.147.
37. Hsich E, Gorodeski EZ, Starling RC, Blackstone EH, Ishwaran H and Lauer MS. Importance of treadmill exercise time as an initial prognostic screening tool in patients with systolic left ventricular dysfunction. Circulation. 2009;119 25:3189-97. doi:10.1161/CIRCULATIONAHA.109.848382.
38. Hosmer Jr DW, Lemeshow S and May S. Applied survival analysis: regression modeling of time-to-event data. John Wiley & Sons; 2008.
39. Goldberg RJ. Recent Changes in Attack and Survival Rates of Acute Myocardial Infarction (1975 Through 1981). Jama. 1986;255 20 doi:10.1001/jama.1986.03370200076031.
40. McNamara RL, Kennedy KF, Cohen DJ, Diercks DB, Moscucci M, Ramee S, et al. Predicting In-Hospital Mortality in Patients With Acute Myocardial Infarction. J Am Coll Cardiol. 2016;68 6:626-35. doi:10.1016/j.jacc.2016.05.049.
41. Galea MH, Blamey RW, Elston CE and Ellis IO. The Nottingham Prognostic Index in primary breast cancer. Breast Cancer Res Treat. 1992;22 3:207-19. doi:10.1007/BF01840834.
42. Chu T, Wang Z, Pe’Er D and Danko CG. Cell type and gene expression deconvolution with BayesPrism enables Bayesian integrative analysis across bulk and single-cell RNA sequencing in oncology. Nature Cancer. 2022;3 4:505-17. doi:10.1038/s43018-022-00356-3.
43. Wu SZ, Al-Eryani G, Roden DL, Junankar S, Harvey K, Andersson A, et al. A single-cell and spatially resolved atlas of human breast cancers. Nat Genet. 2021;53 9:1334-47. doi:10.1038/s41588-021-00911-1.
44. Cui D, Huang Z, Liu Y and Ouyang G. The multifaceted role of periostin in priming the tumor microenvironments for tumor progression. Cell Mol Life Sci. 2017;74 23:4287-91. doi:10.1007/s00018-017-2646-2.
45. Wang X, Liu J, Wang Z, Huang Y, Liu W, Zhu X, et al. Periostin contributes to the acquisition of multipotent stem cell-like properties in human mammary epithelial cells and breast cancer cells. PLoS One. 2013;8 8:e72962. doi:10.1371/journal.pone.0072962.
46. Mancini DM, Eisen H, Kussmaul W, Mull R, Edmunds LH, Jr. and Wilson JR. Value of peak exercise oxygen consumption for optimal timing of cardiac transplantation in ambulatory patients with heart failure. Circulation. 1991;83 3:778-86. doi:10.1161/01.cir.83.3.778.
47. O'Connor CM, Whellan DJ, Wojdyla D, Leifer E, Clare RM, Ellis SJ, et al. Factors related to morbidity and mortality in patients with chronic heart failure with systolic dysfunction: the HF-ACTION predictive risk score model. Circ Heart Fail. 2012;5 1:63-71. doi:10.1161/CIRCHEARTFAILURE.111.963462.
48. McDonagh TA, Metra M, Adamo M, Gardner RS, Baumbach A, Bohm M, et al. 2023 Focused Update of the 2021 ESC Guidelines for the diagnosis and treatment of acute and chronic heart failure. Eur Heart J. 2023;44 37:3627-39. doi:10.1093/eurheartj/ehad195.
49. Shah RU, Klein L and Lloyd-Jones DM. Heart failure in women: epidemiology, biology and treatment. Womens Health (Lond). 2009;5 5:517-27. doi:10.2217/whe.09.50.

50. Duan S, Li Y and Yang P. Predictive value of blood urea nitrogen in heart failure: a systematic review and meta-analysis. Front Cardiovasc Med. 2023;10:1189884. doi:10.3389/fcvm.2023.1189884.
51. Hunt SA, Abraham WT, Chin MH, Feldman AM, Francis GS, Ganiats TG, et al. ACC/AHA 2005 Guideline Update for the Diagnosis and Management of Chronic Heart Failure in the Adult—Summary Article. Circulation. 2005;112 12:1825-52. doi:10.1161/circulationaha.105.167587.
52. Masarone D, Martucci ML, Errigo V and Pacileo G. The Use of beta-Blockers in Heart Failure with Reduced Ejection Fraction. J Cardiovasc Dev Dis. 2021;8 9 doi:10.3390/jcdd8090101.
53. Kapelios CJ, Lund LH, Benson L, Dahlstrom U, Rosano GMC, Hauptman PJ, et al. Digoxin use in contemporary heart failure with reduced ejection fraction: an analysis from the Swedish Heart Failure Registry. Eur Heart J Cardiovasc Pharmacother. 2022;8 8:756-67. doi:10.1093/ehjcvp/pvab079.
54. Wang L, Liu W, He X, Chen Y, Lu J, Liu K, et al. Association of overweight and obesity with patient mortality after acute myocardial infarction: a meta-analysis of prospective studies. Int J Obes (Lond). 2016;40 2:220-8. doi:10.1038/ijo.2015.176.
55. D'Agostino RB, Belanger AJ, Kannel WB and Cruickshank JM. Relation of low diastolic blood pressure to coronary heart disease death in presence of myocardial infarction: the Framingham Study. BMJ. 1991;303 6799:385-9. doi:10.1136/bmj.303.6799.385.
56. Kim YJ, Lee G, Han J, Song K, Choi JS, Choi YL, et al. UBE2C Overexpression Aggravates Patient Outcome by Promoting Estrogen-Dependent/Independent Cell Proliferation in Early Hormone Receptor-Positive and HER2-Negative Breast Cancer. Front Oncol. 2019;9:1574. doi:10.3389/fonc.2019.01574.
57. Tiong KH, Tan BS, Choo HL, Chung FF, Hii LW, Tan SH, et al. Fibroblast growth factor receptor 4 (FGFR4) and fibroblast growth factor 19 (FGF19) autocrine enhance breast cancer cells survival. Oncotarget. 2016;7 36:57633-50. doi:10.18632/oncotarget.9328.
58. Bianchini G, De Angelis C, Licata L and Gianni L. Treatment landscape of triple-negative breast cancer - expanded options, evolving needs. Nat Rev Clin Oncol. 2022;19 2:91-113. doi:10.1038/s41571-021-00565-2.
59. Mihaly Z, Kormos M, Lanczky A, Dank M, Budczies J, Szasz MA, et al. A meta-analysis of gene expression-based biomarkers predicting outcome after tamoxifen treatment in breast cancer. Breast Cancer Res Treat. 2013;140 2:219-32. doi:10.1007/s10549-013-2622-y.
60. Kieu TL, Pierre L, Derangere V, Perrey S, Truntzer C, Jalil A, et al. Downregulation of Elovl5 promotes breast cancer metastasis through a lipid-droplet accumulation-mediated induction of TGF-beta receptors. Cell Death Dis. 2022;13 9:758. doi:10.1038/s41419-022-05209-6.
61. Kikuchi K, McNamara KM, Miki Y, Iwabuchi E, Kanai A, Miyashita M, et al. S100P and Ezrin promote trans-endothelial migration of triple negative breast cancer cells. Cell Oncol (Dordr). 2019;42 1:67-80. doi:10.1007/s13402-018-0408-2.
62. Maierthaler M, Kriegsmann M, Peng C, Jauch S, Szabo A, Wallwiener M, et al. S100P and HYAL2 as prognostic markers for patients with triple-negative breast cancer. Exp Mol Pathol. 2015;99 1:180-7. doi:10.1016/j.yexmp.2015.06.010.
63. Chen Q and Massague J. Molecular pathways: VCAM-1 as a potential therapeutic target in metastasis. Clin Cancer Res. 2012;18 20:5520-5. doi:10.1158/1078-0432.CCR-11-2904.
64. Chen Q, Zhang XH and Massague J. Macrophage binding to receptor VCAM-1 transmits survival signals in breast cancer cells that invade the lungs. Cancer Cell. 2011;20 4:538-49. doi:10.1016/j.ccr.2011.08.025.

65. Lu X, Mu E, Wei Y, Riethdorf S, Yang Q, Yuan M, et al. VCAM-1 promotes osteolytic expansion of indolent bone micrometastasis of breast cancer by engaging alpha4beta1-positive osteoclast progenitors. Cancer Cell. 2011;20 6:701-14. doi:10.1016/j.ccr.2011.11.002.
66. Lovelace TC, Dudek M, Fiore J and Benos PV. rCausalMGM: Scalable Causal Discovery and Model Selection on Mixed Datasets with 'rCausalMGM' [R package version 1.0.1]. Comprehensive R Archive Network (CRAN). 2026; doi:10.32614/CRAN.package.rCausalMGM.
67. Lovelace TC and Benos PV. Supporting data for "New causal discovery algorithm over censored variables identifies subtype-specific drivers of breast cancer progression". GigaScience Database. 2026; doi:10.5524/102819.
68. Li X, Hua C, Yue Q, Li Z, Tong J, Luo Z, Yang T, You L, Zhang H, Ma D, Wei X, Zhou H. GigaDB: A redesigned repository for data publishing and management. GigaScience. 2026. https://doi.org/10.1093/gigascience/giag047

FIGURE LEGENDS

Figure 1: Schematic illustrating the CausalCoxMGM method. CausalCoxMGM takes heterogeneous clinical and biomedical data including continuous, discrete, and censored variables as input. An interpretable causal probabilistic graphical model is then inferred in a two step process: (1) an undirected graphical model is learned with the proposed CoxMGM and (2) constraint-based causal discovery algorithms such as PC or FCI are applied to the undirected skeleton. The resulting causal graphical model can be used to construct parsimonious and robust predictors, and its interpretability aids hypothesis generation, patient stratification, and mechanism discovery.

Figure 2: Results for undirected graph adjacency recovery in simulated datasets. Area under the precision-recall curves (AUPRC) values are presented across all edges (ALL), continuous-censored edges (SC), and discrete-censored edges (SD). When sample size is varied, the number of features is 110 and degree is 4. When the number of features is varied, the sample size is 500 and the degree is 4. When the degree is varied, the sample size is 500 and the number of features is 110. Error bars denote 95% confidence intervals.

Figure 3: Results for directed graph adjacency recovery in simulated datasets. Area under the precision-recall curves (AUPRC) values are presented across all edges (ALL), continuous-censored edges (SC), and discrete-censored edges (SD). When sample size is varied, the number of features is 110 and degree is 4. When the number of features is varied, the sample size is 500 and the degree is 4. When the degree is varied, the sample size is 500 and the number of features is 110. Error bars denote 95% confidence intervals.

Figure 4: Results for CausalCoxMGM's causal structure recovery in simulated datasets. Values are presented across all edges (ALL), continuous-censored edges (SC), and discrete-censored edges (SD). (**A**) Orientation area under the precision-recall curves (AUPRC) across simulation conditions. (**B**) Normalized Structural Hamming Distance (SHD) across simulation conditions. When sample size is varied, the number of features is 110 and degree is 4. When the number of features is varied, the sample size is 500 and the degree is 4. When the degree is varied, the sample size is 500 and the number of features is 110. Error bars denote 95% confidence intervals.

Figure 5: Subtype-specific causal models reveals distinct gene expression drivers of breast cancer progression. (**A**) Subgraph of the CausalCoxMGM model for estrogen receptor positive (ER+) tumors containing censored outcomes and their MB. (**B**) Subgraph of the CausalCoxMGM model for estrogen receptor negative (ER-) tumors containing censored outcomes and their MB. Orange nodes are censored outcomes, green nodes are clinical features, and blue nodes are gene expression features. A directed edge $X \rightarrow Y$ indicates X is a cause of Y, a bidirected edge $X \leftrightarrow Y$ indicates X and Y have a shared latent confounder, a

partially oriented edge X o→ Y indicates Y is not a cause of X but it is unclear whether X causes Y or they share a latent confounder, and an unoriented edge X o-o Y indicates that causal orientation cannot be inferred for that edge. (**C**) Forest plots depicting the standardized hazard ratios (and 95% confidence intervals) from a multivariate Cox regression model for the MB of each mode of breast cancer progression. Standardized hazard ratios for features selected by both the ER+ (red) and ER- (blue) models are estimated in ER+ (right) and ER- (left) tumors. *Abbrev.* DSS: disease-specific survival; OD: death by other causes; DR: distant relapse; LR: locoregional relapse.

Figure 6: Predictive accuracy and feature selection parsimony in internal and external validation for CausalCoxMGM compared to LASSO Cox regression selected at the minimum deviance (Min) and one standard error rule (1SE) and Random Survival Forest (RSF) baselines. (**A**) Harrell's concordance statistic at 5, 10, and 15 years from baseline for each censored outcome estimated through 10-fold cross-validation. (**B**) Number of features selected by each predictive model for each outcome estimated through 10-fold cross-validation. (**C**) Harrell's concordance statistic at five years for common measures of breast cancer outcomes in METABRIC (estimated through 10-fold cross-validation) and an external Meta Cohort. Dashed lines at 0.5 denote expected concordance for random predictions. FDR adjusted p-values are given for all significant differences between CausalCoxMGM and baselines (FDR $p < 0.05$). *Abbrev.* DSS: disease-specific survival; OD: death by other causes; DR: distant relapse; LR: locoregional relapse; DRFS: distant relapse-free survival; DFS: disease-free survival.

CausalCoxMGM_Fig6
Click here to access/download;Figure;CausalCoxMGM_Fig6.pdf

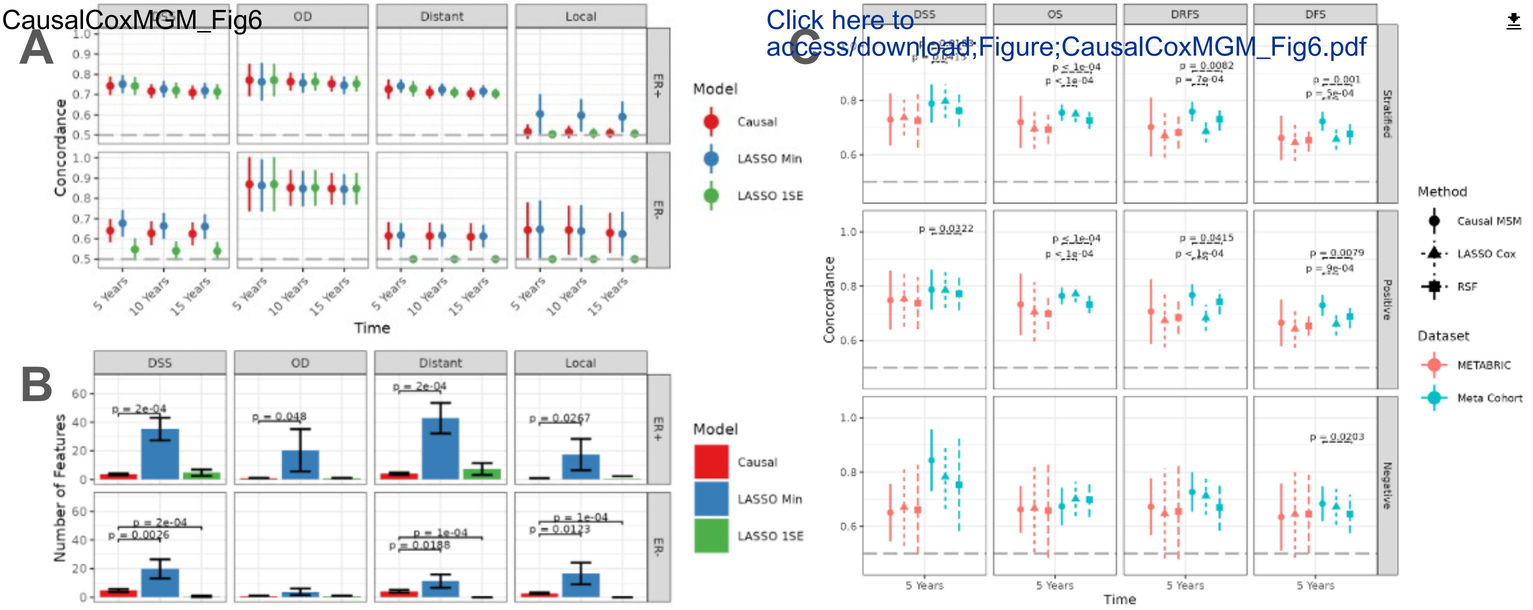

Figure 6: Predictive accuracy and feature selection parsimony in internal and external validation for CausalCoxMGM compared to LASSO Cox regression selected at the minimum deviance (Min) and one standard error rule (1SE) and Random Survival Forest (RSF) baselines. (**A**) Harrell's concordance statistic at 5, 10, and 15 years from baseline for each censored outcome estimated through 10-fold cross-validation. (**B**) Number of features selected by each predictive model for each outcome estimated through 10-fold cross-validation. (**C**) Harrell's concordance statistic at five years for common measures of breast cancer outcomes in METABRIC (estimated through 10-fold cross-validation) and an external Meta Cohort. Dashed lines at 0.5 denote expected concordance for random predictions. FDR adjusted p-values are given for all significant differences between CausalCoxMGM and baselines (FDR $p < 0.05$). *Abbrev.* DSS: disease-specific survival; OD: death by other causes; DR: distant relapse; LR: locoregional relapse; DRFS: distant relapse-free survival; DFS: disease-free survival.

CausalCoxMGM_Fig5
Click here to access/download;Figure;CausalCoxMGM_Fig5.pdf

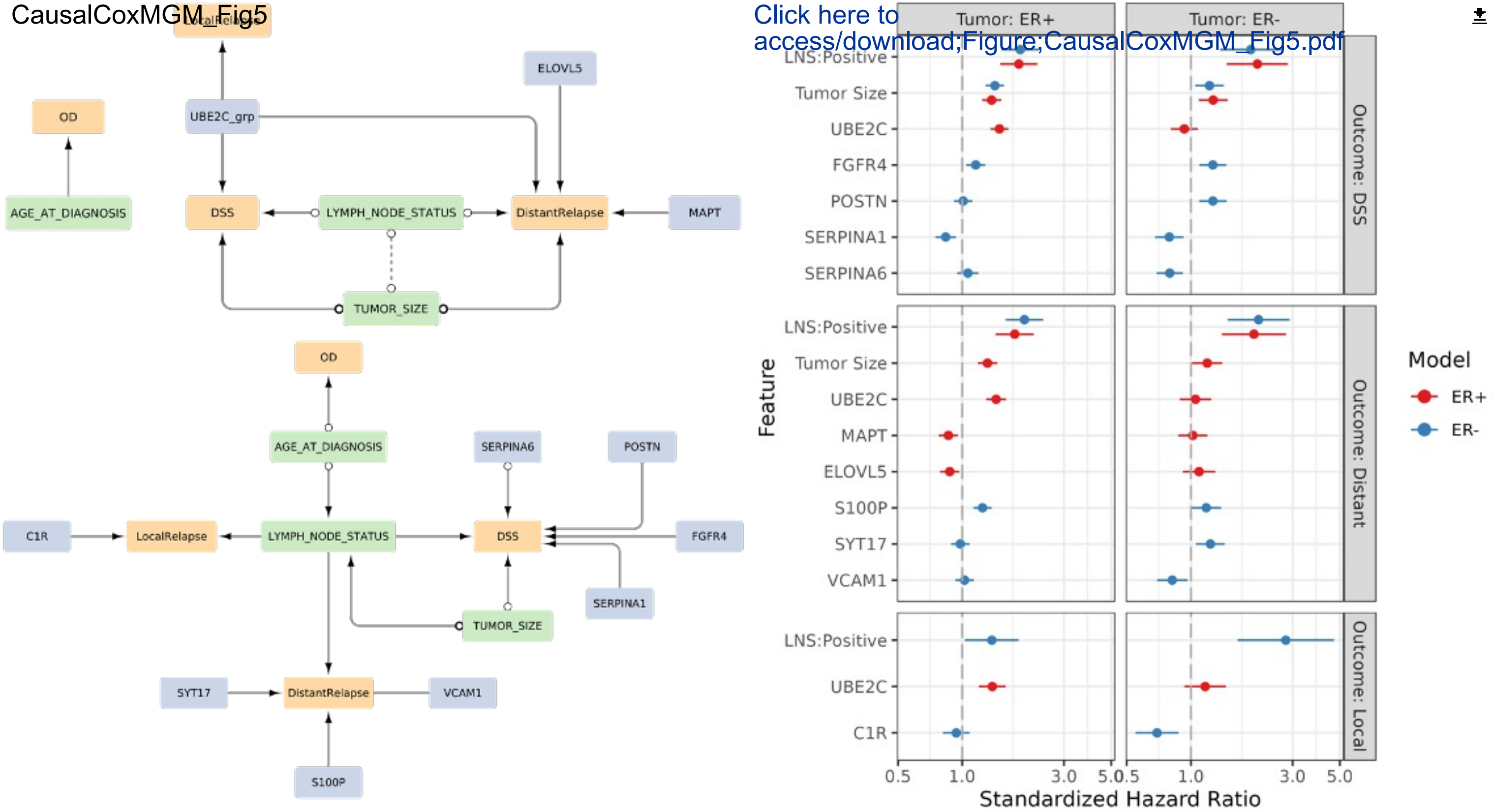

Figure 5: Subtype-specific causal models reveals distinct gene expression drivers of breast cancer progression. (**A**) Subgraph of the CausalCoxMGM model for estrogen receptor positive (ER+) tumors containing censored outcomes and their MB. (**B**) Subgraph of the CausalCoxMGM model for estrogen receptor negative (ER-) tumors containing censored outcomes and their MB. Orange nodes are censored outcomes, green nodes are clinical features, and blue nodes are gene expression features. (**C**) Forest plots depicting the standardized hazard ratios (and 95% confidence intervals) from a multivariate Cox regression model for the MB of each mode of breast cancer progression. Standardized hazard ratios for features selected by both the ER+ (red) and ER- (blue) models are estimated in ER+ (right) and ER- (left) tumors. *Abbrev.* DSS: disease-specific survival; OD: death by other causes; DR: distant relapse; LR: locoregional relapse.

CausalCoxMGM_Fig4
Click here to access/download;Figure;CausalCoxMGM_Fig4.pdf

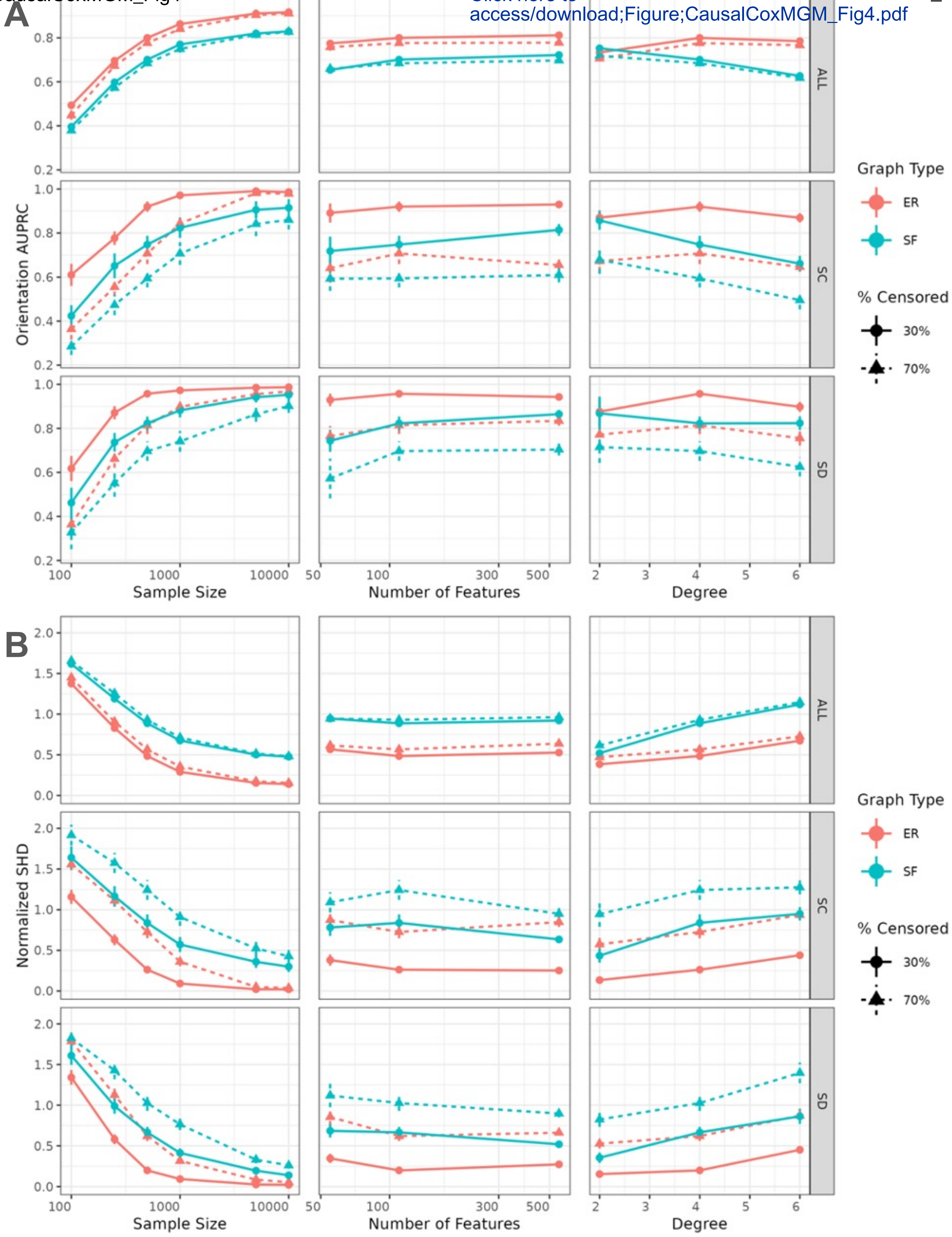

Figure 4: Results for CausalCoxMGM's causal structure recovery in simulated datasets. Values are presented across all edges (ALL), continuous-censored edges (SC), and discrete-censored edges (SD). (**A**) Orientation area under the precision-recall curves (AUPRC) across simulation conditions. (**B**) Normalized Structural Hamming Distance (SHD) across simulation conditions. When sample size is varied, the number of features is 110 and degree is 4. When the number of features is varied, the sample size is 500 and the degree is 4. When the degree is varied, the sample size is 500 and the number of features is 110. Error bars denote 95% confidence intervals.

CausalCoxMGM_Fig3

Click here to access/download;Figure;CausalCoxMGM_Fig3.pdf

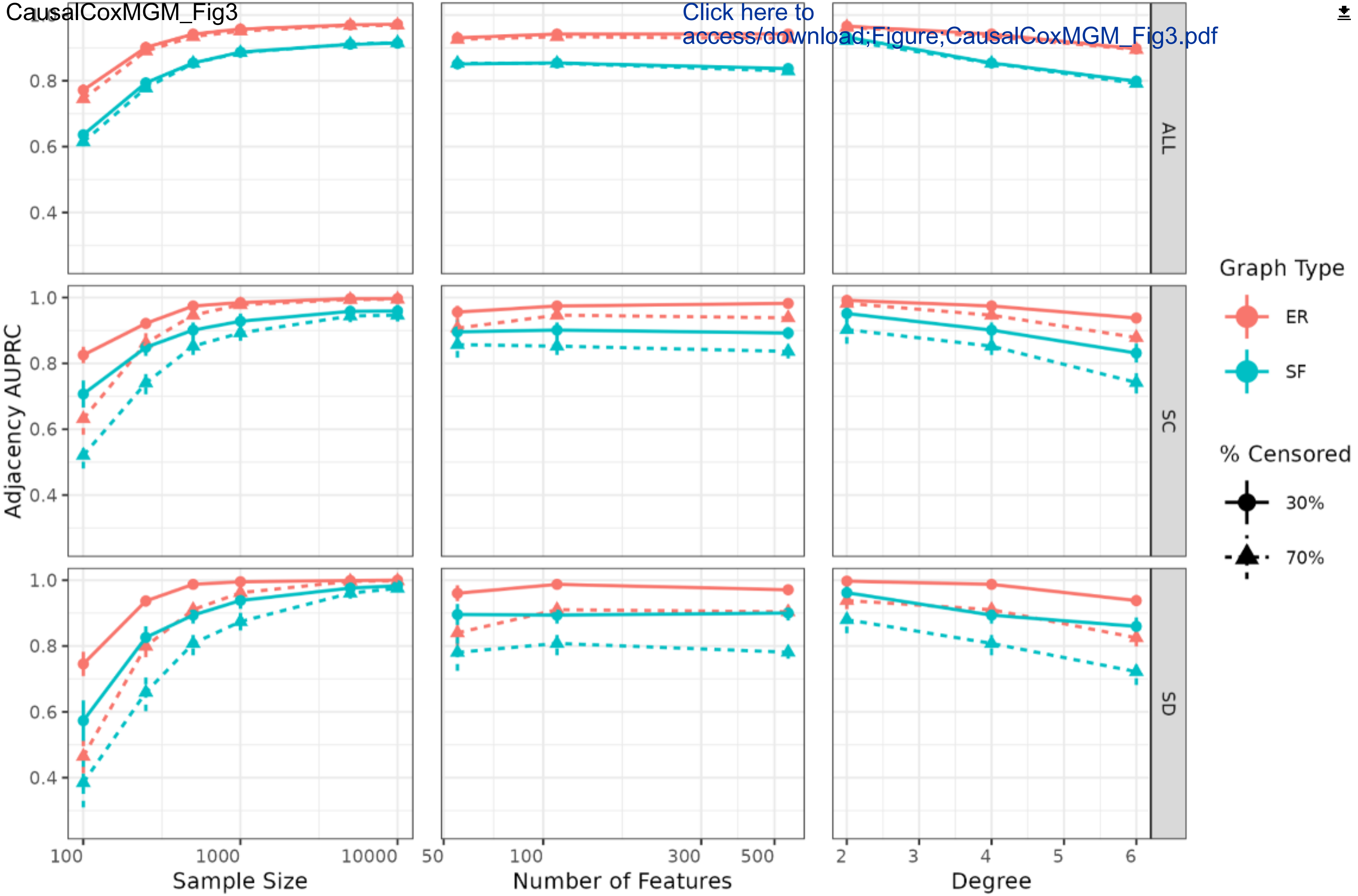

Figure 3: Results for directed graph adjacency recovery in simulated datasets. Area under the precision-recall curves (AUPRC) values are presented across all edges (ALL), continuous-censored edges (SC), and discrete-censored edges (SD). When sample size is varied, the number of features is 110 and degree is 4. When the number of features is varied, the sample size is 500 and the degree is 4. When the degree is varied, the sample size is 500 and the number of features is 110. Error bars denote 95% confidence intervals.

CausalCoxMGM_Fig2

Click here to access/download;Figure;CausalCoxMGM_Fig2.pdf

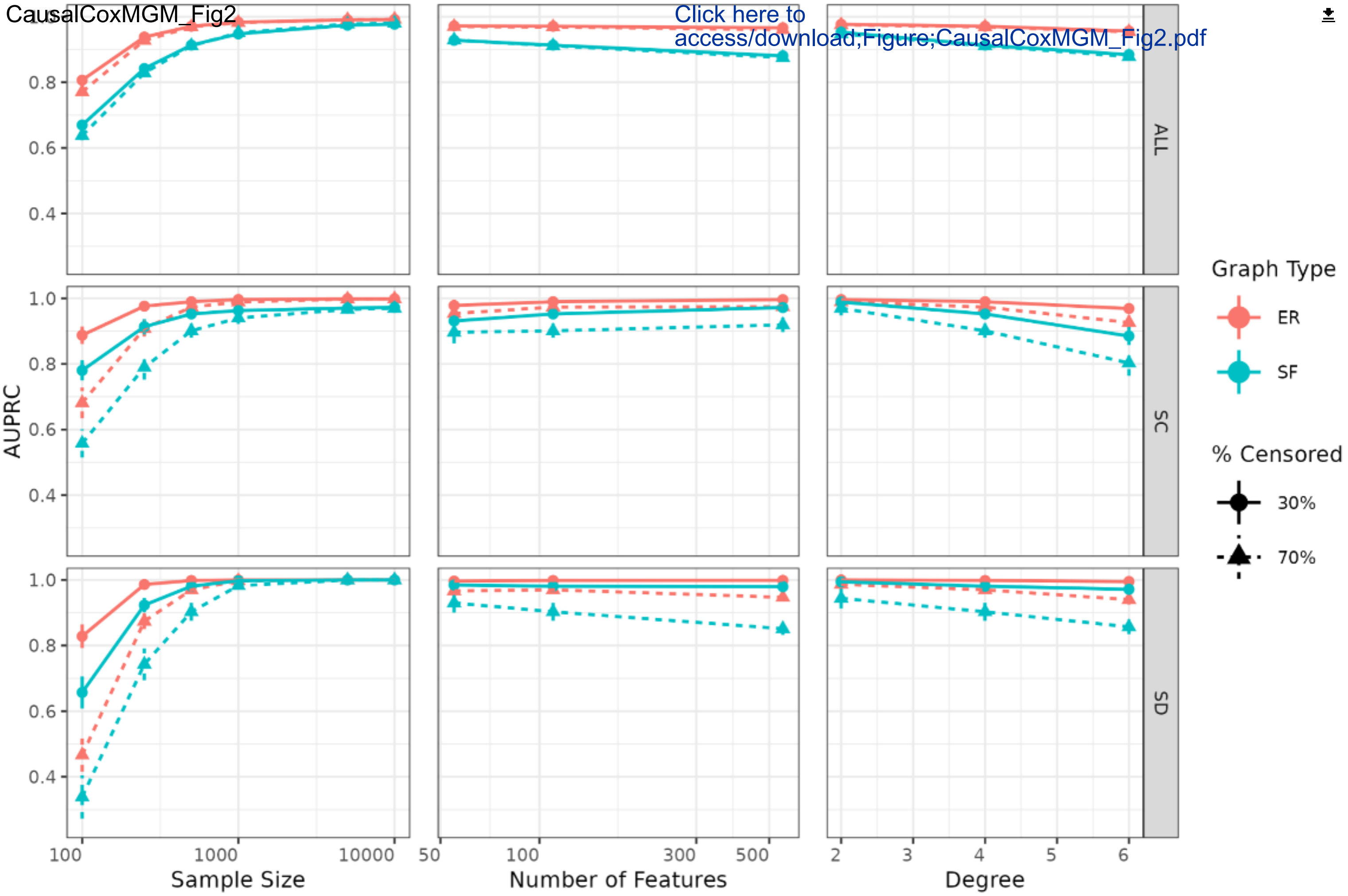

Figure 2: Results for undirected graph adjacency recovery in simulated datasets. Area under the precision-recall curves (AUPRC) values are presented across all edges (ALL), continuous-censored edges (SC), and discrete-censored edges (SD). When sample size is varied, the number of features is 110 and degree is 4. When the number of features is varied, the sample size is 500 and the degree is 4. When the degree is varied, the sample size is 500 and the number of features is 110. Error bars denote 95% confidence intervals.

Click here to access/download;Figure;CausalCoxMGM_Fig1.pdf

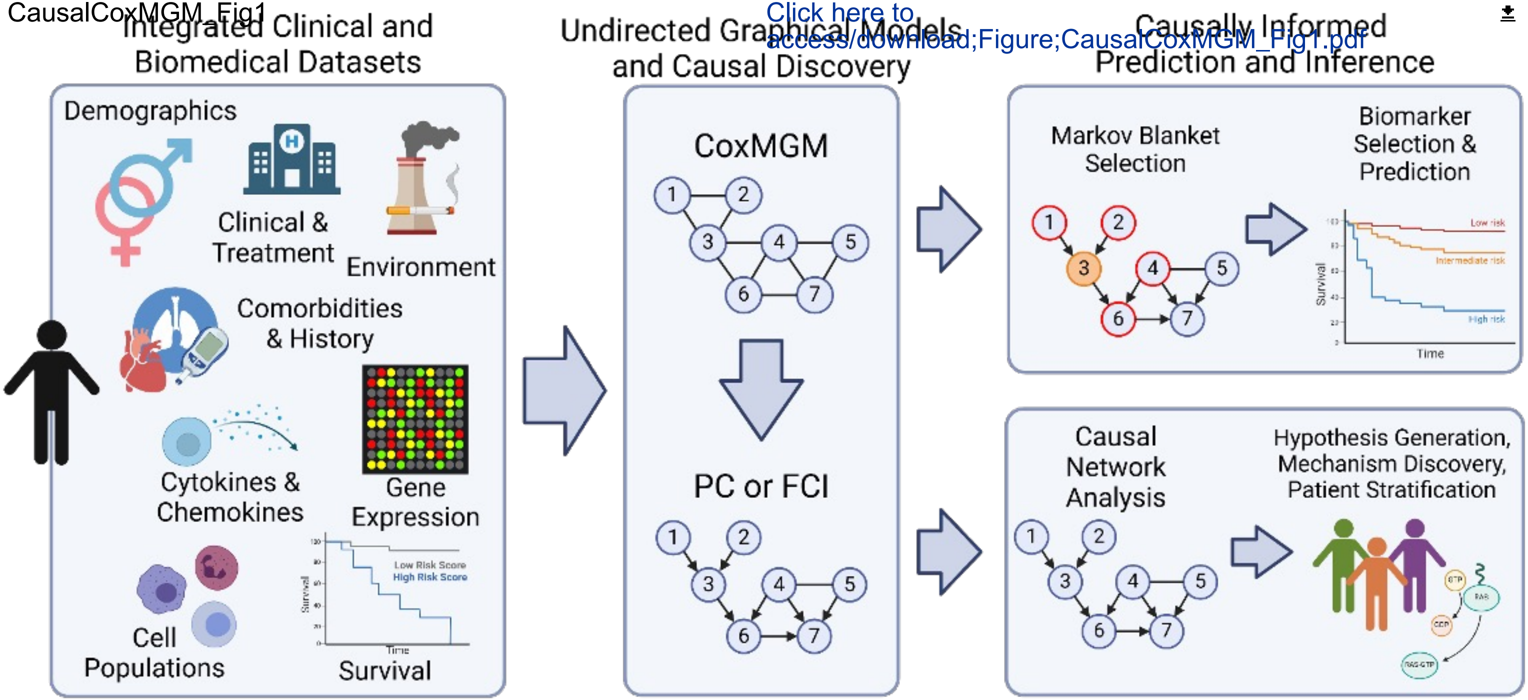

Figure 1: Schematic illustrating the CausalCoxMGM method. CausalCoxMGM takes heterogeneous clinical and biomedical data including continuous, discrete, and censored variables as input. An interpretable causal probabilistic graphical model is then inferred in a two step process: (1) an undirected graphical model is learned with the proposed CoxMGM and (2) constraint-based causal discovery algorithms such as PC or FCI are applied to the undirected skeleton. The resulting causal graphical model can be used to construct parsimonious and robust predictors, and its interpretability aids hypothesis generation, patient stratification, and mechanism discovery.

Click here to access/download
Supplementary Material
4a_CausalCoxMGM_Supplementary_Methods.2025020
4-pvb.docx

Click here to access/download
Supplementary Material
4b_CausalCoxMGM_Suppl_Figures.20250204-pvb.pdf

Click here to access/download
Supplementary Material
2_RESPONSE TO REVIEWERS.v2.1_tcl_pvb2.pdf

Cover letter for the Editor

Click here to access/download;Personal Cover;1_Cover Letter - Lovelace - GigaScience_R1.pdf

UF UNIVERSITY of FLORIDA

2004 Mowry Road
Gainesville, FL 32610

Email: pbenos @ ufl.edu
Tel: +1 352 273-5048
Fax: +1 352 273-5365

Panayiotis V. Benos Ph.D.
William Bushnell Presidential Chaired Professor

College of Public Health & Health Professions,
College of Medicine
Department of Epidemiology

August 22, 2025

Editorial Office
GigaScience

Dear Dr. Hans Zauner,

Thank you very much for giving us the opportunity to revise our manuscript titled "*New causal discovery algorithm over censored variables identifies subtype-specific drivers of breast cancer progression*".

We believe we have now addressed all reviewers' comments by adding more results (summarized in Suppl Fig. S7) and clarified a number of points. We hope that the reviewers will find the current version acceptable for publication.

Please do not hesitate to contact us if any additional information is needed.

Yours sincerely,

P. Benos

Panayiotis (Takis) Benos, PhD
William Bushnell Presidential Chaired Professor,
Department of Epidemiology, PHHP and COM, University of Florida
Tel.: 352-273-5048
e-mail: pbenos@ufl.edu
Lab URL: https://benos.epidemiology.phhp.ufl.edu

(on behalf of all authors)
