# Supplementary material for: New causal discovery algorithm over censored variables identifies subtype-specific drivers of breast cancer progression
Source: Gigascience. 2026 May 22;15:giag060. doi: 10.1093/gigascience/giag060 (PMC13235964; doi:10.1093/gigascience/giag060)
Supplement: giag060_Reviewer_1_Report_original_submission — Reviewer 1 -- 6/10/2025 [file giag060_reviewer_1_report_original_submission.pdf]

Reviewer Report

Title: New causal discovery algorithm over censored variables identifies subtype-specific drivers of breast cancer progression

Version: Original Submission **Date:** 6/10/2025

Reviewer name: Reviewer 1

Reviewer Comments to Author:

The manuscript presents a novel framework for causal discovery in datasets containing continuous, discrete, and censored variables. The method is validated through simulations and biomedical datasets, with a particular focus on breast cancer subtypes, and shows promise in identifying meaningful predictors of progression and survival.The paper closes an important methodological gap in the causal discovery framework, and it would be of great interest of the scientific community. However, to this reviewer, there are some important methodological concerns that should be addressed:1. While the authors provide extensive evaluation of discrimination (e.g., AUPRC, concordance index), no calibration analysis is presented. I would suggest the authors to include calibration metrics such as Brier scores, calibration plots (e.g., observed vs. predicted survival probabilities), or Hosmer-Lemeshow-type tests adapted for survival models. This will provide a more complete assessment of model performance, especially in clinical applications.2. The method's stability appears highly dependent on large sample sizes. In fact, performances drop considerably with small datasets, which is particularly relevant for research in rare cancers or low-frequency events. This limitation is noted in the discussion but should be made more explicit in the abstract and early results sections. Additionally, consider adding recommendations or guidelines for minimum sample sizes or strategies for small-sample settings (e.g., model regularization or data augmentation approaches).3. Missing data are excluded without any modeling or discussion of missingness mechanisms. This approach can introduce bias, especially if the data are not missing completely at random. The authors should clarify what missingness mechanisms are assumed and, ideally, implement multiple imputation or inverse probability weighting. Sensitivity analyses could be used to assess how robust the causal inferences are to different assumptions about missing data. This is particularly important for causal modeling, where biased exclusions can distort estimated relationships.Addressing these points would significantly strengthen the manuscript and increase its methodological rigor and practical relevance.

Level of Interest

Please indicate how interesting you found the manuscript: Choose an item.

Quality of Written English

Please indicate the quality of language in the manuscript: Choose an item.

Declaration of Competing Interests

Please complete a declaration of competing interests, considering the following questions:

- Have you in the past five years received reimbursements, fees, funding, or salary from an organisation that may in any way gain or lose financially from the publication of this manuscript, either now or in the future?
- Do you hold any stocks or shares in an organisation that may in any way gain or lose financially from the publication of this manuscript, either now or in the future?
- Do you hold or are you currently applying for any patents relating to the content of the manuscript?
- Have you received reimbursements, fees, funding, or salary from an organization that holds or has applied for patents relating to the content of the manuscript?
- Do you have any other financial competing interests?
- Do you have any non-financial competing interests in relation to this paper?

If you can answer no to all of the above, write 'I declare that I have no competing interests' below. If your reply is yes to any, please give details below.

'I declare that I have no competing interests' below

I agree to the open peer review policy of the journal. I understand that my name will be included on my report to the authors and, if the manuscript is accepted for publication, my named report including any attachments I upload will be posted on the website along with the authors' responses. I agree for my report to be made available under an Open Access Creative Commons CC-BY license (http://creativecommons.org/licenses/by/4.0/). I understand that any comments which I do not wish to be included in my named report can be included as confidential comments to the editors, which will not be published.

Choose an item.

To further support our reviewers, we have joined with Publons, where you can gain additional credit to further highlight your hard work (see: https://publons.com/journal/530/gigascience). On publication of this paper, your review will be automatically added to Publons, you can then choose whether or not to claim your Publons credit. I understand this statement.

Yes Choose an item.
